# *KRAS* mutations and endometriosis burden of disease

**DOI:** 10.1002/cjp2.317

**Published:** 2023-03-28

**Authors:** Natasha L Orr, Arianne Albert, Yang Doris Liu, Amy Lum, JooYoon Hong, Catalina L Ionescu, Janine Senz, Tayyebeh M Nazeran, Anna F Lee, Heather Noga, Kate Lawrenson, Catherine Allaire, Christina Williams, Mohamed A Bedaiwy, Michael S Anglesio, Paul J Yong

**Affiliations:** ^1^ Department of Obstetrics and Gynecology University of British Columbia Vancouver Canada; ^2^ BC Women's Centre for Pelvic Pain and Endometriosis BC Women's Hospital and Health Centre Vancouver Canada; ^3^ Women's Health Research Institute Vancouver Canada; ^4^ Department of Pathology and Laboratory Medicine University of British Columbia Vancouver Canada; ^5^ Department of Pathology and Laboratory Medicine BC Women's and Children's Hospital Vancouver Canada; ^6^ Women's Cancer Research Program at Samuel Oschin Comprehensive Cancer Institute Cedars‐Sinai Medical Center Los Angeles CA USA

**Keywords:** classification, endometrioma, endometriosis, ethnicity, fertility, gene, *KRAS*, pain, somatic mutation, surgery

## Abstract

The clinical phenotype of somatic mutations in endometriosis is unknown. The objective was to determine whether somatic *KRAS* mutations were associated with greater disease burden in endometriosis (i.e. more severe subtypes and higher stage). This prospective longitudinal cohort study included 122 subjects undergoing endometriosis surgery at a tertiary referral center between 2013 and 2017, with 5–9 years of follow‐up. Somatic activating *KRAS* codon 12 mutations were detected in endometriosis lesions using droplet digital PCR. *KRAS* mutation status for each subject was coded as present (*KRAS* mutation in at least one endometriosis sample in a subject) or absent. Standardized clinical phenotyping for each subject was carried out via linkage to a prospective registry. Primary outcome was anatomic disease burden, based on distribution of subtypes (deep infiltrating endometriosis, ovarian endometrioma, and superficial peritoneal endometriosis) and surgical staging (Stages I–IV). Secondary outcomes were markers of surgical difficulty, demographics, pain scores, and risk of re‐operation. *KRAS* mutation presence was higher in subjects with deep infiltrating endometriosis or endometrioma lesions only (57.9%; 11/19) and subjects with mixed subtypes (60.6%; 40/66), compared with those with superficial endometriosis only (35.1%; 13/37) (*p* = 0.04). *KRAS* mutation was present in 27.6% (8/29) of Stage I cases, in comparison to 65.0% (13/20) of Stage II, 63.0% (17/27) of Stage III, and 58.1% (25/43) of Stage IV cases (*p* = 0.02). *KRAS* mutation was also associated with greater surgical difficulty (ureterolysis) (relative risk [RR] = 1.47, 95% CI: 1.02–2.11) and non‐Caucasian ethnicity (RR = 0.64, 95% CI: 0.47–0.89). Pain severities did not differ based on *KRAS* mutation status, at either baseline or follow‐up. Re‐operation rates were low overall, occurring in 17.2% with *KRAS* mutation compared with 10.3% without (RR = 1.66, 95% CI: 0.66–4.21). In conclusion, *KRAS* mutations were associated with greater anatomic severity of endometriosis, resulting in increased surgical difficulty. Somatic cancer‐driver mutations may inform a future molecular classification of endometriosis.

## Introduction

Endometriosis is defined as the presence of endometrial‐like epithelial and stromal cells in extra‐uterine locations [1, 2, 3]. Endometriosis can be divided into three anatomical subtypes: deep infiltrating endometriosis (DIE), ovarian endometrioma (OMA), and superficial peritoneal endometriosis (SUP) [4]. Treatment includes hormonal suppression or surgical resection of endometriosis lesions. Surgical staging of endometriosis commonly utilizes the revised American Society for Reproductive Medicine (rASRM) classification, based on anatomical subtype amount and distribution, depth of endometriosis lesions, and presence of adhesions and OMAs [5]. The rASRM classification includes Stage I (minimal; score of 0–5), Stage II (mild; score of 6–15), Stage III (moderate; score of 16–40), and Stage IV (severe; score of >40) disease, where complete obliteration of the posterior cul‐de‐sac, commonly due to DIE, and large OMAs, are typically present [5, 6]. A diverse group of symptoms are associated with this condition including infertility, painful periods, sexual pain, and chronic pain. Despite ~190 million affected people worldwide, much remains to be understood about the fundamental molecular processes driving disease progression [4].

Though endometriosis is a benign condition, malignant transformation occurs in approximately 1% of cases, largely restricted to the OMA subtype and leading to endometriosis‐associated ovarian cancers [7]. Somatic cancer‐driver mutations have been described in endometriosis adjacent to endometriosis‐associated ovarian cancers, and have been proposed to be necessary but not sufficient for malignant transformation [8]. A growing literature has also identified somatic cancer‐driver mutations in endometriosis not associated with cancer, across anatomic subtypes and restricted to endometriotic epithelium [9, 10, 11, 12, 13, 14]. Somatic activating *KRAS* mutations appear to be the most common somatic mutations currently reported in endometriosis, ranging from 19.4 to 46.7% of cases based on previous literature [12, 15, 16, 17]. Endometriosis can have tumor‐like qualities such as local invasiveness, proliferation, resistance to apoptosis, and spread, which leads to more anatomically severe disease [12, 16, 17, 18, 19, 20]. Only recently, studies have begun to explore somatic cancer‐driver mutations and endometriosis phenotypes [17].

We recently reviewed the literature on somatic mutations and other somatic genomic alterations in endometriosis, and proposed a protocol for clinical phenotyping to facilitate reproducibility [21]. This protocol emphasizes pathology review and tissue enrichment given that endometriotic epithelium only forms a minority of surgically excised tissue specimens. Proposed clinical annotation in the protocol includes anatomic features, pain and fertility measures, and prospective follow‐up of outcomes.

In this study, we describe a prospective longitudinal cohort of patients undergoing surgery for endometriosis, with somatic activating *KRAS* codon 12 mutation testing in excised endometriosis lesions after pathology review and tissue enrichment. Subjects were followed 5–9 years after surgery, with detailed clinical annotation. The objective of the study was to characterize the clinical phenotype of *KRAS* mutations in endometriosis. We hypothesized that *KRAS* mutations would be associated with greater anatomic disease burden in endometriosis (i.e. more severe anatomic subtypes and higher stage).

## Materials and methods

### Cohort description

The setting of the study was the BC Centre for Pelvic Pain and Endometriosis, a tertiary referral center for endometriosis. Surgically excised endometriosis was prospectively biobanked beginning in 2013 (ENDOONC study; REB H11‐00536 and H14‐03040), while baseline and ongoing longitudinal follow‐up clinical data were collected as part of a prospective registry at our center (EPPIC registry, Clinicaltrials.gov # NCT02911090, REB H11‐02882, and H16‐00264) [22, 23]. The EPPIC registry systematically collected real‐time patient reported data (e.g. pain severities, ethnicity) and physician reported data (e.g. physical exam, recording of surgical procedures and findings). Standardized follow‐up in the EPPIC registry involved annual patient reported questionnaires to 2 years after baseline, as well as physician report of any repeat surgeries (re‐operations) at the center until study end in May 2022. Prospective consent was sought for both the biobank and the registry, which were linked to allow for correlation of somatic mutations with detailed phenotypic data.

Inclusion criteria were consecutive subjects with index surgeries (see Table 1 for list of procedures) at the center between 2013 and 2017 who gave informed consent to both biobanking and the registry. Subjects were excluded if they were post‐menopausal (spontaneous or surgical) or had a history of cancer or co‐existing cancer; and tissue samples were excluded from the *KRAS* mutation assay if they had insufficient endometriotic epithelial cells. See supplementary material, Figure S1 for the study flowchart.

**Table 1:** Patient and sample characteristics. Description of the study sample, including patient characteristics, findings at the index surgery, and procedures performed at the index surgery

Variable	Mean ± SD or No. (%)
*Patient characteristics*	
Age*	34.4 ± 6.6
Parity	
Parous	34 (27.9%)
Nulliparous	87 (71.3%)
Missing	1 (0.8%)
Ethnicity	
Caucasian only ethnicity	89 (73.0%)
Other ethnicity	28 (23.0%)
Mixed ethnicities	4 (3.3%)
Missing	1 (0.8%)
*Findings at index surgery*	
Anatomic subtype	
DIE only + OMA only	10/122 (8.2%) + 9/122 (7.4%)
SUP only	37/122 (30.3%)
Mixed (at least two different subtypes present)	66/122 (54.1%)
rASRM surgical staging	
I	29 (23.8%)
II	20 (16.4%)
III	27 (22.1%)
IV	43 (35.3%)
Missing	3 (2.4%)
OMA location	
Right	11 (18.6%)
Left	12 (20.3%)
Bilateral	22 (37.3%)
Location missing	14 (23.7%)
Cul‐de‐sac obliteration	
Complete	19 (15.6%)
Partial	28 (23.0%)
None	75 (61.5%)
DIE nodule location	
Posterior uterus/cervix	4 (7.0%)
Uterosacral	24 (42.1%)
Pelvic sidewall	5 (8.8%)
Colon	1 (19.3%)
Vaginal	3 (5.3%)
Bladder	1 (1.8%)
Appendix	0 (0%)
Location missing	9 (15.8%)
*Procedures at index surgery*	
Surgical approach	
Laparoscopy	121 (99.2%)
Laparotomy	1 (0.8%)
Excision of endometriosis (SUP, DIE)	
Yes	107 (87.7%)
No	15 (12.3%)
Hysterectomy	
Yes	33 (27.0%)
No	89 (73.0%)
Oophorectomy (e.g. for OMA)	
Right	13 (10.7%)
Left	9 (7.4%)
Bilateral	15 (12.3%)
No oophorectomy	85 (69.7%)
Ovarian cystectomy (OMA)	
Right	13 (10.7%)
Left	10 (8.2%)
Bilateral	11 (9.0%)
No cystectomy	88 (72.1%)
Ureteric surgery (ureterolysis)^†^	
Yes	63 (51.6%)
No	59 (48.4%)
Bowel surgery (shaving)^†^	
Yes	18 (14.8%)
No	104 (85.2%)
Bladder surgery (shaving)^†^	
Yes	2 (1.6%)
No	120 (98.4%)

* Age *n* = 121.

^†^ There were no cases requiring ureteric reimplantation, bowel resection, or bladder wall resection in this cohort.

### Specimen enrichment and DNA extraction

We employed a selective sampling strategy of the endometriosis lesions in each subject for the *KRAS* mutation assay. For subjects with DIE, all available DIE lesions were sampled for the *KRAS* mutation assay. For subjects with OMA, one OMA was sampled, plus an OMA on the contralateral ovary was also sampled if available. For subjects with SUP, one SUP lesion was sampled, plus a second SUP lesion at a different anatomical location was also sampled if available. Note that each subject could have one, two, or three anatomic subtypes concurrently. Each anatomic subtype was diagnosed by the surgeon (e.g. >5 mm invasion for DIE).

Samples were enriched by manual needle macrodissection or laser capture microdissection (LCM). Manual macrodissection was sufficient when the epithelial content of endometriosis lesions was sufficiently large (one or more clusters of epithelial glands appearing >1 mm^2^ on visual estimation) and there was limited surrounding tissue (i.e. lesion was close to the edge of the sample). We observed no difference in the rate of detectable mutations between LCM and macrodissection enrichment (supplementary material, Table S1). For each sample, the tissue was stained with hematoxylin and eosin (H&E) to identify endometriosis cells (i.e. pathology review). Sequential sections of a formalin‐fixed paraffin embedded block were then sectioned at 8 μm onto glass slides or polyethylene naphthalate membrane slides (Leica Microsystems Inc., Heerbrugg, Switzerland) for needle macrodissection or LCM, respectively. Tissue was deparaffinized with xylene and stained with 10% diluted H&E to enable capture with minimal surrounding fibrotic tissue. Manual macrodissection was done under a stereomicroscope with a 20‐gauge needle (Thermo Fisher Scientific, Waltham, MA, USA) [15]. LCM was performed on an LMD7000 (Leica Microsystems Inc., Switzerland) [12]. DNA was extracted using an Arcturus® PicoPure® DNA Extraction Kit (Thermo Fisher Scientific) and quantified using the Qubit 2.0 Fluorometer (Invitrogen; Thermo Fisher Scientific).

### *KRAS* codon 12 mutation assay: droplet digital PCR

DNA was pre‐amplified using primers flanking the codon 12 region (see Supplementary materials and methods). Droplet digital PCR (ddPCR), with a multiplex ddPCR screening assay to pre‐screen samples, was used to detect six *KRAS* codon 12 variants (c.34G>T (p.G12C), c.35G>A (p.G12D), c.34G>C (p.G12R), c.35G>T (p.G12V), c.35G>C (p.G12A), c.34G>A (p.G12S)). Empirical testing showed the combination of c.34G>T (p.G12C), c.35G>A (p.G12D), and c.34G>C (p.G12R) probes (Thermo Fisher Scientific) was sufficient to detect and resolve unique clusters for all *KRAS* variants (supplementary material, Figure S2). Any positive variant was confirmed with a variant‐specific ddPCR assay that was also used to establish variant allele frequency (Supplementary materials and methods; and supplementary material, Table S2). Note that enrichment of the endometriotic epithelium was not done (for LCM or macrodissection) and thus the variant allele frequency of the epithelial fraction is not known. Droplets were generated using a BioRad QX200 Automated Droplet Generator (BioRad Laboratories, Hercules, CA, USA), and quantified on a BioRad QX200 Droplet Reader (BioRad Laboratories). The mean detection thresholds and ranges for the individual *KRAS* assays in this study are listed in supplementary material, Table S3.

### Statistical analysis approach

For *KRAS* mutation status, the subjects were divided into two groups (present versus absent): present was defined as at least one *KRAS* mutation in at least one sample assayed in a subject, and absent was defined as the absence of *KRAS* mutations in all samples assayed in a subject.

The primary analysis was for an association between *KRAS* mutation status and anatomic phenotyping at the index surgery based on the anatomic subtypes of DIE, OMA, and SUP that were each confirmed on pathology. Subjects were phenotyped by (1) the presence of one or more subtypes and (2) by rASRM staging of the subtypes. Significance was set at *α* = 0.05 for the hypothesis that *KRAS* mutation would be associated with more severe anatomic disease. A subanalysis was carried out in the subgroup of subjects with only one sample assayed for *KRAS* mutation.

For secondary exploratory analyses, we examined for associations between the *KRAS* mutation variable and five types of outcome: other anatomic findings at the index surgery; markers of surgical difficulty; fertility variables at baseline; demographics at baseline; and pain variables at baseline. Mean differences and relative risks (RRs) are reported with 95% confidence intervals (CIs). For re‐operation during the follow‐up period, time to re‐operation based on *KRAS* mutation status was analyzed using Kaplan–Meier survival analysis with log‐rank test. For longitudinal follow‐up of pain scores after surgery based on *KRAS* mutation status, we evaluated the change in pain scores and also used linear regression for follow‐up pain scores while controlling for baseline pain scores.

Analyses were done using IBM SPSS Statistics 25 (SPSS Inc, Chicago, IL, USA). Missing data were assumed to be missing at random and excluded based by pairwise deletion (available‐case analysis). Data analysis was performed by NLO, AA, YDL, and PJY.

## Results

### Study sample

**Figure 1:**
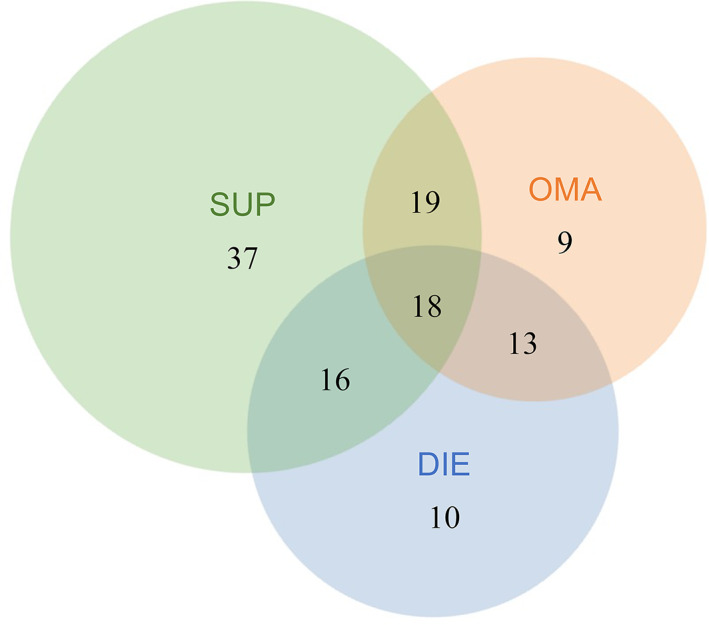
Cohort classification based on endometriosis anatomic subtype. Venn diagram of the 122 subjects in the study categorized by anatomic subtypes. Note that not all anatomic subtype lesions were sampled for *KRAS* mutation testing due to our selective sampling strategy (see Materials and methods section) and material availability constraints (lack of endometriotic epithelial cells).

This study included 122 subjects with a mean age of 34 ± 7 years. The distribution of subjects based on rASRM surgical stage was: Stage I – 23.8% (29/122), Stage II – 16.4% (20/122), Stage III – 22.1% (27/122), and Stage IV – 35.3% (43/122) (Table 1). The distribution of subjects based on anatomic subtype (each subtype confirmed as endometriosis on pathology) is illustrated in Figure 1, with half (54.1%; 66/122) of subjects having more than one subtype. Additional demographics for the cases are in supplementary material, Table S4.

### *KRAS* mutation prevalence

**Figure 2:**
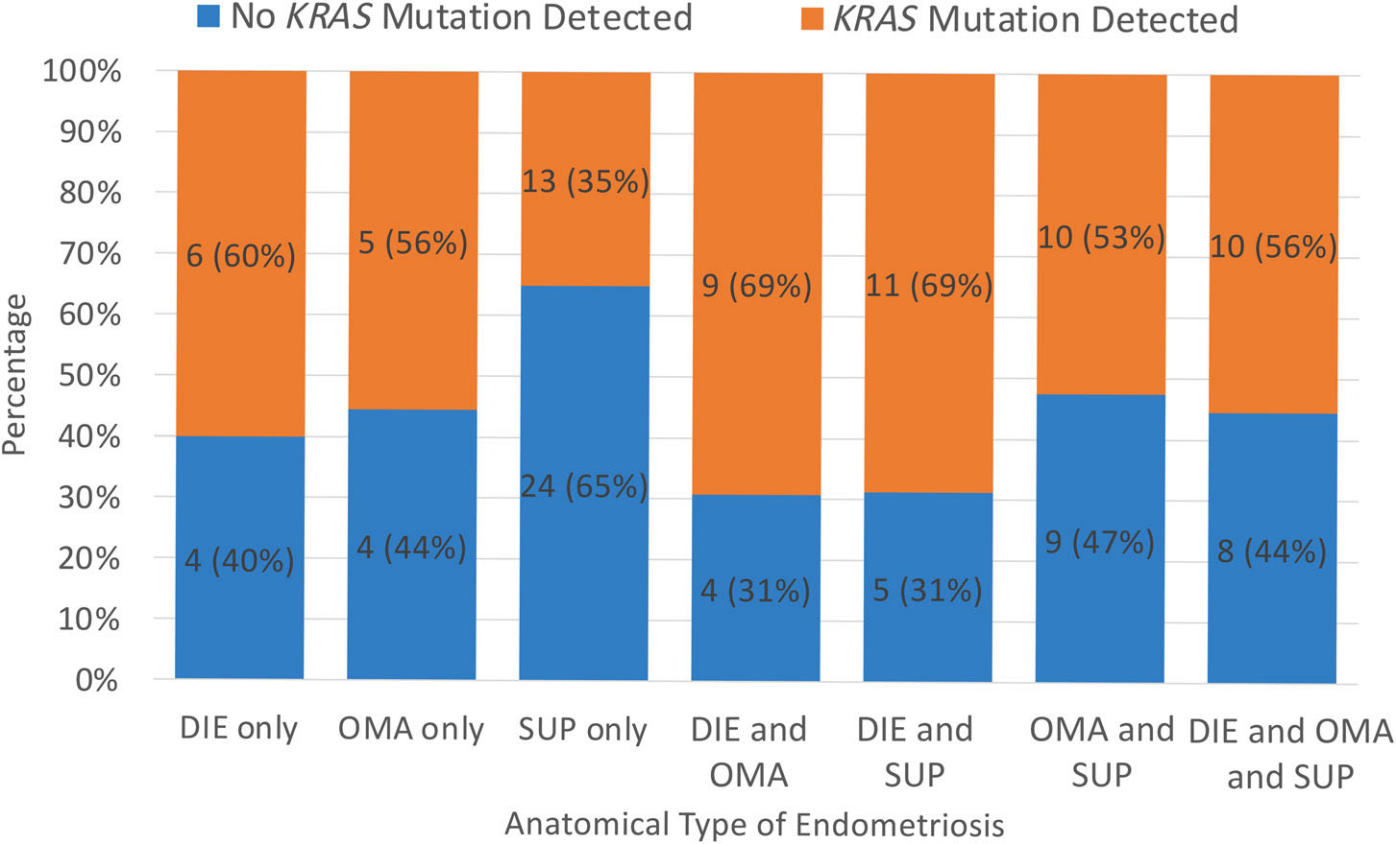
Prevalence of *KRAS* mutation based on anatomic subtypes. Number (percentage) of cases with *KRAS* mutation in the each of the following groups: subjects with DIE lesions only, OMA lesions only, SUP lesions only, and different combinations of anatomic subtypes.

From the 122 subjects, a total of 262 endometriosis lesions were sampled for the *KRAS* mutation assay based on our selective sampling strategy: 105 DIE samples, 44 OMA samples, and 113 SUP samples (supplementary material, Table S5). Most of the 122 subjects had one sample (*n* = 51) or two samples (*n* = 36) assayed for a mutation (range 1–7). In total, 52.5% (64/122) of the subjects had at least one endometriosis sample with a *KRAS* mutation, while 47.5% (58/122) had no *KRAS* mutations in any of the endometriosis samples assayed. The frequency of different codon 12 mutations was *KRAS* G12C (*n* = 4), *KRAS* G12D (*n* = 17), *KRAS* G12R (*n* = 5), *KRAS* G12V (*n* = 33), *KRAS* G12A (*n* = 11), and *KRAS* G12S (*n* = 7) – which includes 13 dual positives (i.e. individuals found to have more than one mutation). We observed the same *KRAS* G12 variant in more than one lesion in the same patient in eight (6.6%) patients, and *KRAS* G12D was more likely to be clonal in our cohort. The proportion of subjects with a *KRAS* mutation varied by which anatomic subtype(s) were present, with the lowest proportion in subjects with only SUP lesions (Figure 2).

### Primary analysis

For the primary outcome of anatomic phenotyping based on subtype, subjects with at least one *KRAS* mutation affected lesion were compared with subjects without mutation (Table 2). To meet assumptions of chi‐square testing, we categorized subjects as: (1) SUP only; (2) DIE only or OMA only; and (3) mixed (two or more) subtypes. *KRAS* mutation was present in 35.1% (13/37) of subjects with SUP lesions only, 57.9% (11/19) with DIE only or OMA only, and 60.6% (40/66) of those with mixed anatomic subtypes (chi‐square, *p* = 0.04) (Table 2). When the DIE or OMA only and the mixed subtypes were grouped together (versus SUP only cases), *KRAS* mutation had a RR of 1.36 (95% CI: 1.06–1.74) (Table 2), supporting an association between *KRAS* mutation and more anatomically severe disease.

**Table 2:** Primary analysis. Bivariate analyses for associations between *KRAS* mutation and the primary outcome of anatomic phenotyping of endometriosis

	*KRAS* mutation			
	No (*n* = 58)	Yes (*n* = 64)	*P* value*	RR^†^ (95% CI)
*Anatomic subtype*				
SUP only	24 (64.9%)	13 (35.1%)	0.04	RR for DIE or OMA only and mixed versus SUP only = 1.36 (1.06, 1.74)
DIE or OMA only	8 (42.1%)	11 (57.9%)		
Mixed	26 (24.2%)	40 (60.6%)		
*rASRM stage* ^‡^				
I	21 (72.4%)	8 (27.6%)	0.02	RR for Stage II–IV versus Stage I = 1.40 (1.12, 1.75)
II	7 (35.0%)	13 (65.0%)		
III	10 (37.0%)	17 (63.0%)		
IV	18 (41.9%)	25 (58.1%)		

* Chi‐square test for 2 × 3 table (anatomic subtype) and 2 × 4 table (stage).

^†^ RR for *KRAS* mutation as the ‘exposure’.

^‡^ Stage *n* = 119.

Similarly, a *KRAS* mutation was present in 27.6% (8/29) of rASRM Stage I, 65.0% (13/20) of Stage II, 63.0% (17/27) of Stage III, and 58.1% (25/43) of Stage IV cases (chi‐square, *p* = 0.02) (Table 2). When Stage II–IV cases were grouped together (versus Stage I cases), *KRAS* mutation had a RR of 1.40 (95% CI = 1.12–1.75) (Table 2), again supporting an association between *KRAS* mutation and more anatomically severe (advanced stage) disease.

A subanalysis was performed in those subjects that had only one sample assayed for mutation (*n* = 51). In this subgroup, *KRAS* mutation had an RR of 1.60 (95% CI = 0.98–2.60) for DIE or OMA only or mixed subtypes grouped together (versus SUP only cases). Likewise, in this subgroup, *KRAS* mutation had an RR of 1.60 (95% CI: 1.07–2.38) for Stage II–IV cases grouped together (versus Stage I cases).

### Secondary analyses

Secondary exploratory analyses are summarized in Table 3 for other anatomic findings at the index surgery, markers of surgical difficulty, fertility variables, demographics, and pain scores at baseline. *KRAS* mutation was associated with a higher risk of ureterolysis (retroperitoneal dissection of the ureter) being required during the index surgery (RR = 1.47, 95% CI: 1.02–2.12) (Table 3). We were limited in stratification of ethnicities with our cohort being predominantly Caucasian, and thus the sample was categorized into Caucasian versus other ethnicities (East and Southeast Asian ethnicities being the most prevalent [57.1%]). Subjects of Caucasian ethnicity were less likely to have a *KRAS* mutation (RR = 0.64, 95% CI: 0.47–0.89). There was no evidence of associations with other secondary outcomes (Table 3).

**Table 3:** Secondary analyses. Bivariate analyses for associations between *KRAS* mutation, and the following categories of variables: anatomic findings at the index surgery, difficulty of the index surgery, fertility variables, demographics, and pain scores

	*KRAS* mutation		
	No (*n* = 58)	Yes (*n* = 64)	RR or MD* (95% CI)
**Anatomic findings**			
*Cul‐de‐sac obliteration*			
Complete	8 (42.1%)	11 (57.9%)	RR for complete + partial versus no = 1.12 (0.71, 1.76)
Partial	13 (46.4%)	15 (53.6%)	
No	37 (49.1%)	38 (50.7%)	
*Size (cm) of left OMA* ^†^	5.1 ± 3.1	4.2 ± 2.0	MD = −0.9 (−3.1, 1.3)
*Size (cm) of right OMA* ^†^	4.0 ± 3.2	4.5 ± 2.4	MD = 0.5 (−2.6, 1.5)
*SUP lesion*			
Blue/black (typical)	17 (47.2%)	19 (52.8%)	RR = 1.17 (0.70, 1.94)
Other (atypical)	29 (53.7%)	25 (46.3%)	
**Markers of surgical difficulty**			
*Index surgery time* ^†^ *(min)*	120.9 ± 80.3	125.2 ± 57.4	MD = 4.30 (−21.9, 30.5)
*Ureterolysis*			
Yes	24 (38.1%)	39 (61.9%)	RR = 1.47 (1.02, 2.12)
No	34 (58.6%)	25 (39.1%)	
**Fertility variables**			
*Parity* ^†^			
Nulliparous	39 (44.8%)	48 (55.2%)	RR = 1.13 (0.90, 1.42)
Parous	19 (55.9%)	15 (44.1%)	
*Infertility* ^†^			
Yes	17 (38.6%)	27 (61.4%)	RR for yes versus no = 1.29 (0.89, 1.86)
No	13 (56.5%)	10 (43.5%)	
Never tried	28 (51.9%)	26 (48.1%)	
**Demographics**			
*Ethnicity* ^‡^			
Caucasian only	48 (53.9%)	41 (46.1%)	RR = 0.64
Other	8 (28.6%)	20 (71.4%)	(0.47, 0.89)
*Age* ^†^	34.8 ± 6.7	34.1 ± 6.5	MD = −0.70 (−3.08, 1.68)
*History of prior surgery*			
Yes	41 (55.4%)	33 (44.6%)	RR = 0.73 (0.55, 0.97)
No	17 (35.4%)	31 (64.6%)	
**Pain scores at baseline**			
Dysmenorrhea^†^ (0–10)	7.5 ± 2.8	7.3 ± 2.5	MD = −0.20 (−1.2, 0.80)
Deep dyspareunia^†^ (0–10)	6.4 ± 3.0	5.6 ± 3.4	MD = −0.80 (−1.98, 0.38)
Dyschezia (0–10)	4.8 ± 3.5	4.9 ± 3.1	MD = 0.1 (−1.1, 1.3)
Chronic pelvic pain (0–10)	6.8 ± 2.5	6.0 ± 2.9	MD = −0.80 (−1.78, 0.18)

MD, mean difference.

* RR for *KRAS* mutation as the ‘exposure’ (except for ethnicity, where Caucasian was considered the ‘exposure’). MD for continuous or 0–10 variables.

^†^ Left OMA *n* = 33, right OMA *n* = 34, index surgery time *n* = 114, parity *n* = 121, infertility *n* = 121, age *n* = 121, dysmenorrhea *n* = 110 (excludes those with no menses at baseline), deep dyspareunia *n* = 117 (excludes those not sexually active).

^‡^ Other (non‐Caucasian) ethnicities included: East or Southeast Asian (*n* = 16), South Asian (*n* = 6), Hispanic (*n* = 5), Other (*n* = 1). Mixed ethnicities group excluded.

For the assessment of re‐operation at the center, duration of prospective longitudinal follow‐up ranged from 5 to 9 years. The rate of re‐operation (for endometriosis) was low in the cohort overall (13.9%; 17/122), with 17.2% (11/64) of subjects with *KRAS* mutation and 10.3% (6/58) of subjects without *KRAS* mutation having a re‐operation (RR = 1.66, 95% CI: 0.66–4.21). The curve of re‐operation free survival appeared lower for subjects with *KRAS* mutation compared with subjects without *KRAS* mutation but with no statistically significant difference on Kaplan–Meier survival analysis log‐rank testing (supplementary material, Figure S3). For the 17 patients who have re‐operation data within our center, no patients have developed malignancies.

Pain scores (11‐point numeric rating scale [24]) were evaluated longitudinally after surgery at 2‐year follow‐up (or 1‐year follow‐up, if 2‐year data not available). Overall, pain scores decreased over time after surgery: dysmenorrhea decreased from 7.3 ± 2.6 to 3.6 ± 3.1, deep dyspareunia decreased from 6.2 ± 3.1 to 4.3 ± 3.2, dyschezia decreased from 4.9 ± 3.2 to 2.7 ± 2.5, and chronic pelvic pain decreased from 6.3 ± 2.7 to 3.5 ± 3.1. These changes over time were not associated with *KRAS* mutation status (supplementary material, Figure S4). Similarly, using linear regression with follow‐up pain score as the outcome (controlling for baseline pain), no associations with *KRAS* mutation were identified (supplementary material, Table S6).

## Discussion

### Principal findings

In this prospective longitudinal study of 122 subjects who underwent endometriosis surgery at a tertiary referral center, somatic *KRAS* codon 12 cancer‐driver mutations were associated with more anatomically severe endometriosis (i.e. those with DIE or OMA only or mixed subtypes, and higher rASRM stage).

### Results in the context of what is known

We detected *KRAS* mutation in 58–65% of the more anatomically severe cases. This figure is higher than previously reported for OMA (38.5–46.7%) or for DIE (19.4–33.3%) [12, 15, 16, 17, 25], likely due to sampling more lesions per case than in previous work or perhaps related to the patient population at our tertiary center likely representing more severe cases. Our mutation prevalence is also higher than prior studies utilizing lower resolution detection methods or without enrichment for endometriosis cells [9, 26].

### Clinical implications

The association between *KRAS* mutations and greater anatomic disease burden suggests that these activating mutations may play a role in lesion growth, invasion, or spread [13, 21]. We also noted an association between *KRAS* mutation and ureterolysis as a marker of surgical difficulty. This association with surgical difficulty is likely influenced by the association between *KRAS* mutation and advanced stage disease, as the latter often requires ureterolysis to separate the ureter from surrounding adhesions and fibrosis, prior to excision of endometriosis lesions.

*KRAS* mutations were less common among Caucasian subjects and more common among subjects of other ethnicities (predominantly East and Southeast Asian). Anatomically severe endometriosis was previously reported to be more common in East and Southeast Asian individuals in our registry [27]. It is possible that this epidemiological observation is accounted for by a higher rate of somatic events in *KRAS* in non‐Caucasian individuals with endometriosis, at least in our population. While intriguing, larger studies in other settings are needed to validate this possible association and investigate potential mechanisms.

No trends were observed for *KRAS* mutation status and baseline pain scores or change in pain scores over time. The lack of association is consistent with the marginal correlation between anatomic severity of endometriosis and pain symptoms, as the pathophysiology of endometriosis‐associated pain is multifactorial and can involve central sensitization [2, 3, 4, 28].

Re‐operation rates were overall low at this tertiary referral center for endometriosis (13.9% over 5–9 years of follow‐up). This may reflect the experience of high‐volume endometriosis surgeons at our center, though we cannot rule out the possibility that patients returned to and sought re‐operation in the community. A nonsignificant reduction in re‐operation free survival time after the index surgery was observed in those having a *KRAS* mutation. Since surgery involves excision of visible endometriosis disease, it is conceivable that microscopic residual endometriosis cells harboring *KRAS* mutations may be more likely to cause recurrent disease. However, confirmation of this difference would require a future study with larger sample size. Curiously, subjects with *KRAS* mutant endometriosis appeared to be less likely to have a prior surgery before the index surgery (Table 3). This may be an artefact of referral of suspected higher stage cases to our tertiary center, though biological influence cannot be ruled out and improved outcomes in some Ras‐harboring cancers have been reported [29, 30].

### Strengths and limitations

A major strength of this study is its prospective longitudinal design, with surveillance of re‐operation up to 5–9 years of follow‐up. Furthermore, registry data were entered in real time, ensuring higher accuracy and avoiding risk of bias from prior knowledge of mutation status. Moreover, highly sensitive ddPCR testing was employed, ensuring detection of subclonal alterations that have been reported previously, and enabling application of moderate enrichment methods that are operationally more feasible than laser capture or single‐cell molecular assays (e.g. macrodissection). However, we recognize that somatic alterations in *ARID1A, PIK3CA*, and other oncogenes and tumor suppressors have been reported and may coexist with our observed *KRAS* mutations contributing to clinical phenotypes and heterogeneity [12, 15, 17, 31]. Likewise, we tested only for the most common hot‐spot, *KRAS* codon 12 variants [21]. While reports of *KRAS* mutations outside of codon 12 alteration in endometriosis (and malignancies) are moderately rare [21] we cannot exclude the possibility that these may be present and not accounted for in our population. Whole genome or exome approaches [12, 14, 31] may also reveal complex genomic landscapes associated with clinical phenotypes. Due to enrichment via macrodissection in the majority of specimens in our study, we could not reliably test for associations between (increasing/decreasing) allele frequency and clinicopathological features. In particular, the use of macrodissection results in apparent low mutant allele frequencies (supplementary material, Table S5); allele frequencies would be higher if endometriotic epithelium were isolated by LCM.

It should be noted that we did not sample every lesion in each subject, but utilized a selective sampling strategy based on anatomic subtype. By definition, cases with more anatomically severe endometriosis will have more lesions to be sampled, and one would expect a greater probability of finding at least one mutation by chance with a higher number of samples assayed. For this reason, we performed a subanalysis in the subgroup with only one lesion sampled according to our sampling strategy, and observed increased RRs that were similar to the whole group analysis. Optimally, complete sampling of all lesions within each patient in future research would enable a per lesion analysis of mutation rate (versus the per subject analysis in this study). Validation of the findings in other cohorts is also required to confirm generalizability. In addition, our cases are from a tertiary referral center and thus the results cannot be reliably extrapolated to the general population.

### Research implications

We hypothesize that somatic activating *KRAS* codon 12 mutations may contribute to constitutive activation in downstream pathways that results in proliferation, local invasion, and metastatic spread resulting in advanced stage disease with elevated surgical complexity [17, 21]. Models of endometriosis, in particular whole‐animal models, will be necessary to investigate putative mechanisms [32, 33, 34, 35, 36, 37]. While targeting of *KRAS* has historically been challenging, new opportunities have been presented including variant‐specific agents and synthetic lethal strategies [38, 39, 40]. Work presented here suggests the *KRAS* axis may be a relevant target to reduce the invasiveness, spread, or burden of endometriosis.

Furthermore, somatic mutations such as in *KRAS* may inform a novel molecular classification of endometriosis. This future molecular classification may incorporate other somatic genomic alterations, germline polymorphisms, RNA expression changes, immunohistochemistry markers, together integrated with clinical variables. Ultimately, a clinically useful molecular classification for endometriosis should demonstrate correlations with baseline phenotype and response to treatment. In the future, molecular subtypes of endometriosis could be built into clinical trial design and subtype‐specific treatments could be incorporated into care.

## Conclusions

*KRAS* somatic‐cancer driver mutations were associated with greater anatomic disease burden in endometriosis and thus more surgical complexity. *KRAS* mutations may serve as a nonhormonal therapeutic target and contribute to a molecularly informed classification in endometriosis.

## Author contributions statement

NLO, AFL, MAB, MSA and PJY conceptualized the study. NLO, AL, JH, CLI, JS and TMN carried out experiments. NO, TMN, AL, KL, MSA and PJY designed the methodology and curated the data. NOL, AA, YDL and PJY analyzed the data. AFL, MAB, MSA and PJY acquired funding. CA, CW, MAB, MSA and PJY provided study resources. HN led project administration. NLO, MSA and PJY wrote the original draft. All authors were involved in reviewing, editing and final approval of the manuscript.

## Supporting information


Supplementary materials and methods
**Figure S1.** Study flowchart
**Figure S2.** Multiplex assay with all six *KRAS* codon 12 positive controls, each with a unique clustering region
**Figure S3.** Kaplan–Meier survival analysis
**Figure S4.** Change in pain scores between baseline and follow‐up, based on mutation status
**Table S1.** Comparison between manual microdissection and laser capture microdissection
**Table S2.** Cell line controls
**Table S3.** Mean detection thresholds for the individual *KRAS* assays
**Table S4.** Demographics
**Table S5.** Summary of mutation calls
**Table S6.** The association between follow‐up pain scores and *KRAS* mutation status, controlling for baseline pain severity – linear regression

## Data Availability

The participants of this study did not give written consent for their data to be shared publicly.
