# Supplementary material for: KRAS mutations and endometriosis burden of disease
Source: J Pathol Clin Res. 2023 Mar 28;9(4):302–12. doi: 10.1002/cjp2.317 (PMC10240146; doi:10.1002/cjp2.317)
Supplement: Supplementary file 1 — Supplementary materials and methods Figure S1. Study flowchart Figure S2. Multiplex assay with all six KRAS codon 12 positive controls, each with a unique clustering region Figure S3. Kaplan–Meier survival analysis Figure S4. Change in pain scores between baseline and follow‐up, based on mutation status Table S1. Comparison between manual microdissection and laser capture microdissection Table S2. Cell line controls Table S3. Mean detection thresholds for the individual KRAS assays Table S4. Demographics Table S5. Summary of mutation calls Table S6. The association between follow‐up pain scores and KRAS mutation status, controlling for baseline pain severity – linear regression [file CJP2-9-302-s001.pdf]

KRAS mutations and endometriosis burden of disease

NL Orr *et al*. *J Pathol Clin Res* https://doi.org/10.1002/cjp2.317

Supplementary Material

Reference numbers refer to the list in the main paper

Contents

Supplementary Materials and Methods…………………………………. ………………………………………………………………………………**2**

Primer and probe sequences……………………………………………………………………………...*2*

Pre-amplification and droplet digital PCR – reaction procedure………………………………………………...*2*

Threshold for ddPCR………...*3*

Supplementary Figures……. …………………………………………………………………………………………………**4**

Figure S1. Study flowchart……………………………………………………………………………………………………..*4*

Figure S2. Multiplex assay with all six KRAS codon 12 positive controls, each with a unique clustering region………………………………………*5*

Figure S3. Kaplan Meier Survival Analysis…………………………………………………………………………………………*6*

Figure S4. Change in pain scores between baseline and follow-up, based on mutation status……………………………………………...………. *.7*

Supplementary Tables……………………………………………..……………………………………………………………..……**8**

Table S1. Comparison between manual microdissection and laser capture microdissection……………………………………………………...*8*

Table S2. Cell line controls..………………………………………………………………………………………………….…*9*

Table S3. Mean detection thresholds for the individual KRAS assays..…………………………………………………………………….....*10*

Table S4. Demographics…………………………………………………………………………………………………….. *11*

Table S5. Summary of mutation calls..……………………………………………………………………………………….....*13*

Table S6. The association between follow-up pain scores and KRAS mutation status, controlling for baseline pain severity – linear regression..….. *..70*

Supplementary Materials and Methods

Primer and probe sequences

Primer					
Assay	***Description***	***Primer Sequence***	***Source***		
KRAS G12	Forward	5'-GCCTGCTGAAAATGACTGAATATAAACT-3'	Applied Biosystems, Inc., USA		
	Reverse	5'-GCTGTATCGTCAAGGCACTCTT -3'	Applied Biosystems, Inc., USA		
**Probes**					
Assay	***Description***	***Probe Details***	***Source***	***Extension Temperature (ºC)***	***Assay Specifications***
KRAS Multiplex	Equal parts of G12C/ G12D/ G12R probes		In house	60	Equal parts G12C, G12D, and G12R probes at 40X
KRAS G12C (c.34G>T)	Wildtype	5'-VIC-TTGGAGCTGGTGGCGTA-NFQ-3'	Applied Biosystems, Inc., USA	60	Assay at 40X - use 1x primer/probe mix
	Mutant	5'-FAM-TTGGAGCTTGTGGCGTA-NFQ-3'			
KRAS G12D (c.35G>A)	Wildtype	5'-VIC-TTGGAGCTGGTGGCGTA-NFQ-3'	Applied Biosystems, Inc., USA	60	Assay at 40X - use 1x primer/probe mix
	Mutant	5'-FAM-TTGGAGCTGATGGCGTA-NFQ-3'			
KRAS G12R (c.34G>C)	Wildtype	5'-VIC-TTGGAGCTGGTGGCGTA-NFQ-3'	Applied Biosystems, Inc., USA	60	Assay at 40X - use 1x primer/probe mix
	Mutant	5'-FAM-TTGGAGCTCGTGGCGTA-NFQ-3'			
KRAS G12V (c.35G>T)	Wildtype	5'-Yak Yellow-CGCC+A+C+CA+GCT-IABkFQ-3'	Integrated DNA Technologies, Inc., USA	60	500nM primer + 200nM probe
	Mutant	5'-FAM-CG+CC+A+A+CAGC+TC-IABkFQ-3'			
KRAS G12A (c.35G>C)	Wildtype	5'-Yak Yellow-CGCC+A+C+CA+GCT-IABkFQ-3'	Integrated DNA Technologies, Inc., USA	60	500nM primer + 200nM probe
	Mutant	5'-FAM-CGCC+A+G+CA+GCT-IABkFQ-3'			
KRAS G12S (c.34G>A)	Wildtype	5'-Yak Yellow-CGCC+A+C+CA+GCT-IABkFQ-3'	Integrated DNA Technologies, Inc., USA	60	500nM primer + 200nM probe
	Mutant	5'-FAM-CGC+CA+C+T+AGC-IABkFQ-3'			

Pre-amplification and droplet digital PCR – reaction procedure

The pre-amplification reactions were denatured at 95ºC for 10 minutes, and cycled (94ºC for 30 seconds, then 60ºC for 4 minutes) for 10 cycles, followed by 4ºC hold.

For ddPCR: after droplet generation the reactions were denatured at 95ºC for 10 minutes and cycled (94ºC for 30 seconds then 60ºC for 90 seconds) at a 2.5C/sec ramp rate for 40 cycles; followed by 98ºC for 10 minutes and final hold at 4ºC.

Threshold for ddPCR

Once the samples had been read on the BioRad QX200 Droplet Reader, the number of droplets were reviewed to ensure there were, at minimum, 10,000 total droplets and 5,000 positive droplets (mutation (MUT) only + wildtype (WT) only). The sample was repeated if the threshold was not met. If the repeated run had above 1000 positive droplets and the results were consistent with the previous run, then we included the sample. Alternatively, if the sample was run twice on the same plate and the total added droplets were above the cut-off, then the average VAF of the two samples was used. If the sample continued to have low droplet counts, it was considered to have "failed" the assay and was excluded.

Interpretation of the ddPCR data and mutation calling involved the calculation of a VAF cut-off/threshold based on the results from the negative controls (endometriosis samples with no *KRAS* mutations), as previously described [12, 15].

For the multiplex assay, the average VAF for the negative controls multiplied by three was used as the threshold, except for the c.35G>T (p.G12V) probe, since mutant droplets appear in a region that is difficult to distinguish from the "no DNA" and "WT only" droplets. Thus, the threshold VAF was set lower (the average of the negative controls without multiplication by three) for c.35G>T (p.G12V) calls on the multiplex assay to avoid false negatives. As the multiplex assay assesses all six variants (see **Figure S2**), only mutant droplet clusters within the specific variant region of interest were considered for the VAF calculation of that variant. For example, if mutant droplets are identified in the c.35G>A (p.G12D) region and the c.34G>A (p.G12S) region for an individual sample, the VAF cut-off for the c.35G>A (p.G12D) variant will be calculated using mutant droplets only in the c.35G>A (p.G12D) region (i.e., the droplets within the c.34G>A (p.G12S) region are not included as mutant droplets in the calculation for VAF threshold of the c.35G>A (p.G12D) variant). Dual positive droplets were not included as mutant in the VAF calculation due to the non-specific clustering of these droplets (i.e., you cannot identify which variant is mutated).

In all cases the "positive" call was confirmed using variant specific probes in an individual (single-plex) assay. The average VAF for the negative controls multiplied by three was used as a threshold for all variants.

If the VAF from the sample was higher than the threshold VAF, the sample was called positive (i.e. mutation detected).

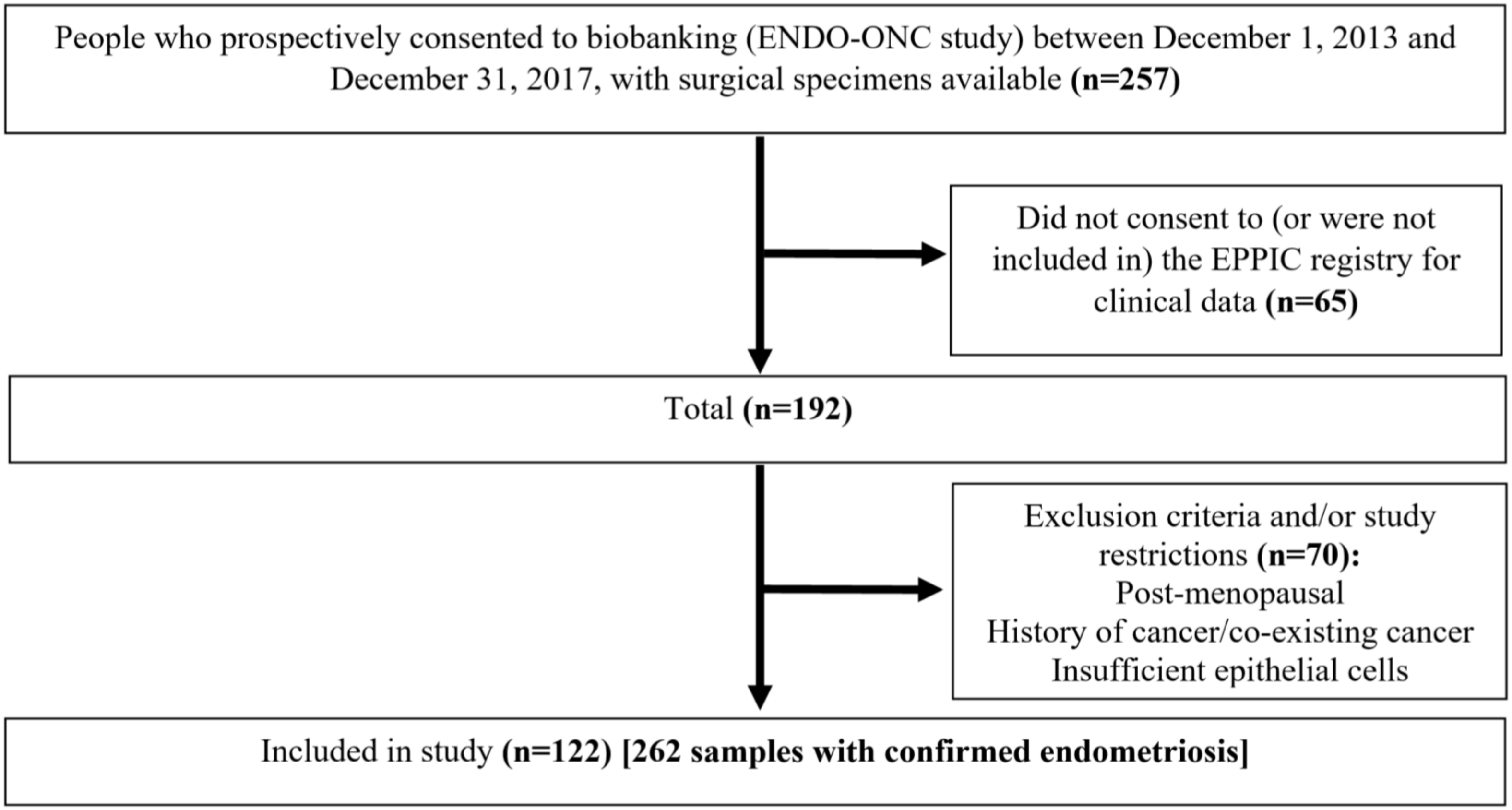

Figure 1. Study flowchart.

Flow chart of included and excluded cases. Subjects consented to both prospective biobanking and the prospective EPPIC registry for clinical data. Exclusions were both clinical (e.g. history of co-existing cancer, in order exclude cases of malignant transformation) and technical (e.g. insufficient endometriosis epithelial cells in the tissue block, due to epithelium-restricted *KRAS* mutations). 122 subjects were included in the study, with 262 samples from the subjects based on our mutation testing strategy (see "Specimen Enrichment and DNA Extraction" under the "Materials and Methods" section).

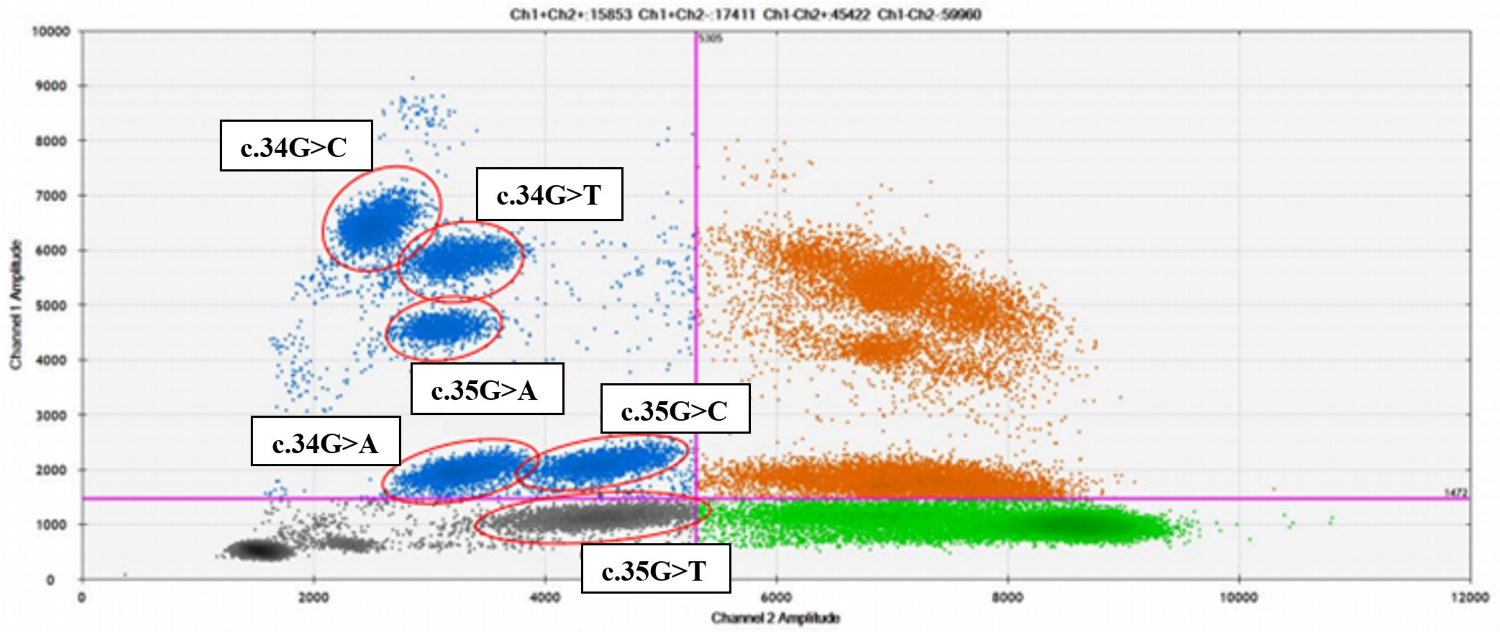

Figure 2. Multiplex assay with all six *KRAS* codon 12 positive controls, each with a unique clustering region.

The y-axis represents fluorescence of the FAM channel and the x-axis represents fluorescence of the VIC channel. The droplets containing mutant only DNA are shown in blue droplets on the top left quadrant; wildtype only DNA shown in the green droplets on the bottom right quadrant; dual positive droplets (both mutant and wildtype DNA present within the droplet) shown in the orange droplets at the top right quadrant; double negative droplets (no DNA) shown in the gray droplets in the bottom left quadrant. The pink line represents the threshold set to define each class of droplets. The regions of individual *KRAS* codon 12 variants are circled in red. c.34G>T: p.G12C; c.35G>A: p.G12D; c.34G>C: p.G12R; c.35G>T: p.G12V; c.35G>C: p.G12A; c.34G>A: p.G12S.

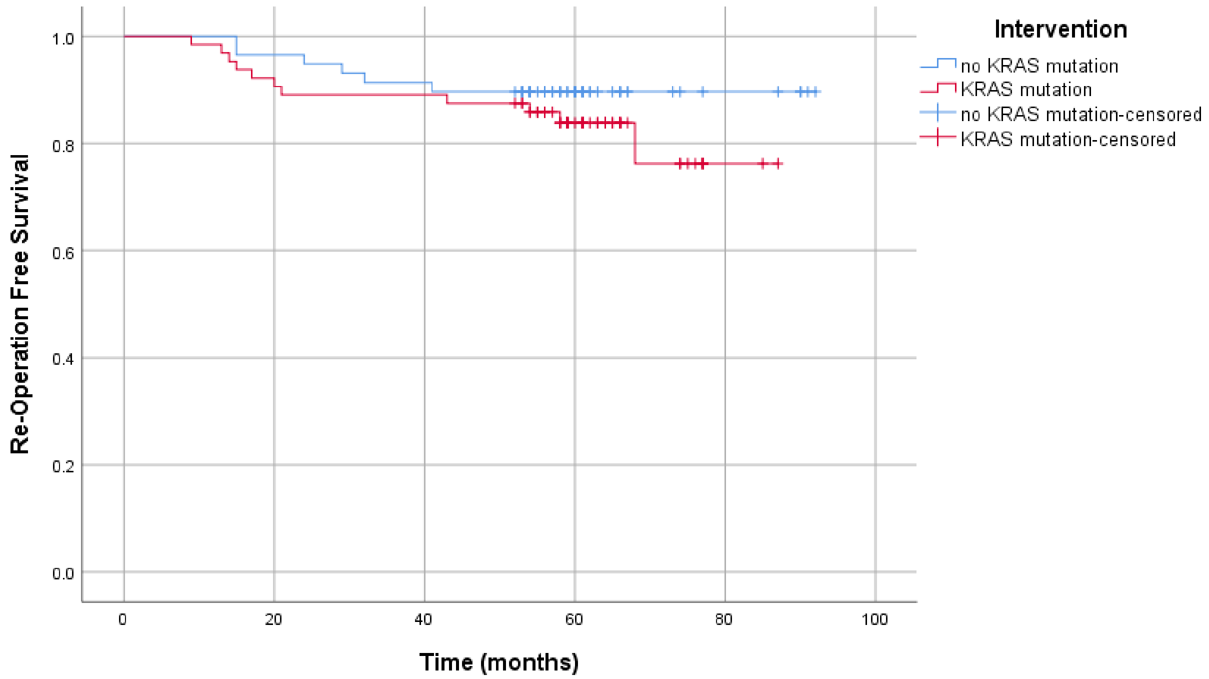

Number of remaining cases at each time

58	56	53	23	5	No *KRAS* mutation
64	58	57	31	2	*KRAS* mutation

Figure 3. Kaplan Meier Survival Analysis.

Kaplan Meier curves for re-operation free survival based on *KRAS* mutation status. Re-operation occurred in 17·2% (11/64) of cases with *KRAS* mutation compared to 10·3% (6/58) of cases without *KRAS* mutation (RR = 1·66, 95% CI: 0·66-4·21). Cases were censored at end of the follow-up period (May 2022). Log-rank test for *KRAS* mutation, p = 0·30.

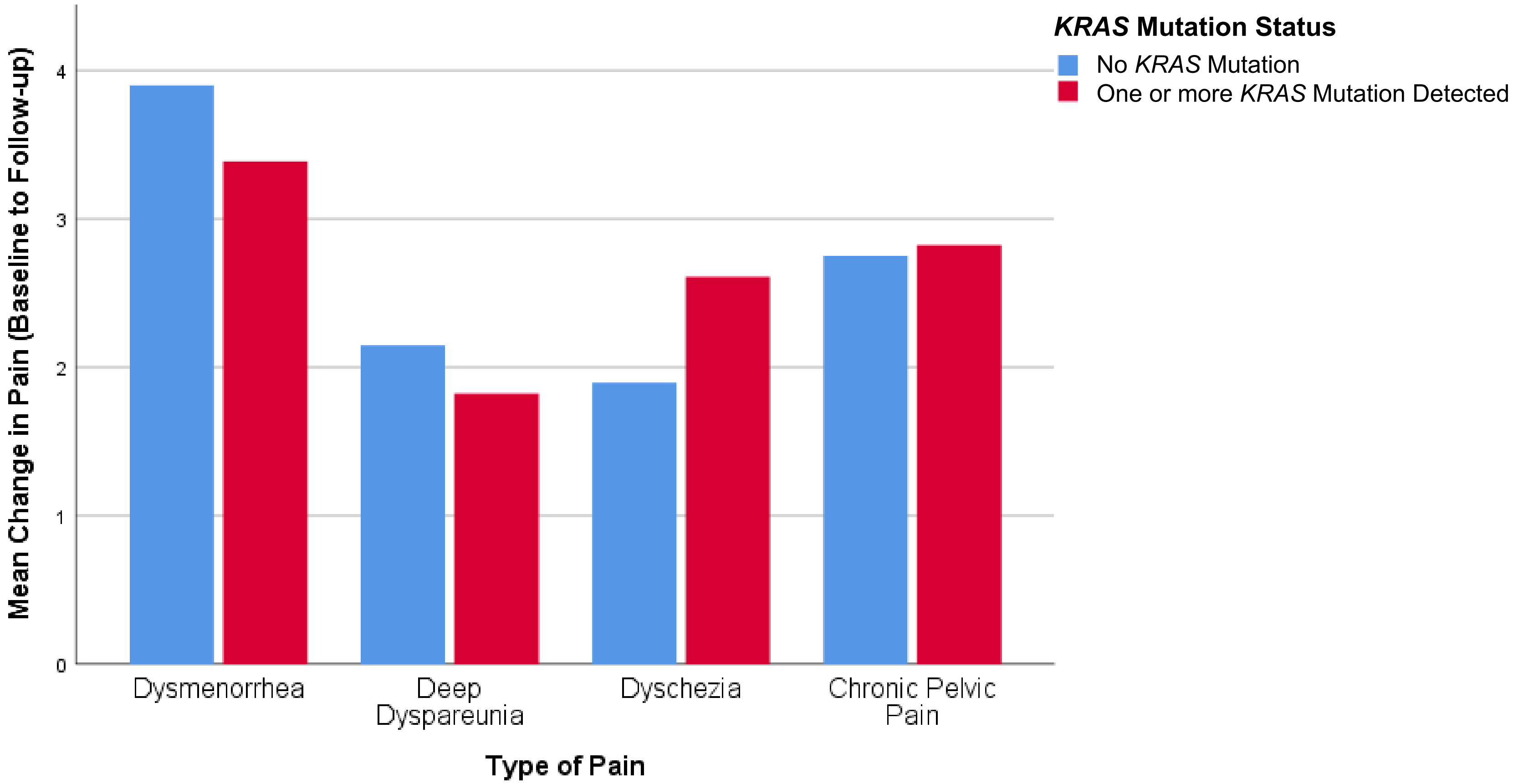

Figure 4. Change in pain scores between baseline and follow-up, based on mutation status.

The mean change in pain (baseline pain score minus follow-up pain score) is shown for four pain scores: dysmenorrhea, deep dyspareunia, dyschezia, and chronic pelvic pain. Pain scores were measured on a numeric rating scale from 0-10 (10=worst pain imaginable), as previously described [24]. Blue bars represent the mean change in pain for people with no *KRAS* mutations detected. Red bars represent the mean change in pain for people with *KRAS* mutation(s) detected. As shown, similar improvements in pain were seen after surgery in both groups.

Table S1. Comparison between manual macrodissection and laser capture microdissection.

Enrichment	No *KRAS* Mutation	*KRAS* Mutation	Chi Square	P Value
Laser capture microdissection<br>Manual macrodissection	22 (73·3%)<br>151 (65·1%)	8 (26·7%)<br>81 (34·9%)	0·81	0·42

Bivariate analysis identified no significance difference in detection of *KRAS* mutation between the samples enriched by manual needle macrodissection vs. laser capture microdissection. As well, seven cases of superficial peritoneal endometriosis, without *KRAS* mutation after manual macrodissection, were re-tested after laser capture microdissection which confirmed the absence of *KRAS* mutation.

Table S2. Cell line controls.

KRAS Assay	Cell Line
c.34G>T (p.G12C)	MIA PaCa-2 Cell Line
c.35G>A (p.G12D)	HEY Cell Line
c.34G>C (p.G12R)	PK-8 Cell Line
c.35G>T (p.G12V)	1:1 Mix of OvCar5 Cell Line with Wildtype DNA
c.35G>C (p.G12A)	H2009 Cell Line
c.34G>A (p.G12S)	1:1 Mix of A549 Cell Line and Wildtype DNA

Positive variants on multiplex ddPCR were confirmed with variant-specific ddPCR assays that were also used to establish variant allele frequency and sensitivity relative to cell line controls.

Table S3. Mean detection thresholds for the individual *KRAS* assays.

***KRAS* Variant**	**Mean Detection Threshold**	**Range**
c.34G>T (p.G12C)	0·10%	0·0040%-0·55%
c.35G>A (p.G12D)	0·76%	0·35%-1·5%
c.34G>C (p.G12R)	0·0070%	0·0020%-0·020%
c.35G>T (p.G12V)	0·013%	0%-0·27%
c.35G>C (p.G12A)	0·0028%	0%-0·0060%
c.34G>A (p.G12S)	0·81%	0·44%-1·2%

The mean detection thresholds (per assay/per run) and ranges for the individual, variant-specific *KRAS* mutation ddPCR assays used to confirm any suspected positive variants detected after multiplex ddPCR assay.

Table S4. Demographics.

The study sample is from a research data registry, with consented subjects recruited from a tertiary referral center for endometriosis and pelvic pain in the province of British Columbia, Canada. The Table below shows the diversity in various socioeconomic variables within the study sample.

It should be emphasized that the center has a minimum age cut-off of 18 years, and therefore adolescent patients with endometriosis will not be represented in this sample. There may also be issues with generalizability to community/primary care settings or to other provinces or countries. Moreover, the sample has proportionally fewer non-Caucasian ethnicities in comparison to census data from British Columbia.

Demographics of the study group:

**Variable[1]**	**Mean ± SD or # (%)**
Patient characteristics	
Age	34·4±6·6
Parity	
Parous	34 (27·9%)
Nulliparous	87 (71·3%)
Missing	1 (0·8%)
Ethnicity	
Caucasian only ethnicity	89 (73·0%)
Other ethnicity	28 (23·0%)
Mixed ethnicity	4 (3·3%)
Missing	1 (0·8%)
Sexual Orientation[2]	
Heterosexual	112 (91·8%)
Lesbian	2 (1·6%)
Bisexual	6 (4·9%)
Other	1 (0·8%)
Missing	1 (0·8%)
Employment status	
Working	93 (76·9%)
Not working	28 (23·1%)
Relationship status	
Single	26 (21·5%)
Dating	14 (11·6%)
Married	62 (51·2%)
Common-law	16 (13·2%)
Separated	1 (0·8%)
Divorced	2 (1·7%)
Education	
Graduated high school or earned GED	14 (11·6%)
Some college	33 (27·3%)
Graduated 2 years college	21 (17·4%)
Graduated 4 years college	21 (17·4%)
Post-grad degree	27 (22·3%)
Other	5 (4·1%)
Income (annual)	

<20K	13 (10·7%)
20-39K	20 (16·5%)
40-59K	20 (16·5%)
60-79K	16 (13·2%)
80-99K	19 (15·7%)
>100K	33 (27·3%)

[1]N = 121

[2]Sexual orientation was assessed but due to the small sample sizes in the non-heterosexual groups we could not analyze the data by sexual orientation. Gender identity was not assessed in our registry until 2018.

Table S5. Summary of mutation calls.

Case ID	Sample ID	Endo type	Anatomical Location	Enrich. method	Variant tested	Multi. cut-off VAF (%)	Multi. VAF (%)	Indiv. cut-off VAF (%)	Indiv. VAF (%)	Result	*KRAS* Variant
C_1	OMA27	OMA	Ovary (B)	Macro	G12C	0*	0*			Wildtype	
C_1	OMA27	OMA	Ovary (B)	Macro	G12D	0·043	0·0087			Wildtype	
C_1	OMA27	OMA	Ovary (B)	Macro	G12R	0*	0*			Wildtype	
C_1	OMA27	OMA	Ovary (B)	Macro	G12V	0·0020	0·0017			Wildtype	
C_1	OMA27	OMA	Ovary (B)	Macro	G12A	0*	0*			Wildtype	
C_1	OMA27	OMA	Ovary (B)	Macro	G12S	0·031	0·0090			Wildtype	
C_1	SUP72	SUP	Cul-de-sac	Macro	G12C	0*	0*			Wildtype	
C_1	SUP72	SUP	Cul-de-sac	Macro	G12D	0·019	0·0017			Wildtype	
C_1	SUP72	SUP	Cul-de-sac	Macro	G12R	0*	0*			Wildtype	
C_1	SUP72	SUP	Cul-de-sac	Macro	G12V	0·0016	0*			Wildtype	
C_1	SUP72	SUP	Cul-de-sac	Macro	G12A	0·0016	0*			Wildtype	
C_1	SUP72	SUP	Cul-de-sac	Macro	G12S	0·030	0*			Wildtype	
C_2	SUP46	SUP	Rectosigmoid junction	Macro	G12C	0*	0*			Wildtype	
C_2	SUP46	SUP	Rectosigmoid junction	Macro	G12D	0·027	0*			Wildtype	
C_2	SUP46	SUP	Rectosigmoid junction	Macro	G12R	0*	0*			Wildtype	
C_2	SUP46	SUP	Rectosigmoid junction	Macro	G12V	0*	0*			Wildtype	
C_2	SUP46	SUP	Rectosigmoid junction	Macro	G12A	0·0021	0*			Wildtype	
C_2	SUP46	SUP	Rectosigmoid junction	Macro	G12S	0·021	0*			Wildtype	
C_2	SUP80	SUP	Uterosacral (L)	Macro	G12C	0·0058	0*			Wildtype	
C_2	SUP80	SUP	Uterosacral (L)	Macro	G12D	0·084	0·0028			Wildtype	
C_2	SUP80	SUP	Uterosacral (L)	Macro	G12R	0*	0*			Wildtype	
C_2	SUP80	SUP	Uterosacral (L)	Macro	G12V	0·0036	0*			Wildtype	
C_2	SUP80	SUP	Uterosacral (L)	Macro	G12A	0·0022	0*			Wildtype	
C_2	SUP80	SUP	Uterosacral (L)	Macro	G12S	0·046	0·0043			Wildtype	

Case ID	Sample ID	Endo type	Anatomical Location	Enrich. method	Variant tested	Multi. cut-off VAF (%)	Multi. VAF (%)	Indiv. cut-off VAF (%)	Indiv. VAF (%)	Result	*KRAS* Variant
C_3	OMA28	OMA	Ovary (R)	Macro	G12C	0*	0*			Wildtype	
C_3	OMA28	OMA	Ovary (R)	Macro	G12D	0·019	0·0087			Wildtype	
C_3	OMA28	OMA	Ovary (R)	Macro	G12R	0*	0*			Wildtype	
C_3	OMA28	OMA	Ovary (R)	Macro	G12V	0·0016	0·0022	0·017	0·0017	Wildtype	
C_3	OMA28	OMA	Ovary (R)	Macro	G12A	0·0016	0*			Wildtype	
C_3	OMA28	OMA	Ovary (R)	Macro	G12S	0·030	0*			Wildtype	
C_4	SUP73	SUP	Pelvic sidewall (L)	Macro	G12C	0*	0*			Wildtype	
C_4	SUP73	SUP	Pelvic sidewall (L)	Macro	G12D	0·019	0*			Wildtype	
C_4	SUP73	SUP	Pelvic sidewall (L)	Macro	G12R	0*	0*			Wildtype	
C_4	SUP73	SUP	Pelvic sidewall (L)	Macro	G12V	0·0016	0*			Wildtype	
C_4	SUP73	SUP	Pelvic sidewall (L)	Macro	G12A	0·0016	0*			Wildtype	
C_4	SUP73	SUP	Pelvic sidewall (L)	Macro	G12S	0·030	0*			Wildtype	
C_4	SUP81	SUP	Cul-de-sac	Macro	G12C	0·0058	0*			Wildtype	
C_4	SUP81	SUP	Cul-de-sac	Macro	G12D	0·084	0*			Wildtype	
C_4	SUP81	SUP	Cul-de-sac	Macro	G12R	0*	0*			Wildtype	
C_4	SUP81	SUP	Cul-de-sac	Macro	G12V	0·0036	0·0028			Wildtype	
C_4	SUP81	SUP	Cul-de-sac	Macro	G12A	0·0022	0*			Wildtype	
C_4	SUP81	SUP	Cul-de-sac	Macro	G12S	0·046	0·0014			Wildtype	
C_5	SUP55	SUP	Appendix	Macro	G12C	0*	0*			Wildtype	
C_5	SUP55	SUP	Appendix	Macro	G12D	0·027	0·011			Wildtype	
C_5	SUP55	SUP	Appendix	Macro	G12R	0*	0*			Wildtype	
C_5	SUP55	SUP	Appendix	Macro	G12V	0*	0·0022	0·0065	0·0055	Wildtype	
C_5	SUP55	SUP	Appendix	Macro	G12A	0·0021	0*			Wildtype	
C_5	SUP55	SUP	Appendix	Macro	G12S	0·021	0·0070			Wildtype	
C_6	OMA6	OMA	Ovary (L)	Macro	G12C	0·0019	0*			Wildtype	
C_6	OMA6	OMA	Ovary (L)	Macro	G12D	0·022	0·031	0·64	0·26	Wildtype	
C_6	OMA6	OMA	Ovary (L)	Macro	G12R	0*	0·0031	0·0024	0·058	Mutant	G12R
C_6	OMA6	OMA	Ovary (L)	Macro	G12V	0·018	0·0031			Wildtype	

Case ID	Sample ID	Endo type	Anatomical Location	Enrich. method	Variant tested	Multi. cut-off VAF (%)	Multi. VAF (%)	Indiv. cut-off VAF (%)	Indiv. VAF (%)	Result	*KRAS* Variant
C_6	OMA6	OMA	Ovary (L)	Macro	G12A	0·0016	0·0031	0·0061	0·0026	Wildtype	
C_6	OMA6	OMA	Ovary (L)	Macro	G12S	0·025	0·022			Wildtype	
C_6	SUP1	SUP	Uterosacral (L)	Macro	G12C	0·0019	0*			Wildtype	
C_6	SUP1	SUP	Uterosacral (L)	Macro	G12D	0·022	0·0021			Wildtype	
C_6	SUP1	SUP	Uterosacral (L)	Macro	G12R	0*	0*			Wildtype	
C_6	SUP1	SUP	Uterosacral (L)	Macro	G12V	0·018	0·0021			Wildtype	
C_6	SUP1	SUP	Uterosacral (L)	Macro	G12A	0·0016	0*			Wildtype	
C_6	SUP1	SUP	Uterosacral (L)	Macro	G12S	0·025	0·0042			Wildtype	
C_6	SUP82	SUP	Cul-de-sac	Macro	G12C	0*	0*			Wildtype	
C_6	SUP82	SUP	Cul-de-sac	Macro	G12D	0·043	0·0091			Wildtype	
C_6	SUP82	SUP	Cul-de-sac	Macro	G12R	0*	0*			Wildtype	
C_6	SUP82	SUP	Cul-de-sac	Macro	G12V	0·0020	0*			Wildtype	
C_6	SUP82	SUP	Cul-de-sac	Macro	G12A	0*	0*			Wildtype	
C_6	SUP82	SUP	Cul-de-sac	Macro	G12S	0·031	0·011			Wildtype	
C_7	SUP56	SUP	Cul-de-sac (A)	Macro	G12C	0*	0*			Wildtype	
C_7	SUP56	SUP	Cul-de-sac (A)	Macro	G12D	0·027	0·026			Wildtype	
C_7	SUP56	SUP	Cul-de-sac (A)	Macro	G12R	0*	0*			Wildtype	
C_7	SUP56	SUP	Cul-de-sac (A)	Macro	G12V	0*	0·0024	0·0065	0·033	Mutant	G12V
C_7	SUP56	SUP	Cul-de-sac (A)	Macro	G12A	0·0021	0*			Wildtype	
C_7	SUP56	SUP	Cul-de-sac (A)	Macro	G12S	0·021	0·014			Wildtype	
C_7	SUP83	SUP	Uterosacral (L)	Macro	G12C	0*	0*			Wildtype	
C_7	SUP83	SUP	Uterosacral (L)	Macro	G12D	0·043	0*			Wildtype	
C_7	SUP83	SUP	Uterosacral (L)	Macro	G12R	0*	0*			Wildtype	
C_7	SUP83	SUP	Uterosacral (L)	Macro	G12V	0·0020	0*			Wildtype	
C_7	SUP83	SUP	Uterosacral (L)	Macro	G12A	0*	0*			Wildtype	
C_7	SUP83	SUP	Uterosacral (L)	Macro	G12S	0·031	0*			Wildtype	
C_8	SUP57	SUP	Uterosacral (L)	Macro	G12C	0*	0*			Wildtype	
C_8	SUP57	SUP	Uterosacral (L)	Macro	G12D	0·027	0·0021			Wildtype	

Case ID	Sample ID	Endo type	Anatomical Location	Enrich. method	Variant tested	Multi. cut-off VAF (%)	Multi. VAF (%)	Indiv. cut-off VAF (%)	Indiv. VAF (%)	Result	*KRAS* Variant
C_8	SUP57	SUP	Uterosacral (L)	Macro	G12R	0*	0*			Wildtype	
C_8	SUP57	SUP	Uterosacral (L)	Macro	G12V	0*	0*			Wildtype	
C_8	SUP57	SUP	Uterosacral (L)	Macro	G12A	0·0021	0·0021	0*	0*	Wildtype	
C_8	SUP57	SUP	Uterosacral (L)	Macro	G12S	0·021	0·0023			Wildtype	
C_9	SUP74	SUP	Cul-de-sac (L)	Macro	G12C	0·0058	0*			Wildtype	
C_9	SUP74	SUP	Cul-de-sac (L)	Macro	G12D	0·084	0·018			Wildtype	
C_9	SUP74	SUP	Cul-de-sac (L)	Macro	G12R	0*	0*			Wildtype	
C_9	SUP74	SUP	Cul-de-sac (L)	Macro	G12V	0·0036	0·0061	0·0053	0·030	Mutant	G12V
C_9	SUP74	SUP	Cul-de-sac (L)	Macro	G12A	0·0022	0*			Wildtype	
C_9	SUP74	SUP	Cul-de-sac (L)	Macro	G12S	0·046	0·0061			Wildtype	
C_9	SUP84	SUP	Pelvic sidewall (L)	Macro	G12C	0*	0*			Wildtype	
C_9	SUP84	SUP	Pelvic sidewall (L)	Macro	G12D	0·043	0·0029			Wildtype	
C_9	SUP84	SUP	Pelvic sidewall (L)	Macro	G12R	0*	0*			Wildtype	
C_9	SUP84	SUP	Pelvic sidewall (L)	Macro	G12V	0·0020	0*			Wildtype	
C_9	SUP84	SUP	Pelvic sidewall (L)	Macro	G12A	0*	0*			Wildtype	
C_9	SUP84	SUP	Pelvic sidewall (L)	Macro	G12S	0·031	0*			Wildtype	
C_10	DIE105	DIE	Ureter (L)	LCM	G12C	0·010	0·0088			Wildtype	
C_10	DIE105	DIE	Ureter (L)	LCM	G12D	0·043	0·040			Wildtype	
C_10	DIE105	DIE	Ureter (L)	LCM	G12R	0·0016	0*			Wildtype	
C_10	DIE105	DIE	Ureter (L)	LCM	G12V	0·010	0·018	0·010	0*	Wildtype	
C_10	DIE105	DIE	Ureter (L)	LCM	G12A	0·0046	0*			Wildtype	
C_10	DIE105	DIE	Ureter (L)	LCM	G12S	0·085	0·075	1·2	0·38	Wildtype	
C_10	SUP58	SUP	Ovary (NS)	Macro	G12C	0*	0*			Wildtype	
C_10	SUP58	SUP	Ovary (NS)	Macro	G12D	0·027	0·024			Wildtype	
C_10	SUP58	SUP	Ovary (NS)	Macro	G12R	0*	0*			Wildtype	
C_10	SUP58	SUP	Ovary (NS)	Macro	G12V	0*	0·0027	0·0065	0·0024	Wildtype	
C_10	SUP58	SUP	Ovary (NS)	Macro	G12A	0·0021	0*			Wildtype	
C_10	SUP58	SUP	Ovary (NS)	Macro	G12S	0·021	0·024	1·2	0·29	Wildtype	

**Case ID**	**Sample ID**	**Endo type**	**Anatomical Location**	**Enrich. method**	**Variant tested**	**Multi. cut-off VAF (%)**	**Multi. VAF (%)**	**Indiv. cut-off VAF (%)**	**Indiv. VAF (%)**	**Result**	***KRAS* Variant**
C_10	SUP85	SUP	Cul-de-sac	Macro	G12C	0*	0*			Wildtype	
C_10	SUP85	SUP	Cul-de-sac	Macro	G12D	0·043	0·069	1·5	0·39	Wildtype	
C_10	SUP85	SUP	Cul-de-sac	Macro	G12R	0*	0*			Wildtype	
C_10	SUP85	SUP	Cul-de-sac	Macro	G12V	0·0020	0*			Wildtype	
C_10	SUP85	SUP	Cul-de-sac	Macro	G12A	0*	0·0031	0·0022	0*	Wildtype	
C_10	SUP85	SUP	Cul-de-sac	Macro	G12S	0·031	0·0095			Wildtype	
C_11	OMA7	OMA	Ovary (L)	Macro	G12C	0·0019	0*	0·039	0·0057	Wildtype	
C_11	OMA7	OMA	Ovary (L)	Macro	G12D	0·022	0·011			Wildtype	
C_11	OMA7	OMA	Ovary (L)	Macro	G12R	0*	0·0022	0·0024	0·0026	Mutant	G12R
C_11	OMA7	OMA	Ovary (L)	Macro	G12V	0·018	0*	0·0016	0*	Wildtype	
C_11	OMA7	OMA	Ovary (L)	Macro	G12A	0·0016	0*			Wildtype	
C_11	OMA7	OMA	Ovary (L)	Macro	G12S	0·025	0·0022			Wildtype	
C_11	SUP2	SUP	Cul-de-sac (L)	Macro	G12C	0·0019	0*			Wildtype	
C_11	SUP2	SUP	Cul-de-sac (L)	Macro	G12D	0·022	0·012			Wildtype	
C_11	SUP2	SUP	Cul-de-sac (L)	Macro	G12R	0*	0*			Wildtype	
C_11	SUP2	SUP	Cul-de-sac (L)	Macro	G12V	0·018	0·12	0·0016	0*	Wildtype	
C_11	SUP2	SUP	Cul-de-sac (L)	Macro	G12A	0·0016	0·12	0·0061	0*	Wildtype	
C_11	SUP2	SUP	Cul-de-sac (L)	Macro	G12S	0·025	0*			Wildtype	
C_11	SUP86	SUP	Pelvic sidewall (R)	Macro	G12C	0*	0*			Wildtype	
C_11	SUP86	SUP	Pelvic sidewall (R)	Macro	G12D	0·043	0*			Wildtype	
C_11	SUP86	SUP	Pelvic sidewall (R)	Macro	G12R	0*	0*			Wildtype	
C_11	SUP86	SUP	Pelvic sidewall (R)	Macro	G12V	0·0020	0*			Wildtype	
C_11	SUP86	SUP	Pelvic sidewall (R)	Macro	G12A	0*	0*			Wildtype	
C_11	SUP86	SUP	Pelvic sidewall (R)	Macro	G12S	0·031	0*			Wildtype	
C_12	SUP3	SUP	Uterosacral (R)	Macro	G12C	0·0019	0*			Wildtype	
C_12	SUP3	SUP	Uterosacral (R)	Macro	G12D	0·022	0·43	0·64	5·3	Mutant	G12D
C_12	SUP3	SUP	Uterosacral (R)	Macro	G12R	0*	0*			Wildtype	
C_12	SUP3	SUP	Uterosacral (R)	Macro	G12V	0·018	0·0045			Wildtype	

Case ID	Sample ID	Endo type	Anatomical Location	Enrich. method	Variant tested	Multi. cut-off VAF (%)	Multi. VAF (%)	Indiv. cut-off VAF (%)	Indiv. VAF (%)	Result	*KRAS* Variant
C_12	SUP3	SUP	Uterosacral (R)	Macro	G12A	0·0016	0*			Wildtype	
C_12	SUP3	SUP	Uterosacral (R)	Macro	G12S	0·025	0·018			Wildtype	
C_12	SUP87	SUP	Ovary (B)	Macro	G12C	0·0058	0·063	0·072	1·2	Mutant	G12C
C_12	SUP87	SUP	Ovary (B)	Macro	G12D	0·084	0·011			Wildtype	
C_12	SUP87	SUP	Ovary (B)	Macro	G12R	0*	0·0021	0·0020	0·0015	Wildtype	
C_12	SUP87	SUP	Ovary (B)	Macro	G12V	0·0036	0·0021			Wildtype	
C_12	SUP87	SUP	Ovary (B)	Macro	G12A	0·0022	0*			Wildtype	
C_12	SUP87	SUP	Ovary (B)	Macro	G12S	0·046	0·013			Wildtype	
C_13	OMA8	OMA	Ovary (R)	Macro	G12C	0·0019	0*			Wildtype	
C_13	OMA8	OMA	Ovary (R)	Macro	G12D	0·022	0·026	0·64	0·25	Wildtype	
C_13	OMA8	OMA	Ovary (R)	Macro	G12R	0*	0*			Wildtype	
C_13	OMA8	OMA	Ovary (R)	Macro	G12V	0·018	0·096	0·0016	1·1	Mutant	G12V
C_13	OMA8	OMA	Ovary (R)	Macro	G12A	0·0016	0·026	0·0061	0·43	Mutant	G12A
C_13	OMA8	OMA	Ovary (R)	Macro	G12S	0·025	0·034	1·0	0·29	Wildtype	
C_13	SUP4	SUP	Uterosacral (L)	Macro	G12C	0·0019	0*			Wildtype	
C_13	SUP4	SUP	Uterosacral (L)	Macro	G12D	0·022	0·033	0·64	1·1	Mutant	G12D
C_13	SUP4	SUP	Uterosacral (L)	Macro	G12R	0*	0*			Wildtype	
C_13	SUP4	SUP	Uterosacral (L)	Macro	G12V	0·018	0·0025			Wildtype	
C_13	SUP4	SUP	Uterosacral (L)	Macro	G12A	0·0016	0*			Wildtype	
C_13	SUP4	SUP	Uterosacral (L)	Macro	G12S	0·025	0·0050			Wildtype	
C_13	SUP88	SUP	Cul-de-sac	Macro	G12C	0·0058	0*			Wildtype	
C_13	SUP88	SUP	Cul-de-sac	Macro	G12D	0·084	0·016			Wildtype	
C_13	SUP88	SUP	Cul-de-sac	Macro	G12R	0*	0*			Wildtype	
C_13	SUP88	SUP	Cul-de-sac	Macro	G12V	0·0036	0·0021			Wildtype	
C_13	SUP88	SUP	Cul-de-sac	Macro	G12A	0·0022	0·0021			Wildtype	
C_13	SUP88	SUP	Cul-de-sac	Macro	G12S	0·046	0·010			Wildtype	
C_14	OMA9	OMA	Ovary (L)	Macro	G12C	0·0019	0·0036	0·039	0·036	Wildtype	
C_14	OMA9	OMA	Ovary (L)	Macro	G12D	0·022	0·041	0·64	0·26	Wildtype	

Case ID	Sample ID	Endo type	Anatomical Location	Enrich. method	Variant tested	Multi. cut-off VAF (%)	Multi. VAF (%)	Indiv. cut-off VAF (%)	Indiv. VAF (%)	Result	*KRAS* Variant
C_14	OMA9	OMA	Ovary (L)	Macro	G12R	0*	0*			Wildtype	
C_14	OMA9	OMA	Ovary (L)	Macro	G12V	0·018	0·025	0·0016	0·026	Mutant	G12V
C_14	OMA9	OMA	Ovary (L)	Macro	G12A	0·0016	0·0051	0·0061	0·0060	Wildtype	
C_14	OMA9	OMA	Ovary (L)	Macro	G12S	0·025	0·010			Wildtype	
C_15	OMA10	OMA	Ovary (R)	Macro	G12C	0·0019	0*			Wildtype	
C_15	OMA10	OMA	Ovary (R)	Macro	G12D	0·022	0·36	0·64	0·98	Mutant	G12D
C_15	OMA10	OMA	Ovary (R)	Macro	G12R	0*	0*			Wildtype	
C_15	OMA10	OMA	Ovary (R)	Macro	G12V	0·018	3·1	0·0016	6·9	Mutant	G12V
C_15	OMA10	OMA	Ovary (R)	Macro	G12A	0·0016	0·020	0·0061	0·0059	Wildtype	
C_15	OMA10	OMA	Ovary (R)	Macro	G12S	0·025	0·30	1·0	0·72	Wildtype	
C_16	OMA11	OMA	Ovary (B)	Macro	G12C	0·0019	0*			Wildtype	
C_16	OMA11	OMA	Ovary (B)	Macro	G12D	0·022	0·015			Wildtype	
C_16	OMA11	OMA	Ovary (B)	Macro	G12R	0*	0*			Wildtype	
C_16	OMA11	OMA	Ovary (B)	Macro	G12V	0·018	0·0026	0·0016	0·013	Mutant	G12V
C_16	OMA11	OMA	Ovary (B)	Macro	G12A	0·0016	0*			Wildtype	
C_16	OMA11	OMA	Ovary (B)	Macro	G12S	0·025	0·023			Wildtype	
C_17	DIE80	DIE	Other ill-defined sites	Macro	G12C	0·0020	0*			Wildtype	
C_17	DIE80	DIE	Other ill-defined sites	Macro	G12D	0·0020	0*	0·77	0·17	Wildtype	
C_17	DIE80	DIE	Other ill-defined sites	Macro	G12R	0*	0*			Wildtype	
C_17	DIE80	DIE	Other ill-defined sites	Macro	G12V	0·0060	0*			Wildtype	
C_17	DIE80	DIE	Other ill-defined sites	Macro	G12A	0·0060	0*			Wildtype	
C_17	DIE80	DIE	Other ill-defined sites	Macro	G12S	0·0020	0·0014	0·67	0·35	Wildtype	
C_17	DIE81	DIE	Other ill-defined sites	Macro	G12C	0·017	0*			Wildtype	
C_17	DIE81	DIE	Other ill-defined sites	Macro	G12D	0·13	0*			Wildtype	
C_17	DIE81	DIE	Other ill-defined sites	Macro	G12R	0*	0*			Wildtype	
C_17	DIE81	DIE	Other ill-defined sites	Macro	G12V	0·0060	0*			Wildtype	

Case ID	Sample ID	Endo type	Anatomical Location	Enrich. method	Variant tested	Multi. cut-off VAF (%)	Multi. VAF (%)	Indiv. cut-off VAF (%)	Indiv. VAF (%)	Result	*KRAS* Variant
C_17	DIE81	DIE	Other ill-defined sites	Macro	G12A	0*	0*			Wildtype	
C_17	DIE81	DIE	Other ill-defined sites	Macro	G12S	0·045	0·13	0·73	0·76	Mutant	G12S
C_17	DIE82	DIE	Pelvic sidewall (R)	Macro	G12C	0·010	0*			Wildtype	
C_17	DIE82	DIE	Pelvic sidewall (R)	Macro	G12D	0·043	0·020			Wildtype	
C_17	DIE82	DIE	Pelvic sidewall (R)	Macro	G12R	0·0016	0*			Wildtype	
C_17	DIE82	DIE	Pelvic sidewall (R)	Macro	G12V	0·0100	0*			Wildtype	
C_17	DIE82	DIE	Pelvic sidewall (R)	Macro	G12A	0·0046	0*			Wildtype	
C_17	DIE82	DIE	Pelvic sidewall (R)	Macro	G12S	0·085	0·32	1·2	0·58	Wildtype	
C_17	SUP5	SUP	Uterosacral (L)	Macro	G12C	0·0019	0*			Wildtype	
C_17	SUP5	SUP	Uterosacral (L)	Macro	G12D	0·022	0·0079			Wildtype	
C_17	SUP5	SUP	Uterosacral (L)	Macro	G12R	0*	0*			Wildtype	
C_17	SUP5	SUP	Uterosacral (L)	Macro	G12V	0·018	0*	0·0016	0*	Wildtype	
C_17	SUP5	SUP	Uterosacral (L)	Macro	G12A	0·0016	0*			Wildtype	
C_17	SUP5	SUP	Uterosacral (L)	Macro	G12S	0·025	0·0059			Wildtype	
C_18	DIE85	DIE	Appendix	Macro	G12C	0·0020	0*			Wildtype	
C_18	DIE85	DIE	Appendix	Macro	G12D	0·056	0·010			Wildtype	
C_18	DIE85	DIE	Appendix	Macro	G12R	0*	0*			Wildtype	
C_18	DIE85	DIE	Appendix	Macro	G12V	0·0060	0·0035			Wildtype	
C_18	DIE85	DIE	Appendix	Macro	G12A	0·0060	0*			Wildtype	
C_18	DIE85	DIE	Appendix	Macro	G12S	0·0020	0·0035	0·73	0·42	Wildtype	
C_18	OMA12	OMA	Ovary (R)	Macro	G12C	0·0019	0*			Wildtype	
C_18	OMA12	OMA	Ovary (R)	Macro	G12D	0·022	0·0047			Wildtype	
C_18	OMA12	OMA	Ovary (R)	Macro	G12R	0*	0*			Wildtype	
C_18	OMA12	OMA	Ovary (R)	Macro	G12V	0·018	0*			Wildtype	
C_18	OMA12	OMA	Ovary (R)	Macro	G12A	0·0016	0*			Wildtype	
C_18	OMA12	OMA	Ovary (R)	Macro	G12S	0·025	0·0024			Wildtype	
C_18	SUP6	SUP	Uterosacral (L)	Macro	G12C	0·0019	0*			Wildtype	
C_18	SUP6	SUP	Uterosacral (L)	Macro	G12D	0·022	0·0079			Wildtype	

Case ID	Sample ID	Endo type	Anatomical Location	Enrich. method	Variant tested	Multi. cut-off VAF (%)	Multi. VAF (%)	Indiv. cut-off VAF (%)	Indiv. VAF (%)	Result	*KRAS* Variant
C_18	SUP6	SUP	Uterosacral (L)	Macro	G12R	0*	0*			Wildtype	
C_18	SUP6	SUP	Uterosacral (L)	Macro	G12V	0·018	0·0020			Wildtype	
C_18	SUP6	SUP	Uterosacral (L)	Macro	G12A	0·0016	0*			Wildtype	
C_18	SUP6	SUP	Uterosacral (L)	Macro	G12S	0·025	0·0079			Wildtype	
C_19	DIE96	DIE	Cul-de-sac	LCM	G12C	0·010	0*			Wildtype	
C_19	DIE96	DIE	Cul-de-sac	LCM	G12D	0·043	0·0068			Wildtype	
C_19	DIE96	DIE	Cul-de-sac	LCM	G12R	0·0016	0*			Wildtype	
C_19	DIE96	DIE	Cul-de-sac	LCM	G12V	0·010	0·0023			Wildtype	
C_19	DIE96	DIE	Cul-de-sac	LCM	G12A	0·0046	0*			Wildtype	
C_19	DIE96	DIE	Cul-de-sac	LCM	G12S	0·085	0·0068			Wildtype	
C_20	DIE83	DIE	Uterosacral (L)	Macro	G12C	0·017	0*	0·030	0*	Wildtype	
C_20	DIE83	DIE	Uterosacral (L)	Macro	G12D	0·13	0·13	1·0	0·19	Wildtype	
C_20	DIE83	DIE	Uterosacral (L)	Macro	G12R	0*	0*	0·0020	0*	Wildtype	
C_20	DIE83	DIE	Uterosacral (L)	Macro	G12V	0·013	0*	0·010	0*	Wildtype	
C_20	DIE83	DIE	Uterosacral (L)	Macro	G12A	0*	0*	0·0049	0*	Wildtype	
C_20	DIE83	DIE	Uterosacral (L)	Macro	G12S	0·045	0·10	0·73	0·26	Wildtype	
C_20	DIE84	DIE	Cul-de-sac	Macro	G12C	0·013	0*			Wildtype	
C_20	DIE84	DIE	Cul-de-sac	Macro	G12D	0·0020	0*			Wildtype	
C_20	DIE84	DIE	Cul-de-sac	Macro	G12R	0*	0*			Wildtype	
C_20	DIE84	DIE	Cul-de-sac	Macro	G12V	0·0060	0·050	0·019	0·74	Mutant	G12V
C_20	DIE84	DIE	Cul-de-sac	Macro	G12A	0·0060	0*			Wildtype	
C_20	DIE84	DIE	Cul-de-sac	Macro	G12S	0·0020	0·010	0·73	0·48	Wildtype	
C_20	SUP7	SUP	Pelvic sidewall (L)	Macro	G12C	0·0019	0*			Wildtype	
C_20	SUP7	SUP	Pelvic sidewall (L)	Macro	G12D	0·022	0*			Wildtype	
C_20	SUP7	SUP	Pelvic sidewall (L)	Macro	G12R	0*	0*			Wildtype	
C_20	SUP7	SUP	Pelvic sidewall (L)	Macro	G12V	0·018	0*			Wildtype	
C_20	SUP7	SUP	Pelvic sidewall (L)	Macro	G12A	0·0016	0*			Wildtype	
C_20	SUP7	SUP	Pelvic sidewall (L)	Macro	G12S	0·025	0·016			Wildtype	

Case ID	Sample ID	Endo type	Anatomical Location	Enrich. method	Variant tested	Multi. cut-off VAF (%)	Multi. VAF (%)	Indiv. cut-off VAF (%)	Indiv. VAF (%)	Result	*KRAS* Variant
C_21	DIE101	DIE	Cul-de-sac	LCM	G12C	0·010	0*			Wildtype	
C_21	DIE101	DIE	Cul-de-sac	LCM	G12D	0·043	0·010			Wildtype	
C_21	DIE101	DIE	Cul-de-sac	LCM	G12R	0·0016	0*			Wildtype	
C_21	DIE101	DIE	Cul-de-sac	LCM	G12V	0·010	0·0035	0·11	0*	Wildtype	
C_21	DIE101	DIE	Cul-de-sac	LCM	G12A	0·0046	0·0035			Wildtype	
C_21	DIE101	DIE	Cul-de-sac	LCM	G12S	0·085	0·028			Wildtype	
C_21	OMA13	OMA	Ovary (R)	Macro	G12C	0·0019	0·0036	0·039	0·0025	Wildtype	
C_21	OMA13	OMA	Ovary (R)	Macro	G12D	0·022	0·082	0·64	0·79	Mutant	G12D
C_21	OMA13	OMA	Ovary (R)	Macro	G12R	0*	0*			Wildtype	
C_21	OMA13	OMA	Ovary (R)	Macro	G12V	0·018	0·022	0·0016	0*	Wildtype	
C_21	OMA13	OMA	Ovary (R)	Macro	G12A	0·0016	0·0071	0·0061	0*	Wildtype	
C_21	OMA13	OMA	Ovary (R)	Macro	G12S	0·025	0·025	1··0	0·26	Wildtype	
C_22	DIE6	DIE	Cul-de-sac (L)	LCM	G12C	0·0020	0·31	0·55	0·13	Wildtype	
C_22	DIE6	DIE	Cul-de-sac (L)	LCM	G12D	0·0020	0·64	0·77	4·2	Mutant	G12D
C_22	DIE6	DIE	Cul-de-sac (L)	LCM	G12R	0*	0*			Wildtype	
C_22	DIE6	DIE	Cul-de-sac (L)	LCM	G12V	0·0060	0*			Wildtype	
C_22	DIE6	DIE	Cul-de-sac (L)	LCM	G12A	0·0060	0*			Wildtype	
C_22	DIE6	DIE	Cul-de-sac (L)	LCM	G12S	0·0020	0·047	0·67	0·35	Wildtype	
C_22	DIE7	DIE	Cul-de-sac (R)	Macro	G12C	0·0020	0·055	0·030	0*	Wildtype	
C_22	DIE7	DIE	Cul-de-sac (R)	Macro	G12D	0·0020	0·055	0·77	1·7	Mutant	G12D
C_22	DIE7	DIE	Cul-de-sac (R)	Macro	G12R	0*	0*			Wildtype	
C_22	DIE7	DIE	Cul-de-sac (R)	Macro	G12V	0·0060	0*			Wildtype	
C_22	DIE7	DIE	Cul-de-sac (R)	Macro	G12A	0·0060	0·0021			Wildtype	
C_22	DIE7	DIE	Cul-de-sac (R)	Macro	G12S	0·0020	0·0080	0·73	0·14	Wildtype	
C_22	DIE8	DIE	Pelvic sidewall (R)	Macro	G12C	0·017	0*			Wildtype	
C_22	DIE8	DIE	Pelvic sidewall (R)	Macro	G12D	0·13	0·038	0·77	0·67	Wildtype	
C_22	DIE8	DIE	Pelvic sidewall (R)	Macro	G12R	0*	0*			Wildtype	
C_22	DIE8	DIE	Pelvic sidewall (R)	Macro	G12V	0·0060	0·0037			Wildtype	

Case ID	Sample ID	Endo type	Anatomical Location	Enrich. method	Variant tested	Multi. cut-off VAF (%)	Multi. VAF (%)	Indiv. cut-off VAF (%)	Indiv. VAF (%)	Result	*KRAS* Variant
C_22	DIE8	DIE	Pelvic sidewall (R)	Macro	G12A	0*	0·072	0·0015	0·60	Mutant	G12A
C_22	DIE8	DIE	Pelvic sidewall (R)	Macro	G12S	0·045	0·062	0·67	0·39	Wildtype	
C_22	DIE9	DIE	Rectum	LCM	G12C	0·0020	0*	0·55	0·043	Wildtype	
C_22	DIE9	DIE	Rectum	LCM	G12D	0·0020	0·015	0·77	0·25	Wildtype	
C_22	DIE9	DIE	Rectum	LCM	G12R	0*	0*			Wildtype	
C_22	DIE9	DIE	Rectum	LCM	G12V	0·0060	0*			Wildtype	
C_22	DIE9	DIE	Rectum	LCM	G12A	0·0060	0*			Wildtype	
C_22	DIE9	DIE	Rectum	LCM	G12S	0·0020	0·027	0·67	0·24	Wildtype	
C_22	DIE10	DIE	Cul-de-sac (A)	Macro	G12C	0·010	0*			Wildtype	
C_22	DIE10	DIE	Cul-de-sac (A)	Macro	G12D	0·043	0·049	0·74	0·24	Wildtype	
C_22	DIE10	DIE	Cul-de-sac (A)	Macro	G12R	0·0016	0*			Wildtype	
C_22	DIE10	DIE	Cul-de-sac (A)	Macro	G12V	0·010	0·0054			Wildtype	
C_22	DIE10	DIE	Cul-de-sac (A)	Macro	G12A	0·0046	0*			Wildtype	
C_22	DIE10	DIE	Cul-de-sac (A)	Macro	G12S	0·085	0·032	1·2	0·15	Wildtype	
C_23	SUP8	SUP	Cul-de-sac	Macro	G12C	0·0041	0*			Wildtype	
C_23	SUP8	SUP	Cul-de-sac	Macro	G12D	0·018	0·023	0·73	0·22	Wildtype	
C_23	SUP8	SUP	Cul-de-sac	Macro	G12R	0·0014	0*			Wildtype	
C_23	SUP8	SUP	Cul-de-sac	Macro	G12V	0·00070	0*			Wildtype	
C_23	SUP8	SUP	Cul-de-sac	Macro	G12A	0*	0*			Wildtype	
C_23	SUP8	SUP	Cul-de-sac	Macro	G12S	0·033	0·027			Wildtype	
C_24	DIE4	DIE	Uterosacral (R)	LCM	G12C	0·010	0·0097			Wildtype	
C_24	DIE4	DIE	Uterosacral (R)	LCM	G12D	0·043	0·029			Wildtype	
C_24	DIE4	DIE	Uterosacral (R)	LCM	G12R	0·0016	0*			Wildtype	
C_24	DIE4	DIE	Uterosacral (R)	LCM	G12V	0·010	0*			Wildtype	
C_24	DIE4	DIE	Uterosacral (R)	LCM	G12A	0·0046	0*			Wildtype	
C_24	DIE4	DIE	Uterosacral (R)	LCM	G12S	0·085	0·20	0·67	0·19	Wildtype	
C_24	DIE5	DIE	Vagina	Macro	G12C	0·0020	0·0016			Wildtype	
C_24	DIE5	DIE	Vagina	Macro	G12D	0·0020	0·0018			Wildtype	

Case ID	Sample ID	Endo type	Anatomical Location	Enrich. method	Variant tested	Multi. cut-off VAF (%)	Multi. VAF (%)	Indiv. cut-off VAF (%)	Indiv. VAF (%)	Result	*KRAS* Variant
C_24	DIE5	DIE	Vagina	Macro	G12R	0*	0*			Wildtype	
C_24	DIE5	DIE	Vagina	Macro	G12V	0·0060	0*			Wildtype	
C_24	DIE5	DIE	Vagina	Macro	G12A	0·0060	0*			Wildtype	
C_24	DIE5	DIE	Vagina	Macro	G12S	0·0020	0·0033	0·73	0·18	Wildtype	
C_25	OMA2	OMA	Ovary (L)	Macro	G12C	0·010	0*			Wildtype	
C_25	OMA2	OMA	Ovary (L)	Macro	G12D	0·043	0·030	0·74	0·23	Wildtype	
C_25	OMA2	OMA	Ovary (L)	Macro	G12R	0·0016	0*			Wildtype	
C_25	OMA2	OMA	Ovary (L)	Macro	G12V	0·010	0*			Wildtype	
C_25	OMA2	OMA	Ovary (L)	Macro	G12A	0·0046	0*			Wildtype	
C_25	OMA2	OMA	Ovary (L)	Macro	G12S	0·085	0·040	1·2	0·38	Wildtype	
C_25	SUP9	SUP	Cul-de-sac	Macro	G12C	0·0041	0*			Wildtype	
C_25	SUP9	SUP	Cul-de-sac	Macro	G12D	0·018	0*			Wildtype	
C_25	SUP9	SUP	Cul-de-sac	Macro	G12R	0·0014	0*			Wildtype	
C_25	SUP9	SUP	Cul-de-sac	Macro	G12V	failed - not enough droplets	failed - not enough droplets	0·0060	1·7	Mutant	G12V
C_25	SUP9	SUP	Cul-de-sac	Macro	G12A	0*	0*			Wildtype	
C_25	SUP9	SUP	Cul-de-sac	Macro	G12S	failed - not enough droplets	failed - not enough droplets	0·44	0·91	Mutant	G12S
C_26	DIE72	DIE	Rectum	Macro	G12C	0·013	0*			Wildtype	
C_26	DIE72	DIE	Rectum	Macro	G12D	0·0020	0·0079	0·77	0·49	Wildtype	
C_26	DIE72	DIE	Rectum	Macro	G12R	0*	0*			Wildtype	
C_26	DIE72	DIE	Rectum	Macro	G12V	0·0060	0*			Wildtype	
C_26	DIE72	DIE	Rectum	Macro	G12A	0·0060	0*			Wildtype	
C_26	DIE72	DIE	Rectum	Macro	G12S	0·0020	0·031	0·73	0·17	Wildtype	
C_26	DIE73	DIE	Rectum	Macro	G12C	0·013	0*			Wildtype	
C_26	DIE73	DIE	Rectum	Macro	G12D	0·0020	0·068	0·77	0·36	Wildtype	
C_26	DIE73	DIE	Rectum	Macro	G12R	0*	0*			Wildtype	

Case ID	Sample ID	Endo type	Anatomical Location	Enrich. method	Variant tested	Multi. cut-off VAF (%)	Multi. VAF (%)	Indiv. cut-off VAF (%)	Indiv. VAF (%)	Result	*KRAS* Variant
C_26	DIE73	DIE	Rectum	Macro	G12V	0·0060	0*			Wildtype	
C_26	DIE73	DIE	Rectum	Macro	G12A	0·0060	0*			Wildtype	
C_26	DIE73	DIE	Rectum	Macro	G12S	0·0020	0·14	0·67	0·50	Wildtype	
C_26	DIE74	DIE	Cul-de-sac (L)	LCM	G12C	0·010	0*			Wildtype	
C_26	DIE74	DIE	Cul-de-sac (L)	LCM	G12D	0·043	0·065	0·74	0·31	Wildtype	
C_26	DIE74	DIE	Cul-de-sac (L)	LCM	G12R	0·0016	0*			Wildtype	
C_26	DIE74	DIE	Cul-de-sac (L)	LCM	G12V	0·010	0·0038			Wildtype	
C_26	DIE74	DIE	Cul-de-sac (L)	LCM	G12A	0·0046	0*			Wildtype	
C_26	DIE74	DIE	Cul-de-sac (L)	LCM	G12S	0·085	0·015			Wildtype	
C_26	DIE75	DIE	Cul-de-sac (L)	Macro	G12C	0·017	0*			Wildtype	
C_26	DIE75	DIE	Cul-de-sac (L)	Macro	G12D	0·13	0*			Wildtype	
C_26	DIE75	DIE	Cul-de-sac (L)	Macro	G12R	0*	0*			Wildtype	
C_26	DIE75	DIE	Cul-de-sac (L)	Macro	G12V	0·013	0·024	0·010	0*	Wildtype	
C_26	DIE75	DIE	Cul-de-sac (L)	Macro	G12A	0*	0*			Wildtype	
C_26	DIE75	DIE	Cul-de-sac (L)	Macro	G12S	0·045	0·097	0·67	0·16	Wildtype	
C_26	DIE76	DIE	Cul-de-sac (L)	Macro	G12C	0·017	0*			Wildtype	
C_26	DIE76	DIE	Cul-de-sac (L)	Macro	G12D	0·13	0·12	0·77	0·12	Wildtype	
C_26	DIE76	DIE	Cul-de-sac (L)	Macro	G12R	0*	0*			Wildtype	
C_26	DIE76	DIE	Cul-de-sac (L)	Macro	G12V	0·013	0*			Wildtype	
C_26	DIE76	DIE	Cul-de-sac (L)	Macro	G12A	0*	0*			Wildtype	
C_26	DIE76	DIE	Cul-de-sac (L)	Macro	G12S	0·045	0·17	0·67	0·33	Wildtype	
C_26	DIE77	DIE	Pelvic sidewall (R)	LCM	G12C	0·010	0*			Wildtype	
C_26	DIE77	DIE	Pelvic sidewall (R)	LCM	G12D	0·043	0·018			Wildtype	
C_26	DIE77	DIE	Pelvic sidewall (R)	LCM	G12R	0·0016	0*			Wildtype	
C_26	DIE77	DIE	Pelvic sidewall (R)	LCM	G12V	0·010	0*			Wildtype	
C_26	DIE77	DIE	Pelvic sidewall (R)	LCM	G12A	0·0046	0*			Wildtype	
C_26	DIE77	DIE	Pelvic sidewall (R)	LCM	G12S	0·0084	0·16	0·53	0·50	Wildtype	
C_26	DIE78	DIE	Pelvic sidewall (L)	LCM	G12C	0·010	0*			Wildtype	

Case ID	Sample ID	Endo type	Anatomical Location	Enrich. method	Variant tested	Multi. cut-off VAF (%)	Multi. VAF (%)	Indiv. cut-off VAF (%)	Indiv. VAF (%)	Result	*KRAS* Variant
C_26	DIE78	DIE	Pelvic sidewall (L)	LCM	G12D	0·043	0·012			Wildtype	
C_26	DIE78	DIE	Pelvic sidewall (L)	LCM	G12R	0·0016	0·0077	0·020	0·016	Wildtype	
C_26	DIE78	DIE	Pelvic sidewall (L)	LCM	G12V	0·010	0·0039			Wildtype	
C_26	DIE78	DIE	Pelvic sidewall (L)	LCM	G12A	0·0046	0*			Wildtype	
C_26	DIE78	DIE	Pelvic sidewall (L)	LCM	G12S	0·085	0·066	1·2	0·38	Wildtype	
C_27	SUP75	SUP	Pelvic sidewall (R)	Macro	G12C	0*	0*			Wildtype	
C_27	SUP75	SUP	Pelvic sidewall (R)	Macro	G12D	0·043	0*			Wildtype	
C_27	SUP75	SUP	Pelvic sidewall (R)	Macro	G12R	0*	0*			Wildtype	
C_27	SUP75	SUP	Pelvic sidewall (R)	Macro	G12V	0·0020	0*			Wildtype	
C_27	SUP75	SUP	Pelvic sidewall (R)	Macro	G12A	0*	0·0015	0·0022	0·0013	Wildtype	
C_27	SUP75	SUP	Pelvic sidewall (R)	Macro	G12S	0·031	0·0047			Wildtype	
C_27	SUP89	SUP	Pelvic sidewall (R)	Macro	G12C	0*	0*			Wildtype	
C_27	SUP89	SUP	Pelvic sidewall (R)	Macro	G12D	0·043	0·040	1·5	0·33	Wildtype	
C_27	SUP89	SUP	Pelvic sidewall (R)	Macro	G12R	0*	0*			Wildtype	
C_27	SUP89	SUP	Pelvic sidewall (R)	Macro	G12V	0·0020	0·0091	0·0039	0·0074	Mutant	G12V
C_27	SUP89	SUP	Pelvic sidewall (R)	Macro	G12A	0*	0*			Wildtype	
C_27	SUP89	SUP	Pelvic sidewall (R)	Macro	G12S	0·031	0·040	0·78	0·42	Wildtype	
C_28	DIE99	DIE	Uterosacral (L)	Macro	G12C	0·010	0*			Wildtype	
C_28	DIE99	DIE	Uterosacral (L)	Macro	G12D	0·043	0·13	0·74	0·13	Wildtype	
C_28	DIE99	DIE	Uterosacral (L)	Macro	G12R	0·0016	0*			Wildtype	
C_28	DIE99	DIE	Uterosacral (L)	Macro	G12V	0·010	0·015	0·11	0·12	Mutant	G12V
C_28	DIE99	DIE	Uterosacral (L)	Macro	G12A	0·0046	0*			Wildtype	
C_28	DIE99	DIE	Uterosacral (L)	Macro	G12S	0·085	0·044			Wildtype	
C_28	DIE100	DIE	Uterosacral (R)	LCM	G12C	0·010	0*			Wildtype	
C_28	DIE100	DIE	Uterosacral (R)	LCM	G12D	0·042	0·27	0·69	0·92	Mutant	G12D
C_28	DIE100	DIE	Uterosacral (R)	LCM	G12R	0·0016	0*			Wildtype	
C_28	DIE100	DIE	Uterosacral (R)	LCM	G12V	0·010	0*			Wildtype	
C_28	DIE100	DIE	Uterosacral (R)	LCM	G12A	0·0046	0*			Wildtype	

Case ID	Sample ID	Endo type	Anatomical Location	Enrich. method	Variant tested	Multi. cut-off VAF (%)	Multi. VAF (%)	Indiv. cut-off VAF (%)	Indiv. VAF (%)	Result	*KRAS* Variant
C_28	DIE100	DIE	Uterosacral (R)	LCM	G12S	0·085	0·078			Wildtype	
C_28	SUP47	SUP	Cul-de-sac	Macro	G12C	0*	0*			Wildtype	
C_28	SUP47	SUP	Cul-de-sac	Macro	G12D	0·027	0·0049			Wildtype	
C_28	SUP47	SUP	Cul-de-sac	Macro	G12R	0*	0*			Wildtype	
C_28	SUP47	SUP	Cul-de-sac	Macro	G12V	0*	0*			Wildtype	
C_28	SUP47	SUP	Cul-de-sac	Macro	G12A	0·0021	0·0024	0*	0·026	Mutant	G12A
C_28	SUP47	SUP	Cul-de-sac	Macro	G12S	0·021	0·0050			Wildtype	
C_29	OMA29	OMA	Ovary (R)	Macro	G12C	0*	0*			Wildtype	
C_29	OMA29	OMA	Ovary (R)	Macro	G12D	0·043	0*			Wildtype	
C_29	OMA29	OMA	Ovary (R)	Macro	G12R	0*	0*			Wildtype	
C_29	OMA29	OMA	Ovary (R)	Macro	G12V	0·0020	0·018			Wildtype	
C_29	OMA29	OMA	Ovary (R)	Macro	G12A	0*	0·0018	0·0022	0*	Wildtype	
C_29	OMA29	OMA	Ovary (R)	Macro	G12S	0·031	0·0055			Wildtype	
C_30	SUP10	SUP	Uterosacral (R)	Macro	G12C	0·0041	0*			Wildtype	
C_30	SUP10	SUP	Uterosacral (R)	Macro	G12D	0·018	0·0056			Wildtype	
C_30	SUP10	SUP	Uterosacral (R)	Macro	G12R	0·0014	0*			Wildtype	
C_30	SUP10	SUP	Uterosacral (R)	Macro	G12V	0·00070	0*			Wildtype	
C_30	SUP10	SUP	Uterosacral (R)	Macro	G12A	0*	0*			Wildtype	
C_30	SUP10	SUP	Uterosacral (R)	Macro	G12S	0·033	0·011			Wildtype	
C_30	SUP90	SUP	Pelvic sidewall (R)	Macro	G12C	0*	0*			Wildtype	
C_30	SUP90	SUP	Pelvic sidewall (R)	Macro	G12D	0·043	0·063	1·5	0·36	Wildtype	
C_30	SUP90	SUP	Pelvic sidewall (R)	Macro	G12R	0*	0*			Wildtype	
C_30	SUP90	SUP	Pelvic sidewall (R)	Macro	G12V	0·0020	0·0085	0·0039	0·0030	Wildtype	
C_30	SUP90	SUP	Pelvic sidewall (R)	Macro	G12A	0*	0·0028	0·0022	0*	Wildtype	
C_30	SUP90	SUP	Pelvic sidewall (R)	Macro	G12S	0·031	0·0057			Wildtype	
C_31	OMA14	OMA	Ovary (R)	Macro	G12C	0·0019	0·0062	0·039	0·0041	Wildtype	
C_31	OMA14	OMA	Ovary (R)	Macro	G12D	0·022	0·78	0·64	2·8	Mutant	G12D
C_31	OMA14	OMA	Ovary (R)	Macro	G12R	0*	0*			Wildtype	

Case ID	Sample ID	Endo type	Anatomical Location	Enrich. method	Variant tested	Multi. cut-off VAF (%)	Multi. VAF (%)	Indiv. cut-off VAF (%)	Indiv. VAF (%)	Result	*KRAS* Variant
C_31	OMA14	OMA	Ovary (R)	Macro	G12V	0·018	0·025	0·0016	0·0045	Mutant	G12V
C_31	OMA14	OMA	Ovary (R)	Macro	G12A	0·0016	0·0062	0·0061	0·0050	Wildtype	
C_31	OMA14	OMA	Ovary (R)	Macro	G12S	0·025	0·056	1·0	0·25	Wildtype	
C_31	OMA42	OMA	Ovary (L)	Macro	G12C	0·0058	0*			Wildtype	
C_31	OMA42	OMA	Ovary (L)	Macro	G12D	0·084	0·40	0·98	3·7	Mutant	G12D
C_31	OMA42	OMA	Ovary (L)	Macro	G12R	0*	0·0058	0·0020	0·070	Mutant	G12R
C_31	OMA42	OMA	Ovary (L)	Macro	G12V	0·0036	0·061	0·0053	0·59	Mutant	G12V
C_31	OMA42	OMA	Ovary (L)	Macro	G12A	0·0022	0*			Wildtype	
C_31	OMA42	OMA	Ovary (L)	Macro	G12S	0·046	0·020			Wildtype	
C_31	SUP11	SUP	Pelvic sidewall (L)	Macro	G12C	0·0041	0*			Wildtype	
C_31	SUP11	SUP	Pelvic sidewall (L)	Macro	G12D	0·018	0·17	0·73	0·56	Wildtype	
C_31	SUP11	SUP	Pelvic sidewall (L)	Macro	G12R	0·0014	0*			Wildtype	
C_31	SUP11	SUP	Pelvic sidewall (L)	Macro	G12V	0·00070	0·011	0·0060	0*	Wildtype	
C_31	SUP11	SUP	Pelvic sidewall (L)	Macro	G12A	0*	0*			Wildtype	
C_31	SUP11	SUP	Pelvic sidewall (L)	Macro	G12S	0·033	0·18	0·44	0·49	Mutant	G12S
C_32	DIE88	DIE	Cul-de-sac	Macro	G12C	0·010	0*			Wildtype	
C_32	DIE88	DIE	Cul-de-sac	Macro	G12D	0·043	0·029	0·74	0·31	Wildtype	
C_32	DIE88	DIE	Cul-de-sac	Macro	G12R	0·0016	0·0058	0·020	0·016	Wildtype	
C_32	DIE88	DIE	Cul-de-sac	Macro	G12V	0·010	0*			Wildtype	
C_32	DIE88	DIE	Cul-de-sac	Macro	G12A	0·0046	0*			Wildtype	
C_32	DIE88	DIE	Cul-de-sac	Macro	G12S	0·085	0·037	1·2	0·39	Wildtype	
C_32	OMA30	OMA	Ovary (L)	Macro	G12C	0*	0·0018	0·030	0·025	Wildtype	
C_32	OMA30	OMA	Ovary (L)	Macro	G12D	0·043	0·011			Wildtype	
C_32	OMA30	OMA	Ovary (L)	Macro	G12R	0*	0*			Wildtype	
C_32	OMA30	OMA	Ovary (L)	Macro	G12V	0·0020	0·0068	0·0039	0·048	Mutant	G12V
C_32	OMA30	OMA	Ovary (L)	Macro	G12A	0*	0*			Wildtype	
C_32	OMA30	OMA	Ovary (L)	Macro	G12S	0·031	0·0089			Wildtype	
C_32	SUP77	SUP	Cul-de-sac	Macro	G12C	0*	0*			Wildtype	

Case ID	Sample ID	Endo type	Anatomical Location	Enrich. method	Variant tested	Multi. cut-off VAF (%)	Multi. VAF (%)	Indiv. cut-off VAF (%)	Indiv. VAF (%)	Result	*KRAS* Variant
C_32	SUP77	SUP	Cul-de-sac	Macro	G12D	0·043	0*			Wildtype	
C_32	SUP77	SUP	Cul-de-sac	Macro	G12R	0*	0*			Wildtype	
C_32	SUP77	SUP	Cul-de-sac	Macro	G12V	0·0020	0*			Wildtype	
C_32	SUP77	SUP	Cul-de-sac	Macro	G12A	0*	0*			Wildtype	
C_32	SUP77	SUP	Cul-de-sac	Macro	G12S	0·031	0*			Wildtype	
C_33	DIE93	DIE	Uterosacral (R)	LCM	G12C	0·010	0·0078			Wildtype	
C_33	DIE93	DIE	Uterosacral (R)	LCM	G12D	0·043	0·047	0·69	0·39	Wildtype	
C_33	DIE93	DIE	Uterosacral (R)	LCM	G12R	0·0016	0*			Wildtype	
C_33	DIE93	DIE	Uterosacral (R)	LCM	G12V	0·010	0·0078			Wildtype	
C_33	DIE93	DIE	Uterosacral (R)	LCM	G12A	0·0046	0*			Wildtype	
C_33	DIE93	DIE	Uterosacral (R)	LCM	G12S	0·085	0·10	0·53	0·35	Wildtype	
C_33	DIE94	DIE	Uterosacral (L)	LCM	G12C	0·010	0*			Wildtype	
C_33	DIE94	DIE	Uterosacral (L)	LCM	G12D	0·043	2·7	0·90	3·96	Mutant	G12D
C_33	DIE94	DIE	Uterosacral (L)	LCM	G12R	0·0016	0*			Wildtype	
C_33	DIE94	DIE	Uterosacral (L)	LCM	G12V	0·010	0·011	0·010	0*	Wildtype	
C_33	DIE94	DIE	Uterosacral (L)	LCM	G12A	0·0046	0*			Wildtype	
C_33	DIE94	DIE	Uterosacral (L)	LCM	G12S	0·085	0·24	1·2	0·24	Wildtype	
C_33	DIE95	DIE	Uterosacral (L)	Macro	G12C	0·010	0*			Wildtype	
C_33	DIE95	DIE	Uterosacral (L)	Macro	G12D	0·042	0·94	0·69	1·5	Mutant	G12D
C_33	DIE95	DIE	Uterosacral (L)	Macro	G12R	0·0016	0*			Wildtype	
C_33	DIE95	DIE	Uterosacral (L)	Macro	G12V	0·010	0*			Wildtype	
C_33	DIE95	DIE	Uterosacral (L)	Macro	G12A	0·0046	0*			Wildtype	
C_33	DIE95	DIE	Uterosacral (L)	Macro	G12S	0·0084	0·55	0·53	0·70	Mutant	G12S
C_33	SUP12	SUP	Pelvic sidewall (R)	Macro	G12C	0·0041	0*			Wildtype	
C_33	SUP12	SUP	Pelvic sidewall (R)	Macro	G12D	0·018	0·042	0·73	0·40	Wildtype	
C_33	SUP12	SUP	Pelvic sidewall (R)	Macro	G12R	0·0014	0*			Wildtype	
C_33	SUP12	SUP	Pelvic sidewall (R)	Macro	G12V	0·0007	0·011	0·0060	0*	Wildtype	
C_33	SUP12	SUP	Pelvic sidewall (R)	Macro	G12A	0*	0*			Wildtype	

Case ID	Sample ID	Endo type	Anatomical Location	Enrich. method	Variant tested	Multi. cut-off VAF (%)	Multi. VAF (%)	Indiv. cut-off VAF (%)	Indiv. VAF (%)	Result	*KRAS* Variant
C_33	SUP12	SUP	Pelvic sidewall (R)	Macro	G12S	0·033	0·034	0·44	0·41	Wildtype	
C_33	SUP91	SUP	Pelvic sidewall (L)	Macro	G12C	0·0058	0*			Wildtype	
C_33	SUP91	SUP	Pelvic sidewall (L)	Macro	G12D	0·084	0·035			Wildtype	
C_33	SUP91	SUP	Pelvic sidewall (L)	Macro	G12R	0*	0*			Wildtype	
C_33	SUP91	SUP	Pelvic sidewall (L)	Macro	G12V	0·0036	0·0027			Wildtype	
C_33	SUP91	SUP	Pelvic sidewall (L)	Macro	G12A	0·0022	0*			Wildtype	
C_33	SUP91	SUP	Pelvic sidewall (L)	Macro	G12S	0·046	0·029			Wildtype	
C_34	OMA3	OMA	Ovary (L)	Macro	G12C	0*	0*			Wildtype	
C_34	OMA3	OMA	Ovary (L)	Macro	G12D	0·043	0·0032			Wildtype	
C_34	OMA3	OMA	Ovary (L)	Macro	G12R	0*	0*			Wildtype	
C_34	OMA3	OMA	Ovary (L)	Macro	G12V	0·0020	0·0016			Wildtype	
C_34	OMA3	OMA	Ovary (L)	Macro	G12A	0*	0*			Wildtype	
C_34	OMA3	OMA	Ovary (L)	Macro	G12S	0·031	0·020			Wildtype	
C_34	SUP13	SUP	Cul-de-sac	Macro	G12C	0·0041	0*			Wildtype	
C_34	SUP13	SUP	Cul-de-sac	Macro	G12D	0·018	0·0043			Wildtype	
C_34	SUP13	SUP	Cul-de-sac	Macro	G12R	0·0014	0*			Wildtype	
C_34	SUP13	SUP	Cul-de-sac	Macro	G12V	0·0007	0·0043	0·0060	0·0043	Wildtype	
C_34	SUP13	SUP	Cul-de-sac	Macro	G12A	0*	0*			Wildtype	
C_34	SUP13	SUP	Cul-de-sac	Macro	G12S	0·033	0·030			Wildtype	
C_35	OMA15	OMA	Ovary (R)	Macro	G12C	0·0019	0*			Wildtype	
C_35	OMA15	OMA	Ovary (R)	Macro	G12D	0·022	0·013			Wildtype	
C_35	OMA15	OMA	Ovary (R)	Macro	G12R	0*	0*			Wildtype	
C_35	OMA15	OMA	Ovary (R)	Macro	G12V	0·018	0·051	0·0016	0·49	Mutant	G12V
C_35	OMA15	OMA	Ovary (R)	Macro	G12A	0·0016	0·0033	0·0061	0*	Wildtype	
C_35	OMA15	OMA	Ovary (R)	Macro	G12S	0·025	0·053	1·0	0·38	Wildtype	
C_35	SUP14	SUP	Pelvic sidewall (L)	Macro	G12C	0·0041	0*			Wildtype	
C_35	SUP14	SUP	Pelvic sidewall (L)	Macro	G12D	0·018	0·035	0·73	0·39	Wildtype	
C_35	SUP14	SUP	Pelvic sidewall (L)	Macro	G12R	0·0014	0*			Wildtype	

Case ID	Sample ID	Endo type	Anatomical Location	Enrich. method	Variant tested	Multi. cut-off VAF (%)	Multi. VAF (%)	Indiv. cut-off VAF (%)	Indiv. VAF (%)	Result	*KRAS* Variant
C_35	SUP14	SUP	Pelvic sidewall (L)	Macro	G12V	0·0007	0·10	0·0060	1·7	Mutant	G12V
C_35	SUP14	SUP	Pelvic sidewall (L)	Macro	G12A	0*	0*			Wildtype	
C_35	SUP14	SUP	Pelvic sidewall (L)	Macro	G12S	0·033	0·011			Wildtype	
C_35	SUP92	SUP	Uterosacral (L)	Macro	G12C	0·0058	0*			Wildtype	
C_35	SUP92	SUP	Uterosacral (L)	Macro	G12D	0·084	0·037			Wildtype	
C_35	SUP92	SUP	Uterosacral (L)	Macro	G12R	0*	0*			Wildtype	
C_35	SUP92	SUP	Uterosacral (L)	Macro	G12V	0·0036	1·3	0·0053	11	Mutant	G12V
C_35	SUP92	SUP	Uterosacral (L)	Macro	G12A	0·0022	0·011	0·0015	0·025	Mutant	G12A
C_35	SUP92	SUP	Uterosacral (L)	Macro	G12S	0·046	0·039			Wildtype	
C_36	SUP15	SUP	Pelvic sidewall (L)	Macro	G12C	0·0041	0·0028			Wildtype	
C_36	SUP15	SUP	Pelvic sidewall (L)	Macro	G12D	0·018	0·033	0·73	0·33	Wildtype	
C_36	SUP15	SUP	Pelvic sidewall (L)	Macro	G12R	0·0014	0*			Wildtype	
C_36	SUP15	SUP	Pelvic sidewall (L)	Macro	G12V	0·0007	0·011	0·0060	0·014	Mutant	G12V
C_36	SUP15	SUP	Pelvic sidewall (L)	Macro	G12A	0*	0*			Wildtype	
C_36	SUP15	SUP	Pelvic sidewall (L)	Macro	G12S	0·033	0·039	0·44	0·47	Mutant	G12S
C_36	SUP93	SUP	Cul-de-sac (R)	Macro	G12C	0·0058	0*			Wildtype	
C_36	SUP93	SUP	Cul-de-sac (R)	Macro	G12D	0·084	0·053			Wildtype	
C_36	SUP93	SUP	Cul-de-sac (R)	Macro	G12R	0*	0*			Wildtype	
C_36	SUP93	SUP	Cul-de-sac (R)	Macro	G12V	0·0036	0·0023			Wildtype	
C_36	SUP93	SUP	Cul-de-sac (R)	Macro	G12A	0·0022	0*			Wildtype	
C_36	SUP93	SUP	Cul-de-sac (R)	Macro	G12S	0·046	0·030			Wildtype	
C_37	DIE86	DIE	Uterosacral (L)	LCM	G12C	0·010	0*			Wildtype	
C_37	DIE86	DIE	Uterosacral (L)	LCM	G12D	0·043	0*			Wildtype	
C_37	DIE86	DIE	Uterosacral (L)	LCM	G12R	0·0016	0*			Wildtype	
C_37	DIE86	DIE	Uterosacral (L)	LCM	G12V	0·010	0·0018			Wildtype	
C_37	DIE86	DIE	Uterosacral (L)	LCM	G12A	0·0046	0*			Wildtype	
C_37	DIE86	DIE	Uterosacral (L)	LCM	G12S	0·085	0·0035			Wildtype	
C_37	DIE87	DIE	Uterosacral (R)	Macro	G12C	0·010	0*			Wildtype	

Case ID	Sample ID	Endo type	Anatomical Location	Enrich. method	Variant tested	Multi. cut-off VAF (%)	Multi. VAF (%)	Indiv. cut-off VAF (%)	Indiv. VAF (%)	Result	*KRAS* Variant
C_37	DIE87	DIE	Uterosacral (R)	Macro	G12D	0·043	0·013			Wildtype	
C_37	DIE87	DIE	Uterosacral (R)	Macro	G12R	0·0016	0*			Wildtype	
C_37	DIE87	DIE	Uterosacral (R)	Macro	G12V	0·010	0*			Wildtype	
C_37	DIE87	DIE	Uterosacral (R)	Macro	G12A	0·0046	0·0053	0·0044	0·0077	Mutant	G12A
C_37	DIE87	DIE	Uterosacral (R)	Macro	G12S	0·085	0·013			Wildtype	
C_38	SUP16	SUP	Pelvic sidewall (L)	Macro	G12C	0·0041	0·0062	0·025	0·029	Mutant	G12C
C_38	SUP16	SUP	Pelvic sidewall (L)	Macro	G12D	0·018	0·031	0·73	0·24	Wildtype	
C_38	SUP16	SUP	Pelvic sidewall (L)	Macro	G12R	0·0014	0*			Wildtype	
C_38	SUP16	SUP	Pelvic sidewall (L)	Macro	G12V	0·0007	0*	0·0060	0·0048	Wildtype	
C_38	SUP16	SUP	Pelvic sidewall (L)	Macro	G12A	0*	0*			Wildtype	
C_38	SUP16	SUP	Pelvic sidewall (L)	Macro	G12S	0·033	0·093	0·44	0·29	Wildtype	
C_39	DIE1	DIE	Uterosacral (L)	LCM	G12C	0·017	0*			Wildtype	
C_39	DIE1	DIE	Uterosacral (L)	LCM	G12D	0·13	0·049			Wildtype	
C_39	DIE1	DIE	Uterosacral (L)	LCM	G12R	0*	0*			Wildtype	
C_39	DIE1	DIE	Uterosacral (L)	LCM	G12V	0·013	0·022	0·010	0*	Wildtype	
C_39	DIE1	DIE	Uterosacral (L)	LCM	G12A	0*	0*			Wildtype	
C_39	DIE1	DIE	Uterosacral (L)	LCM	G12S	0·045	0·14	0·67	0·27	Wildtype	
C_39	DIE2	DIE	Uterosacral (L)	LCM	G12C	0·0020	0·010	0·55	0·10	Wildtype	
C_39	DIE2	DIE	Uterosacral (L)	LCM	G12D	0·0020	0·015	0·77	0·20	Wildtype	
C_39	DIE2	DIE	Uterosacral (L)	LCM	G12R	0*	0·0042	0·0020	0*	Wildtype	
C_39	DIE2	DIE	Uterosacral (L)	LCM	G12V	0·0060	0·0042			Wildtype	
C_39	DIE2	DIE	Uterosacral (L)	LCM	G12A	0·0060	0*			Wildtype	
C_39	DIE2	DIE	Uterosacral (L)	LCM	G12S	0·0020	0*			Wildtype	
C_39	DIE3	DIE	Uterosacral (L)	Macro	G12C	0·0020	0*			Wildtype	
C_39	DIE3	DIE	Uterosacral (L)	Macro	G12D	0·0020	0*	0·77	0·58	Wildtype	
C_39	DIE3	DIE	Uterosacral (L)	Macro	G12R	0*	0*			Wildtype	
C_39	DIE3	DIE	Uterosacral (L)	Macro	G12V	0·0060	0·0015			Wildtype	
C_39	DIE3	DIE	Uterosacral (L)	Macro	G12A	0·0060	0·0015	0·0015	0·58	Mutant	G12A

Case ID	Sample ID	Endo type	Anatomical Location	Enrich. method	Variant tested	Multi. cut-off VAF (%)	Multi. VAF (%)	Indiv. cut-off VAF (%)	Indiv. VAF (%)	Result	*KRAS* Variant
C_39	DIE3	DIE	Uterosacral (L)	Macro	G12S	0·0020	0·0015	0·67	0·33	Wildtype	
C_39	SUP17	SUP	Pelvic sidewall (R)	Macro	G12C	0·0019	0·0021	0·039	0·020	Wildtype	
C_39	SUP17	SUP	Pelvic sidewall (R)	Macro	G12D	0·022	0·0093			Wildtype	
C_39	SUP17	SUP	Pelvic sidewall (R)	Macro	G12R	0*	0*			Wildtype	
C_39	SUP17	SUP	Pelvic sidewall (R)	Macro	G12V	0·018	0*			Wildtype	
C_39	SUP17	SUP	Pelvic sidewall (R)	Macro	G12A	0·0016	0*			Wildtype	
C_39	SUP17	SUP	Pelvic sidewall (R)	Macro	G12S	0·025	0·0046			Wildtype	
C_40	DIE79	DIE	Cul-de-sac	Macro	G12C	0·0020	0·0018			Wildtype	
C_40	DIE79	DIE	Cul-de-sac	Macro	G12D	0·0020	0·0035	0·77	0·26	Wildtype	
C_40	DIE79	DIE	Cul-de-sac	Macro	G12R	0*	0*			Wildtype	
C_40	DIE79	DIE	Cul-de-sac	Macro	G12V	0·0060	0·0018			Wildtype	
C_40	DIE79	DIE	Cul-de-sac	Macro	G12A	0·0060	0*			Wildtype	
C_40	DIE79	DIE	Cul-de-sac	Macro	G12S	0·0020	0·0035	0·73	0·30	Wildtype	
C_41	OMA1	OMA	Ovary (NS)	Macro	G12C	0·010	0*			Wildtype	
C_41	OMA1	OMA	Ovary (NS)	Macro	G12D	0·043	0·041			Wildtype	
C_41	OMA1	OMA	Ovary (NS)	Macro	G12R	0·0016	0*			Wildtype	
C_41	OMA1	OMA	Ovary (NS)	Macro	G12V	0·010	0·0094			Wildtype	
C_41	OMA1	OMA	Ovary (NS)	Macro	G12A	0·0046	0·20	0·0033	1·2	Mutant	G12A
C_41	OMA1	OMA	Ovary (NS)	Macro	G12S	0·085	0·044	1·2	0·25	Wildtype	
C_42	DIE102	DIE	Rectosigmoid junction	LCM	G12C	0·010	0*			Wildtype	
C_42	DIE102	DIE	Rectosigmoid junction	LCM	G12D	0·043	0·0016			Wildtype	
C_42	DIE102	DIE	Rectosigmoid junction	LCM	G12R	0·0016	0*			Wildtype	
C_42	DIE102	DIE	Rectosigmoid junction	LCM	G12V	0·010	0*			Wildtype	
C_42	DIE102	DIE	Rectosigmoid junction	LCM	G12A	0·0046	0·0016			Wildtype	
C_42	DIE102	DIE	Rectosigmoid junction	LCM	G12S	0·085	0*			Wildtype	
C_43	SUP18	SUP	Cul-de-sac (R)	Macro	G12C	0·0019	0*			Wildtype	
C_43	SUP18	SUP	Cul-de-sac (R)	Macro	G12D	0·022	0·0021			Wildtype	

Case ID	Sample ID	Endo type	Anatomical Location	Enrich. method	Variant tested	Multi. cut-off VAF (%)	Multi. VAF (%)	Indiv. cut-off VAF (%)	Indiv. VAF (%)	Result	*KRAS* Variant
C_43	SUP18	SUP	Cul-de-sac (R)	Macro	G12R	0*	0*			Wildtype	
C_43	SUP18	SUP	Cul-de-sac (R)	Macro	G12V	0·018	0·013			Wildtype	
C_43	SUP18	SUP	Cul-de-sac (R)	Macro	G12A	0·0016	0·0021	0·0061	0·0015	Wildtype	
C_43	SUP18	SUP	Cul-de-sac (R)	Macro	G12S	0·025	0*			Wildtype	
C_43	SUP94	SUP	Cul-de-sac (L)	Macro	G12C	0*	0*			Wildtype	
C_43	SUP94	SUP	Cul-de-sac (L)	Macro	G12D	0·043	0*			Wildtype	
C_43	SUP94	SUP	Cul-de-sac (L)	Macro	G12R	0*	0*			Wildtype	
C_43	SUP94	SUP	Cul-de-sac (L)	Macro	G12V	0·0020	0*			Wildtype	
C_43	SUP94	SUP	Cul-de-sac (L)	Macro	G12A	0*	0*			Wildtype	
C_43	SUP94	SUP	Cul-de-sac (L)	Macro	G12S	0·031	0*			Wildtype	
C_44	SUP19	SUP	Cul-de-sac	Macro	G12C	0·0019	0·0024	0·039	0·0016	Wildtype	
C_44	SUP19	SUP	Cul-de-sac	Macro	G12D	0·022	0·0071			Wildtype	
C_44	SUP19	SUP	Cul-de-sac	Macro	G12R	0*	0*			Wildtype	
C_44	SUP19	SUP	Cul-de-sac	Macro	G12V	0·018	0·0070			Wildtype	
C_44	SUP19	SUP	Cul-de-sac	Macro	G12A	0·0016	0*			Wildtype	
C_44	SUP19	SUP	Cul-de-sac	Macro	G12S	0·025	0·012			Wildtype	
C_45	SUP20	SUP	Uterosacral (R)	Macro	G12C	0*	0*			Wildtype	
C_45	SUP20	SUP	Uterosacral (R)	Macro	G12D	0·019	0·044	0·99	0·42	Wildtype	
C_45	SUP20	SUP	Uterosacral (R)	Macro	G12R	0*	0*			Wildtype	
C_45	SUP20	SUP	Uterosacral (R)	Macro	G12V	0·0016	0·0044	0·017	0·0046	Wildtype	
C_45	SUP20	SUP	Uterosacral (R)	Macro	G12A	0·0016	0*			Wildtype	
C_45	SUP20	SUP	Uterosacral (R)	Macro	G12S	0·030	0·044	1·0	0·41	Wildtype	
C_45	SUP95	SUP	Cul-de-sac (L)	Macro	G12C	0·0058	0·42	0·072	8·4	Mutant	G12C
C_45	SUP95	SUP	Cul-de-sac (L)	Macro	G12D	0·084	0·15	0·98	0·23	Wildtype	
C_45	SUP95	SUP	Cul-de-sac (L)	Macro	G12R	0*	0·015	0·0020	0·010	Mutant	G12R
C_45	SUP95	SUP	Cul-de-sac (L)	Macro	G12V	0·0036	0·0097	0·0053	0·0012	Wildtype	
C_45	SUP95	SUP	Cul-de-sac (L)	Macro	G12A	0·0022	0*			Wildtype	
C_45	SUP95	SUP	Cul-de-sac (L)	Macro	G12S	0·046	0·0096			Wildtype	

Case ID	Sample ID	Endo type	Anatomical Location	Enrich. method	Variant tested	Multi. cut-off VAF (%)	Multi. VAF (%)	Indiv. cut-off VAF (%)	Indiv. VAF (%)	Result	*KRAS* Variant
C_46	OMA17	OMA	Ovary (L)	Macro	G12C	0·012	0·037	0·036	0*	Wildtype	
C_46	OMA17	OMA	Ovary (L)	Macro	G12D	0·017	0·036	0·71	0·31	Wildtype	
C_46	OMA17	OMA	Ovary (L)	Macro	G12R	0*	0*			Wildtype	
C_46	OMA17	OMA	Ovary (L)	Macro	G12V	0·0097	0*			Wildtype	
C_46	OMA17	OMA	Ovary (L)	Macro	G12A	0*	0*			Wildtype	
C_46	OMA17	OMA	Ovary (L)	Macro	G12S	0·038	0·088	0·75	0·24	Wildtype	
C_47	SUP21	SUP	Cul-de-sac (R)	Macro	G12C	0·0041	0·019	0·025	0·0060	Wildtype	
C_47	SUP21	SUP	Cul-de-sac (R)	Macro	G12D	0·018	0·096	0·73	0·29	Wildtype	
C_47	SUP21	SUP	Cul-de-sac (R)	Macro	G12R	0·0014	0*			Wildtype	
C_47	SUP21	SUP	Cul-de-sac (R)	Macro	G12V	0·0007	0*			Wildtype	
C_47	SUP21	SUP	Cul-de-sac (R)	Macro	G12A	0*	0·0064	0*	0*	Wildtype	
C_47	SUP21	SUP	Cul-de-sac (R)	Macro	G12S	0·033	0·14	0·44	0·46	Mutant	G12S
C_47	SUP96	SUP	Cul-de-sac (L)	Macro	G12C	0·0058	0·0029			Wildtype	
C_47	SUP96	SUP	Cul-de-sac (L)	Macro	G12D	0·084	0·015			Wildtype	
C_47	SUP96	SUP	Cul-de-sac (L)	Macro	G12R	0*	0*			Wildtype	
C_47	SUP96	SUP	Cul-de-sac (L)	Macro	G12V	0·0036	0·40	0·0053	2·2	Mutant	G12V
C_47	SUP96	SUP	Cul-de-sac (L)	Macro	G12A	0·0022	0·0030	0·0015	0·0028	Mutant	G12A
C_47	SUP96	SUP	Cul-de-sac (L)	Macro	G12S	0·046	0·044			Wildtype	
C_48	DIE97	DIE	Uterosacral (L)	LCM	G12C	0·010	0*			Wildtype	
C_48	DIE97	DIE	Uterosacral (L)	LCM	G12D	0·043	0·047	0·74	0·31	Wildtype	
C_48	DIE97	DIE	Uterosacral (L)	LCM	G12R	0·0016	0*			Wildtype	
C_48	DIE97	DIE	Uterosacral (L)	LCM	G12V	0·010	0·0030			Wildtype	
C_48	DIE97	DIE	Uterosacral (L)	LCM	G12A	0·0046	0*			Wildtype	
C_48	DIE97	DIE	Uterosacral (L)	LCM	G12S	0·085	0·045			Wildtype	
C_48	DIE98	DIE	Uterosacral (L)	LCM	G12C	0·010	0*			Wildtype	
C_48	DIE98	DIE	Uterosacral (L)	LCM	G12D	0·043	0·015			Wildtype	
C_48	DIE98	DIE	Uterosacral (L)	LCM	G12R	0·0016	0*			Wildtype	
C_48	DIE98	DIE	Uterosacral (L)	LCM	G12V	0·010	0·0088			Wildtype	

Case ID	Sample ID	Endo type	Anatomical Location	Enrich. method	Variant tested	Multi. cut-off VAF (%)	Multi. VAF (%)	Indiv. cut-off VAF (%)	Indiv. VAF (%)	Result	*KRAS* Variant
C_48	DIE98	DIE	Uterosacral (L)	LCM	G12A	0·0046	0·0029			Wildtype	
C_48	DIE98	DIE	Uterosacral (L)	LCM	G12S	0·085	0·020			Wildtype	
C_48	OMA18	OMA	Ovary (R)	Macro	G12C	0·012	0·040	0·036	0·018	Wildtype	
C_48	OMA18	OMA	Ovary (R)	Macro	G12D	0·017	0·048	0·71	0·29	Wildtype	
C_48	OMA18	OMA	Ovary (R)	Macro	G12R	0*	0*			Wildtype	
C_48	OMA18	OMA	Ovary (R)	Macro	G12V	0·0097	0·0044	0·0060	0·0038	Wildtype	
C_48	OMA18	OMA	Ovary (R)	Macro	G12A	0*	0*			Wildtype	
C_48	OMA18	OMA	Ovary (R)	Macro	G12S	0·038	0·013			Wildtype	
C_48	SUP48	SUP	Fallopian tube	Macro	G12C	0*	0*			Wildtype	
C_48	SUP48	SUP	Fallopian tube	Macro	G12D	0·027	0·021			Wildtype	
C_48	SUP48	SUP	Fallopian tube	Macro	G12R	0*	0*			Wildtype	
C_48	SUP48	SUP	Fallopian tube	Macro	G12V	0*	0·032	0·0065	0·21	Mutant	G12V
C_48	SUP48	SUP	Fallopian tube	Macro	G12A	0·0021	0*			Wildtype	
C_48	SUP48	SUP	Fallopian tube	Macro	G12S	0·021	0·016			Wildtype	
C_49	DIE59	DIE	Cul-de-sac	Macro	G12C	0·24	0·018			Wildtype	
C_49	DIE59	DIE	Cul-de-sac	Macro	G12D	0·24	0·018			Wildtype	
C_49	DIE59	DIE	Cul-de-sac	Macro	G12R	0·0048	0*			Wildtype	
C_49	DIE59	DIE	Cul-de-sac	Macro	G12V	0·0060	0·0044			Wildtype	
C_49	DIE59	DIE	Cul-de-sac	Macro	G12A	0*	0*			Wildtype	
C_49	DIE59	DIE	Cul-de-sac	Macro	G12S	0·23	0·027			Wildtype	
C_50	OMA16	OMA	Ovary (L)	Macro	G12C	0·0019	0·0074	0·039	0·011	Wildtype	
C_50	OMA16	OMA	Ovary (L)	Macro	G12D	0·022	0·073	0·64	0·74	Mutant	G12D
C_50	OMA16	OMA	Ovary (L)	Macro	G12R	0*	0*			Wildtype	
C_50	OMA16	OMA	Ovary (L)	Macro	G12V	0·018	0·037	0·0016	0·077	Mutant	G12V
C_50	OMA16	OMA	Ovary (L)	Macro	G12A	0·0016	0·0037	0·0061	0*	Wildtype	
C_50	OMA16	OMA	Ovary (L)	Macro	G12S	0·025	0·0073			Wildtype	
C_51	DIE28	DIE	Cul-de-sac	Macro	G12C	0·0020	0·068	0·55	0·34	Wildtype	
C_51	DIE28	DIE	Cul-de-sac	Macro	G12D	0·0020	0·13	0·77	0·36	Wildtype	

Case ID	Sample ID	Endo type	Anatomical Location	Enrich. method	Variant tested	Multi. cut-off VAF (%)	Multi. VAF (%)	Indiv. cut-off VAF (%)	Indiv. VAF (%)	Result	*KRAS* Variant
C_51	DIE28	DIE	Cul-de-sac	Macro	G12R	0*	0*			Wildtype	
C_51	DIE28	DIE	Cul-de-sac	Macro	G12V	0·0060	0·015	0·010	0*	Wildtype	
C_51	DIE28	DIE	Cul-de-sac	Macro	G12A	0·0060	0*			Wildtype	
C_51	DIE28	DIE	Cul-de-sac	Macro	G12S	0·0020	0·13	0·67	0·40	Wildtype	
C_51	OMA4	OMA	Ovary (R)	Macro	G12C	0*	0*			Wildtype	
C_51	OMA4	OMA	Ovary (R)	Macro	G12D	0·027	0·014			Wildtype	
C_51	OMA4	OMA	Ovary (R)	Macro	G12R	0*	0*			Wildtype	
C_51	OMA4	OMA	Ovary (R)	Macro	G12V	0*	0*			Wildtype	
C_51	OMA4	OMA	Ovary (R)	Macro	G12A	0·0021	0*			Wildtype	
C_51	OMA4	OMA	Ovary (R)	Macro	G12S	0·021	0·011			Wildtype	
C_52	SUP76	SUP	Fallopian tube (R)	Macro	G12C	0*	0*			Wildtype	
C_52	SUP76	SUP	Fallopian tube (R)	Macro	G12D	0·043	0*			Wildtype	
C_52	SUP76	SUP	Fallopian tube (R)	Macro	G12R	0*	0*			Wildtype	
C_52	SUP76	SUP	Fallopian tube (R)	Macro	G12V	0·0020	0*			Wildtype	
C_52	SUP76	SUP	Fallopian tube (R)	Macro	G12A	0*	0*			Wildtype	
C_52	SUP76	SUP	Fallopian tube (R)	Macro	G12S	0·031	0*			Wildtype	
C_53	SUP22	SUP	Fallopian tube (B)	Macro	G12C	0*	0*			Wildtype	
C_53	SUP22	SUP	Fallopian tube (B)	Macro	G12D	0·019	0·026	0·99	0·84	Wildtype	
C_53	SUP22	SUP	Fallopian tube (B)	Macro	G12R	0*	0*			Wildtype	
C_53	SUP22	SUP	Fallopian tube (B)	Macro	G12V	0·0016	0*			Wildtype	
C_53	SUP22	SUP	Fallopian tube (B)	Macro	G12A	0·0016	0·0029	0·0044	0·0017	Wildtype	
C_53	SUP22	SUP	Fallopian tube (B)	Macro	G12S	0·030	0·0088			Wildtype	
C_54	SUP23	SUP	Cul-de-sac	Macro	G12C	0*	0*			Wildtype	
C_54	SUP23	SUP	Cul-de-sac	Macro	G12D	0·019	0·028	0·99	0·30	Wildtype	
C_54	SUP23	SUP	Cul-de-sac	Macro	G12R	0*	0*			Wildtype	
C_54	SUP23	SUP	Cul-de-sac	Macro	G12V	0·0016	0·0046	0·017	0·0040	Wildtype	
C_54	SUP23	SUP	Cul-de-sac	Macro	G12A	0·0016	0*			Wildtype	
C_54	SUP23	SUP	Cul-de-sac	Macro	G12S	0·030	0·019			Wildtype	

Case ID	Sample ID	Endo type	Anatomical Location	Enrich. method	Variant tested	Multi. cut-off VAF (%)	Multi. VAF (%)	Indiv. cut-off VAF (%)	Indiv. VAF (%)	Result	*KRAS* Variant
C_55	DIE22	DIE	Rectosigmoid junction	Macro	G12C	0·0020	0*			Wildtype	
C_55	DIE22	DIE	Rectosigmoid junction	Macro	G12D	0·0020	0*			Wildtype	
C_55	DIE22	DIE	Rectosigmoid junction	Macro	G12R	0*	0*			Wildtype	
C_55	DIE22	DIE	Rectosigmoid junction	Macro	G12V	0·0060	0*			Wildtype	
C_55	DIE22	DIE	Rectosigmoid junction	Macro	G12A	0·0060	0*			Wildtype	
C_55	DIE22	DIE	Rectosigmoid junction	Macro	G12S	0·0020	0*			Wildtype	
C_55	DIE23	DIE	Appendix	Macro	G12C	0·0020	0*			Wildtype	
C_55	DIE23	DIE	Appendix	Macro	G12D	0·0020	0·0033	0·77	0·19	Wildtype	
C_55	DIE23	DIE	Appendix	Macro	G12R	0*	0*			Wildtype	
C_55	DIE23	DIE	Appendix	Macro	G12V	0·0060	0*			Wildtype	
C_55	DIE23	DIE	Appendix	Macro	G12A	0·0060	0*			Wildtype	
C_55	DIE23	DIE	Appendix	Macro	G12S	0·0020	0·0033	0·67	0·36	Wildtype	
C_55	DIE24	DIE	Uterus	LCM	G12C	0·010	0*			Wildtype	
C_55	DIE24	DIE	Uterus	LCM	G12D	0·043	0·0089			Wildtype	
C_55	DIE24	DIE	Uterus	LCM	G12R	0·0016	0*			Wildtype	
C_55	DIE24	DIE	Uterus	LCM	G12V	0·010	0·0022			Wildtype	
C_55	DIE24	DIE	Uterus	LCM	G12A	0·0046	0*			Wildtype	
C_55	DIE24	DIE	Uterus	LCM	G12S	0·085	0·016			Wildtype	
C_55	DIE25	DIE	Uterus	Macro	G12C	0·24	0·0087			Wildtype	
C_55	DIE25	DIE	Uterus	Macro	G12D	0·24	0·0087			Wildtype	
C_55	DIE25	DIE	Uterus	Macro	G12R	0·0048	0*			Wildtype	
C_55	DIE25	DIE	Uterus	Macro	G12V	0·0060	0·0052			Wildtype	
C_55	DIE25	DIE	Uterus	Macro	G12A	0*	0*			Wildtype	
C_55	DIE25	DIE	Uterus	Macro	G12S	0·23	0·0017			Wildtype	
C_55	DIE26	DIE	Uterosacral (B)	LCM	G12C	0·010	0*			Wildtype	
C_55	DIE26	DIE	Uterosacral (B)	LCM	G12D	0·043	0·0027			Wildtype	
C_55	DIE26	DIE	Uterosacral (B)	LCM	G12R	0·0016	0*			Wildtype	

Case ID	Sample ID	Endo type	Anatomical Location	Enrich. method	Variant tested	Multi. cut-off VAF (%)	Multi. VAF (%)	Indiv. cut-off VAF (%)	Indiv. VAF (%)	Result	*KRAS* Variant
C_55	DIE26	DIE	Uterosacral (B)	LCM	G12V	0·010	0·017	0·11	0·068	Wildtype	
C_55	DIE26	DIE	Uterosacral (B)	LCM	G12A	0·0046	0*			Wildtype	
C_55	DIE26	DIE	Uterosacral (B)	LCM	G12S	0·085	0·0082			Wildtype	
C_56	OMA5	OMA	Ovary (L)	Macro	G12C	0*	0*			Wildtype	
C_56	OMA5	OMA	Ovary (L)	Macro	G12D	0·027	0·013			Wildtype	
C_56	OMA5	OMA	Ovary (L)	Macro	G12R	0*	0*			Wildtype	
C_56	OMA5	OMA	Ovary (L)	Macro	G12V	0*	0*			Wildtype	
C_56	OMA5	OMA	Ovary (L)	Macro	G12A	0·0021	0*			Wildtype	
C_56	OMA5	OMA	Ovary (L)	Macro	G12S	0·021	0·021	0·44	0·41	Wildtype	
C_56	SUP24	SUP	Uterosacral (R)	Macro	G12C	0*	0*			Wildtype	
C_56	SUP24	SUP	Uterosacral (R)	Macro	G12D	0·019	0·0021	0·99	0·32	Wildtype	
C_56	SUP24	SUP	Uterosacral (R)	Macro	G12R	0*	0*			Wildtype	
C_56	SUP24	SUP	Uterosacral (R)	Macro	G12V	0·0016	0·015	0·017	0·31	Mutant	G12V
C_56	SUP24	SUP	Uterosacral (R)	Macro	G12A	0·0016	0*			Wildtype	
C_56	SUP24	SUP	Uterosacral (R)	Macro	G12S	0·030	0·0062			Wildtype	
C_56	SUP97	SUP	Uterosacral (L)	Macro	G12C	0*	0*			Wildtype	
C_56	SUP97	SUP	Uterosacral (L)	Macro	G12D	0·043	0·024	1·5	0·32	Wildtype	
C_56	SUP97	SUP	Uterosacral (L)	Macro	G12R	0*	0*			Wildtype	
C_56	SUP97	SUP	Uterosacral (L)	Macro	G12V	0·0020	0·0069	0·0039	0*	Wildtype	
C_56	SUP97	SUP	Uterosacral (L)	Macro	G12A	0*	0*			Wildtype	
C_56	SUP97	SUP	Uterosacral (L)	Macro	G12S	0·031	0·036	0·78	0·41	Wildtype	
C_57	DIE16	DIE	Uterosacral (L)	LCM	G12C	0·010	0·0096			Wildtype	
C_57	DIE16	DIE	Uterosacral (L)	LCM	G12D	0·043	0·038			Wildtype	
C_57	DIE16	DIE	Uterosacral (L)	LCM	G12R	0·0016	0*			Wildtype	
C_57	DIE16	DIE	Uterosacral (L)	LCM	G12V	0·010	0·014	0·010	0·029	Mutant	G12V
C_57	DIE16	DIE	Uterosacral (L)	LCM	G12A	0·0046	0*			Wildtype	
C_57	DIE16	DIE	Uterosacral (L)	LCM	G12S	0·085	0·057			Wildtype	
C_57	DIE17	DIE	Uterosacral (R)	Macro	G12C	0·017	0*			Wildtype	

Case ID	Sample ID	Endo type	Anatomical Location	Enrich. method	Variant tested	Multi. cut-off VAF (%)	Multi. VAF (%)	Indiv. cut-off VAF (%)	Indiv. VAF (%)	Result	*KRAS* Variant
C_57	DIE17	DIE	Uterosacral (R)	Macro	G12D	0·13	0·042	0·77	0·28	Wildtype	
C_57	DIE17	DIE	Uterosacral (R)	Macro	G12R	0*	0*			Wildtype	
C_57	DIE17	DIE	Uterosacral (R)	Macro	G12V	0·0060	0*			Wildtype	
C_57	DIE17	DIE	Uterosacral (R)	Macro	G12A	0*	0*			Wildtype	
C_57	DIE17	DIE	Uterosacral (R)	Macro	G12S	0·045	0·34	0·67	0·33	Wildtype	
C_57	DIE18	DIE	Cul-de-sac	Macro	G12C	0·238	0·200			Wildtype	
C_57	DIE18	DIE	Cul-de-sac	Macro	G12D	0·24	0·26	0·35	0·48	Mutant	G12D
C_57	DIE18	DIE	Cul-de-sac	Macro	G12R	0·0048	0*			Wildtype	
C_57	DIE18	DIE	Cul-de-sac	Macro	G12V	0·0060	0*			Wildtype	
C_57	DIE18	DIE	Cul-de-sac	Macro	G12A	0*	0*			Wildtype	
C_57	DIE18	DIE	Cul-de-sac	Macro	G12S	0·23	0·037			Wildtype	
C_57	DIE19	DIE	Vagina	LCM	G12C	0·010	0*			Wildtype	
C_57	DIE19	DIE	Vagina	LCM	G12D	0·043	0·014			Wildtype	
C_57	DIE19	DIE	Vagina	LCM	G12R	0·0016	0*			Wildtype	
C_57	DIE19	DIE	Vagina	LCM	G12V	0·010	0*			Wildtype	
C_57	DIE19	DIE	Vagina	LCM	G12A	0·0046	0*			Wildtype	
C_57	DIE19	DIE	Vagina	LCM	G12S	0·085	0·036			Wildtype	
C_57	DIE20	DIE	Cul-de-sac	Macro	G12C	0·24	0·0032			Wildtype	
C_57	DIE20	DIE	Cul-de-sac	Macro	G12D	0·24	0·0032			Wildtype	
C_57	DIE20	DIE	Cul-de-sac	Macro	G12R	0·0048	0*			Wildtype	
C_57	DIE20	DIE	Cul-de-sac	Macro	G12V	0·0060	0·0032			Wildtype	
C_57	DIE20	DIE	Cul-de-sac	Macro	G12A	0*	0*			Wildtype	
C_57	DIE20	DIE	Cul-de-sac	Macro	G12S	0·23	0·0095			Wildtype	
C_58	DIE103	DIE	Cul-de-sac	LCM	G12C	0·010	0*			Wildtype	
C_58	DIE103	DIE	Cul-de-sac	LCM	G12D	0·043	0·49	0·74	0·85	Mutant	G12D
C_58	DIE103	DIE	Cul-de-sac	LCM	G12R	0·0016	0*			Wildtype	
C_58	DIE103	DIE	Cul-de-sac	LCM	G12V	0·010	0*			Wildtype	
C_58	DIE103	DIE	Cul-de-sac	LCM	G12A	0·0046	0*			Wildtype	

Case ID	Sample ID	Endo type	Anatomical Location	Enrich. method	Variant tested	Multi. cut-off VAF (%)	Multi. VAF (%)	Indiv. cut-off VAF (%)	Indiv. VAF (%)	Result	*KRAS* Variant
C_58	DIE103	DIE	Cul-de-sac	LCM	G12S	0·085	0·023			Wildtype	
C_59	DIE89	DIE	Uterosacral (R)	LCM	G12C	0·010	0*	0·030	0*	Wildtype	
C_59	DIE89	DIE	Uterosacral (R)	LCM	G12D	0·043	0*	1·0	0·14	Wildtype	
C_59	DIE89	DIE	Uterosacral (R)	LCM	G12R	0·0016	0*	0·020	0*	Wildtype	
C_59	DIE89	DIE	Uterosacral (R)	LCM	G12V	0·010	0*	0·010	0*	Wildtype	
C_59	DIE89	DIE	Uterosacral (R)	LCM	G12A	0·0046	0*	0·0049	0*	Wildtype	
C_59	DIE89	DIE	Uterosacral (R)	LCM	G12S	0·0084	0·73	0·53	1·3	Mutant	G12S
C_59	DIE90	DIE	Uterosacral (R)	LCM	G12C	0·010	0*			Wildtype	
C_59	DIE90	DIE	Uterosacral (R)	LCM	G12D	0·043	0·17	0·74	0·34	Wildtype	
C_59	DIE90	DIE	Uterosacral (R)	LCM	G12R	0·0016	0*			Wildtype	
C_59	DIE90	DIE	Uterosacral (R)	LCM	G12V	0·010	0·020	0·010	0*	Wildtype	
C_59	DIE90	DIE	Uterosacral (R)	LCM	G12A	0·0046	0·0098	0·0029	0*	Wildtype	
C_59	DIE90	DIE	Uterosacral (R)	LCM	G12S	0·085	0·17	1·2	0·46	Wildtype	
C_59	DIE91	DIE	Uterosacral (L)	Macro	G12C	0·010	0·0055			Wildtype	
C_59	DIE91	DIE	Uterosacral (L)	Macro	G12D	0·043	0·033			Wildtype	
C_59	DIE91	DIE	Uterosacral (L)	Macro	G12R	0·0016	0*			Wildtype	
C_59	DIE91	DIE	Uterosacral (L)	Macro	G12V	0·010	0·017	0·010	0·15	Mutant	G12V
C_59	DIE91	DIE	Uterosacral (L)	Macro	G12A	0·0046	1·6	0·0033	4·6	Mutant	G12A
C_59	DIE91	DIE	Uterosacral (L)	Macro	G12S	0·085	0·24	1·2	0·38	Wildtype	
C_59	OMA31	OMA	Ovary (R)	Macro	G12C	0*	0*			Wildtype	
C_59	OMA31	OMA	Ovary (R)	Macro	G12D	0·043	0·0019			Wildtype	
C_59	OMA31	OMA	Ovary (R)	Macro	G12R	0*	0*			Wildtype	
C_59	OMA31	OMA	Ovary (R)	Macro	G12V	0·0020	0·0038	0·0039	0*	Wildtype	
C_59	OMA31	OMA	Ovary (R)	Macro	G12A	0*	0*			Wildtype	
C_59	OMA31	OMA	Ovary (R)	Macro	G12S	0·031	0·012			Wildtype	
C_60	SUP25	SUP	Uterus (P)	Macro	G12C	0*	0*			Wildtype	
C_60	SUP25	SUP	Uterus (P)	Macro	G12D	0·019	0·026	0·99	0·35	Wildtype	
C_60	SUP25	SUP	Uterus (P)	Macro	G12R	0*	0*			Wildtype	

Case ID	Sample ID	Endo type	Anatomical Location	Enrich. method	Variant tested	Multi. cut-off VAF (%)	Multi. VAF (%)	Indiv. cut-off VAF (%)	Indiv. VAF (%)	Result	*KRAS* Variant
C_60	SUP25	SUP	Uterus (P)	Macro	G12V	0·0016	0*			Wildtype	
C_60	SUP25	SUP	Uterus (P)	Macro	G12A	0·0016	0·0028	0·0044	0·0031	Wildtype	
C_60	SUP25	SUP	Uterus (P)	Macro	G12S	0·030	0·051	1·0	0·30	Wildtype	
C_61	SUP26	SUP	Cul-de-sac	Macro	G12C	0*	0*			Wildtype	
C_61	SUP26	SUP	Cul-de-sac	Macro	G12D	0·019	0*			Wildtype	
C_61	SUP26	SUP	Cul-de-sac	Macro	G12R	0*	0*			Wildtype	
C_61	SUP26	SUP	Cul-de-sac	Macro	G12V	0·0016	0*			Wildtype	
C_61	SUP26	SUP	Cul-de-sac	Macro	G12A	0·0016	0*			Wildtype	
C_61	SUP26	SUP	Cul-de-sac	Macro	G12S	0·030	0·067	1·0	0·34	Wildtype	
C_62	DIE50	DIE	Uterosacral (R)	Macro	G12C	0·24	0·14			Wildtype	
C_62	DIE50	DIE	Uterosacral (R)	Macro	G12D	0·24	0·13	0·35	0·70	Mutant	G12D
C_62	DIE50	DIE	Uterosacral (R)	Macro	G12R	0·0048	0*			Wildtype	
C_62	DIE50	DIE	Uterosacral (R)	Macro	G12V	0·0060	0·010	0·010	0*	Wildtype	
C_62	DIE50	DIE	Uterosacral (R)	Macro	G12A	0*	0·0050	0·0049	0*	Wildtype	
C_62	DIE50	DIE	Uterosacral (R)	Macro	G12S	0·23	0·14	0·67	0·43	Wildtype	
C_62	SUP27	SUP	Cul-de-sac	Macro	G12C	0*	0*			Wildtype	
C_62	SUP27	SUP	Cul-de-sac	Macro	G12D	0·019	0·049	0·99	0·76	Wildtype	
C_62	SUP27	SUP	Cul-de-sac	Macro	G12R	0*	0*			Wildtype	
C_62	SUP27	SUP	Cul-de-sac	Macro	G12V	0·0016	0·0065	0·017	0·0033	Wildtype	
C_62	SUP27	SUP	Cul-de-sac	Macro	G12A	0·0016	0*			Wildtype	
C_62	SUP27	SUP	Cul-de-sac	Macro	G12S	0·030	0·042	1·0	0·31	Wildtype	
C_62	SUP98	SUP	Uterosacral (L)	Macro	G12C	0·0058	0*			Wildtype	
C_62	SUP98	SUP	Uterosacral (L)	Macro	G12D	0·084	0*			Wildtype	
C_62	SUP98	SUP	Uterosacral (L)	Macro	G12R	0*	0*			Wildtype	
C_62	SUP98	SUP	Uterosacral (L)	Macro	G12V	0·0036	0*			Wildtype	
C_62	SUP98	SUP	Uterosacral (L)	Macro	G12A	0·0022	0*			Wildtype	
C_62	SUP98	SUP	Uterosacral (L)	Macro	G12S	0·046	0*			Wildtype	
C_63	OMA19	OMA	Ovary (NS)	Macro	G12C	0·012	0·018	0·036	0*	Wildtype	

Case ID	Sample ID	Endo type	Anatomical Location	Enrich. method	Variant tested	Multi. cut-off VAF (%)	Multi. VAF (%)	Indiv. cut-off VAF (%)	Indiv. VAF (%)	Result	*KRAS* Variant
C_63	OMA19	OMA	Ovary (NS)	Macro	G12D	0·017	0·018	0·71	0·23	Wildtype	
C_63	OMA19	OMA	Ovary (NS)	Macro	G12R	0*	0*			Wildtype	
C_63	OMA19	OMA	Ovary (NS)	Macro	G12V	0·0097	0·052	0*	0·36	Mutant	G12V
C_63	OMA19	OMA	Ovary (NS)	Macro	G12A	0*	0*			Wildtype	
C_63	OMA19	OMA	Ovary (NS)	Macro	G12S	0·038	0·038	0·75	0·34	Wildtype	
C_64	DIE60	DIE	Uterosacral (L)	Macro	G12C	0·24	0·0020			Wildtype	
C_64	DIE60	DIE	Uterosacral (L)	Macro	G12D	0·24	0·0020			Wildtype	
C_64	DIE60	DIE	Uterosacral (L)	Macro	G12R	0·0048	0*			Wildtype	
C_64	DIE60	DIE	Uterosacral (L)	Macro	G12V	0·0060	0·096	0·019	1·4	Mutant	G12V
C_64	DIE60	DIE	Uterosacral (L)	Macro	G12A	0*	0*			Wildtype	
C_64	DIE60	DIE	Uterosacral (L)	Macro	G12S	0·23	0·016			Wildtype	
C_64	DIE61	DIE	Uterosacral (R)	LCM	G12C	0·010	0*			Wildtype	
C_64	DIE61	DIE	Uterosacral (R)	LCM	G12D	0·043	0·14	0·74	0·30	Wildtype	
C_64	DIE61	DIE	Uterosacral (R)	LCM	G12R	0·0016	0*			Wildtype	
C_64	DIE61	DIE	Uterosacral (R)	LCM	G12V	0·010	0*			Wildtype	
C_64	DIE61	DIE	Uterosacral (R)	LCM	G12A	0·0046	0*			Wildtype	
C_64	DIE61	DIE	Uterosacral (R)	LCM	G12S	0·085	0·018			Wildtype	
C_65	DIE92	DIE	Uterosacral (L)	LCM	G12C	0·010	0*			Wildtype	
C_65	DIE92	DIE	Uterosacral (L)	LCM	G12D	0·043	0·0035			Wildtype	
C_65	DIE92	DIE	Uterosacral (L)	LCM	G12R	0·0016	0·0069	0·020	0·084	Mutant	G12R
C_65	DIE92	DIE	Uterosacral (L)	LCM	G12V	0·010	0·003			Wildtype	
C_65	DIE92	DIE	Uterosacral (L)	LCM	G12A	0·0046	0*			Wildtype	
C_65	DIE92	DIE	Uterosacral (L)	LCM	G12S	0·085	0·024			Wildtype	
C_66	DIE21	DIE	Appendix	Macro	G12C	0·0020	0·0067	0·55	0·12	Wildtype	
C_66	DIE21	DIE	Appendix	Macro	G12D	0·0020	0·022	0·77	0·93	Mutant	G12D
C_66	DIE21	DIE	Appendix	Macro	G12R	0*	0*			Wildtype	
C_66	DIE21	DIE	Appendix	Macro	G12V	0·0060	0·054	0·27	1·4	Mutant	G12V
C_66	DIE21	DIE	Appendix	Macro	G12A	0·0060	0*			Wildtype	

Case ID	Sample ID	Endo type	Anatomical Location	Enrich. method	Variant tested	Multi. cut-off VAF (%)	Multi. VAF (%)	Indiv. cut-off VAF (%)	Indiv. VAF (%)	Result	*KRAS* Variant
C_66	DIE21	DIE	Appendix	Macro	G12S	0·0020	0·020	0·67	0·36	Wildtype	
C_66	OMA32	OMA	Ovary (B)	Macro	G12C	0·0058	0*			Wildtype	
C_66	OMA32	OMA	Ovary (B)	Macro	G12D	0·084	0·019			Wildtype	
C_66	OMA32	OMA	Ovary (B)	Macro	G12R	0*	0*			Wildtype	
C_66	OMA32	OMA	Ovary (B)	Macro	G12V	0·0036	0·0019			Wildtype	
C_66	OMA32	OMA	Ovary (B)	Macro	G12A	0·0022	0*			Wildtype	
C_66	OMA32	OMA	Ovary (B)	Macro	G12S	0·046	0·028			Wildtype	
C_67	OMA20	OMA	Ovary (L)	Macro	G12C	0·012	0·022	0·036	0·0023	Wildtype	
C_67	OMA20	OMA	Ovary (L)	Macro	G12D	0·017	0·027	0·71	0·31	Wildtype	
C_67	OMA20	OMA	Ovary (L)	Macro	G12R	0*	0*			Wildtype	
C_67	OMA20	OMA	Ovary (L)	Macro	G12V	0·010	0*			Wildtype	
C_67	OMA20	OMA	Ovary (L)	Macro	G12A	0*	0*			Wildtype	
C_67	OMA20	OMA	Ovary (L)	Macro	G12S	0·038	0·0056			Wildtype	
C_67	OMA43	OMA	Ovary (R)	Macro	G12C	0·0058	0*			Wildtype	
C_67	OMA43	OMA	Ovary (R)	Macro	G12D	0·084	0·036			Wildtype	
C_67	OMA43	OMA	Ovary (R)	Macro	G12R	0*	0*			Wildtype	
C_67	OMA43	OMA	Ovary (R)	Macro	G12V	0·0036	0·0045	0·0053	0*	Wildtype	
C_67	OMA43	OMA	Ovary (R)	Macro	G12A	0·0022	0*			Wildtype	
C_67	OMA43	OMA	Ovary (R)	Macro	G12S	0·046	0·011			Wildtype	
C_68	SUP28	SUP	Uterosacral (R)	Macro	G12C	0*	0*			Wildtype	
C_68	SUP28	SUP	Uterosacral (R)	Macro	G12D	0·019	0·013			Wildtype	
C_68	SUP28	SUP	Uterosacral (R)	Macro	G12R	0*	0*			Wildtype	
C_68	SUP28	SUP	Uterosacral (R)	Macro	G12V	0·0016	0·0084	0·017	0*	Wildtype	
C_68	SUP28	SUP	Uterosacral (R)	Macro	G12A	0·0016	0·0042	0·0044	0*	Wildtype	
C_68	SUP28	SUP	Uterosacral (R)	Macro	G12S	0·030	0·013			Wildtype	
C_68	SUP99	SUP	Uterosacral (L)	Macro	G12C	0·0058	0*			Wildtype	
C_68	SUP99	SUP	Uterosacral (L)	Macro	G12D	0·084	0·067	0·98	0·53	Wildtype	
C_68	SUP99	SUP	Uterosacral (L)	Macro	G12R	0*	0*			Wildtype	

Case ID	Sample ID	Endo type	Anatomical Location	Enrich. method	Variant tested	Multi. cut-off VAF (%)	Multi. VAF (%)	Indiv. cut-off VAF (%)	Indiv. VAF (%)	Result	*KRAS* Variant
C_68	SUP99	SUP	Uterosacral (L)	Macro	G12V	0·0036	0*			Wildtype	
C_68	SUP99	SUP	Uterosacral (L)	Macro	G12A	0·0022	0*			Wildtype	
C_68	SUP99	SUP	Uterosacral (L)	Macro	G12S	0·046	0·019			Wildtype	
C_69	SUP29	SUP	Fallopian tube (B)	Macro	G12C	0*	0*			Wildtype	
C_69	SUP29	SUP	Fallopian tube (B)	Macro	G12D	0·019	0·036	0·99	0·25	Wildtype	
C_69	SUP29	SUP	Fallopian tube (B)	Macro	G12R	0*	0*			Wildtype	
C_69	SUP29	SUP	Fallopian tube (B)	Macro	G12V	0·0016	0·0051	0·017	0*	Wildtype	
C_69	SUP29	SUP	Fallopian tube (B)	Macro	G12A	0·0016	0·015	0·0044	0*	Wildtype	
C_69	SUP29	SUP	Fallopian tube (B)	Macro	G12S	0·030	0·11	1·0	0·63	Wildtype	
C_70	DIE62	DIE	Uterosacral (R)	Macro	G12C	0·24	0·0080			Wildtype	
C_70	DIE62	DIE	Uterosacral (R)	Macro	G12D	0·24	0·0080			Wildtype	
C_70	DIE62	DIE	Uterosacral (R)	Macro	G12R	0·0048	0*			Wildtype	
C_70	DIE62	DIE	Uterosacral (R)	Macro	G12V	0·0060	0*			Wildtype	
C_70	DIE62	DIE	Uterosacral (R)	Macro	G12A	0*	0*			Wildtype	
C_70	DIE62	DIE	Uterosacral (R)	Macro	G12S	0·23	0·0040			Wildtype	
C_70	SUP100	SUP	Pelvic sidewall (L)	Macro	G12C	0·0058	0*			Wildtype	
C_70	SUP100	SUP	Pelvic sidewall (L)	Macro	G12D	0·084	0·058	0·98	0·47	Wildtype	
C_70	SUP100	SUP	Pelvic sidewall (L)	Macro	G12R	0*	0*			Wildtype	
C_70	SUP100	SUP	Pelvic sidewall (L)	Macro	G12V	0·0036	0·0025			Wildtype	
C_70	SUP100	SUP	Pelvic sidewall (L)	Macro	G12A	0·0022	0*			Wildtype	
C_70	SUP100	SUP	Pelvic sidewall (L)	Macro	G12S	0·046	0·020			Wildtype	
C_70	SUP30	SUP	Cul-de-sac (R)	Macro	G12C	0·0049	0*			Wildtype	
C_70	SUP30	SUP	Cul-de-sac (R)	Macro	G12D	0·012	0·32	0·62	0·74	Mutant	G12D
C_70	SUP30	SUP	Cul-de-sac (R)	Macro	G12R	0·0020	0*			Wildtype	
C_70	SUP30	SUP	Cul-de-sac (R)	Macro	G12V	0·0078	0·0050	0·0097	0*	Wildtype	
C_70	SUP30	SUP	Cul-de-sac (R)	Macro	G12A	0·014	0·0099			Wildtype	
C_70	SUP30	SUP	Cul-de-sac (R)	Macro	G12S	0·035	0·046	1·2	0·20	Wildtype	
C_71	SUP31	SUP	Fallopian tube (L)	Macro	G12C	0*	0*			Wildtype	

Case ID	Sample ID	Endo type	Anatomical Location	Enrich. method	Variant tested	Multi. cut-off VAF (%)	Multi. VAF (%)	Indiv. cut-off VAF (%)	Indiv. VAF (%)	Result	*KRAS* Variant
C_71	SUP31	SUP	Fallopian tube (L)	Macro	G12D	0·027	0·054	0·73	0·32	Wildtype	
C_71	SUP31	SUP	Fallopian tube (L)	Macro	G12R	0*	0*			Wildtype	
C_71	SUP31	SUP	Fallopian tube (L)	Macro	G12V	0*	0·0043	0·0065	0·0043	Wildtype	
C_71	SUP31	SUP	Fallopian tube (L)	Macro	G12A	0·0021	0*			Wildtype	
C_71	SUP31	SUP	Fallopian tube (L)	Macro	G12S	0·021	0·046	1·2	0·37	Wildtype	
C_72	OMA21	OMA	Ovary (R)	Macro	G12C	0·0041	0*			Wildtype	
C_72	OMA21	OMA	Ovary (R)	Macro	G12D	0·018	0*	0·62	0·22	Wildtype	
C_72	OMA21	OMA	Ovary (R)	Macro	G12R	0·0014	0*			Wildtype	
C_72	OMA21	OMA	Ovary (R)	Macro	G12V	0·00068	0·036	0·0060	0·028	Mutant	G12V
C_72	OMA21	OMA	Ovary (R)	Macro	G12A	0*	0*			Wildtype	
C_72	OMA21	OMA	Ovary (R)	Macro	G12S	0·033	0·061	0·44	0·13	Wildtype	
C_73	SUP49	SUP	Pelvic sidewall (R)	Macro	G12C	0*	0*			Wildtype	
C_73	SUP49	SUP	Pelvic sidewall (R)	Macro	G12D	0·027	0·061	0·73	1·2	Mutant	G12D
C_73	SUP49	SUP	Pelvic sidewall (R)	Macro	G12R	0*	0*			Wildtype	
C_73	SUP49	SUP	Pelvic sidewall (R)	Macro	G12V	0*	0·0079	0·0065	0·0023	Wildtype	
C_73	SUP49	SUP	Pelvic sidewall (R)	Macro	G12A	0·0021	0*			Wildtype	
C_73	SUP49	SUP	Pelvic sidewall (R)	Macro	G12S	0·021	0·0077			Wildtype	
C_74	DIE42	DIE	Periureteric (R)	Macro	G12C	0·24	0·032			Wildtype	
C_74	DIE42	DIE	Periureteric (R)	Macro	G12D	0·24	0·032			Wildtype	
C_74	DIE42	DIE	Periureteric (R)	Macro	G12R	0·0048	0*			Wildtype	
C_74	DIE42	DIE	Periureteric (R)	Macro	G12V	0·0060	0·21	0·019	0·26	Mutant	G12V
C_74	DIE42	DIE	Periureteric (R)	Macro	G12A	0*	0·016	0·0049	0*	Wildtype	
C_74	DIE42	DIE	Periureteric (R)	Macro	G12S	0·23	0·25	0·67	0·47	Wildtype	
C_74	DIE43	DIE	Periureteric (R)	Macro	G12C	0·24	0·010			Wildtype	
C_74	DIE43	DIE	Periureteric (R)	Macro	G12D	0·24	0·010			Wildtype	
C_74	DIE43	DIE	Periureteric (R)	Macro	G12R	0·0048	0*			Wildtype	
C_74	DIE43	DIE	Periureteric (R)	Macro	G12V	0·0060	0*			Wildtype	
C_74	DIE43	DIE	Periureteric (R)	Macro	G12A	0*	0*			Wildtype	

Case ID	Sample ID	Endo type	Anatomical Location	Enrich. method	Variant tested	Multi. cut-off VAF (%)	Multi. VAF (%)	Indiv. cut-off VAF (%)	Indiv. VAF (%)	Result	*KRAS* Variant
C_74	DIE43	DIE	Periureteric (R)	Macro	G12S	0·23	0·015			Wildtype	
C_75	DIE44	DIE	Uterosacral (L)	Macro	G12C	0·24	0·0043			Wildtype	
C_75	DIE44	DIE	Uterosacral (L)	Macro	G12D	0·24	0·0043			Wildtype	
C_75	DIE44	DIE	Uterosacral (L)	Macro	G12R	0·0048	0*			Wildtype	
C_75	DIE44	DIE	Uterosacral (L)	Macro	G12V	0·0060	0·0022			Wildtype	
C_75	DIE44	DIE	Uterosacral (L)	Macro	G12A	0*	0*			Wildtype	
C_75	DIE44	DIE	Uterosacral (L)	Macro	G12S	0·23	0·011			Wildtype	
C_75	DIE45	DIE	Uterosacral (R)	Macro	G12C	0·24	0·038			Wildtype	
C_75	DIE45	DIE	Uterosacral (R)	Macro	G12D	0·24	0·038	0·35	0·27	Wildtype	
C_75	DIE45	DIE	Uterosacral (R)	Macro	G12R	0·0048	0*			Wildtype	
C_75	DIE45	DIE	Uterosacral (R)	Macro	G12V	0·0060	0*			Wildtype	
C_75	DIE45	DIE	Uterosacral (R)	Macro	G12A	0*	0*			Wildtype	
C_75	DIE45	DIE	Uterosacral (R)	Macro	G12S	0·23	0·024	0·67	0·18	Wildtype	
C_75	OMA22	OMA	Endometrium (L)	Macro	G12C	0·012	0*			Wildtype	
C_75	OMA22	OMA	Endometrium (L)	Macro	G12D	0·017	0*			Wildtype	
C_75	OMA22	OMA	Endometrium (L)	Macro	G12R	0*	0*			Wildtype	
C_75	OMA22	OMA	Endometrium (L)	Macro	G12V	0·0097	0*			Wildtype	
C_75	OMA22	OMA	Endometrium (L)	Macro	G12A	0*	0*			Wildtype	
C_75	OMA22	OMA	Endometrium (L)	Macro	G12S	0·038	0·038	0·75	0·088	Wildtype	
C_75	SUP32	SUP	Cul-de-sac	Macro	G12C	0·0049	0*			Wildtype	
C_75	SUP32	SUP	Cul-de-sac	Macro	G12D	0·012	0·021	0·62	0·13	Wildtype	
C_75	SUP32	SUP	Cul-de-sac	Macro	G12R	0·0020	0*			Wildtype	
C_75	SUP32	SUP	Cul-de-sac	Macro	G12V	0·0078	0·0035	0·0097	0·0040	Wildtype	
C_75	SUP32	SUP	Cul-de-sac	Macro	G12A	0·014	0·025	0*	0*	Wildtype	
C_75	SUP32	SUP	Cul-de-sac	Macro	G12S	0·035	0·053	1·2	0·28	Wildtype	
C_76	SUP33	SUP	Pelvic sidewall (L)	Macro	G12C	0·0049	0·040	0·025	0·081	Mutant	G12C
C_76	SUP33	SUP	Pelvic sidewall (L)	Macro	G12D	0·012	0·22	0·62	0·54	Wildtype	
C_76	SUP33	SUP	Pelvic sidewall (L)	Macro	G12R	0·0020	0*			Wildtype	

Case ID	Sample ID	Endo type	Anatomical Location	Enrich. method	Variant tested	Multi. cut-off VAF (%)	Multi. VAF (%)	Indiv. cut-off VAF (%)	Indiv. VAF (%)	Result	*KRAS* Variant
C_76	SUP33	SUP	Pelvic sidewall (L)	Macro	G12V	0·0078	0*			Wildtype	
C_76	SUP33	SUP	Pelvic sidewall (L)	Macro	G12A	0·014	0·11	0*	0*	Wildtype	
C_76	SUP33	SUP	Pelvic sidewall (L)	Macro	G12S	0·035	0·24	1·2	0·51	Wildtype	
C_77	SUP50	SUP	Ovary (NS)	Macro	G12C	0*	0*			Wildtype	
C_77	SUP50	SUP	Ovary (NS)	Macro	G12D	0·027	0·0082			Wildtype	
C_77	SUP50	SUP	Ovary (NS)	Macro	G12R	0*	0*			Wildtype	
C_77	SUP50	SUP	Ovary (NS)	Macro	G12V	0*	0·0043	0·0065	0·015	Mutant	G12V
C_77	SUP50	SUP	Ovary (NS)	Macro	G12A	0·0021	0*			Wildtype	
C_77	SUP50	SUP	Ovary (NS)	Macro	G12S	0·021	0·0021			Wildtype	
C_78	SUP51	SUP	Uterosacral (L)	Macro	G12C	0*	0*			Wildtype	
C_78	SUP51	SUP	Uterosacral (L)	Macro	G12D	0·027	0·0038			Wildtype	
C_78	SUP51	SUP	Uterosacral (L)	Macro	G12R	0*	0*			Wildtype	
C_78	SUP51	SUP	Uterosacral (L)	Macro	G12V	0*	0·0020	0·0065	0·0045	Wildtype	
C_78	SUP51	SUP	Uterosacral (L)	Macro	G12A	0·0021	0·0019			Wildtype	
C_78	SUP51	SUP	Uterosacral (L)	Macro	G12S	0·021	0·0020			Wildtype	
C_78	SUP101	SUP	Uterosacral (R)	Macro	G12C	0·0058	0*			Wildtype	
C_78	SUP101	SUP	Uterosacral (R)	Macro	G12D	0·084	0·040			Wildtype	
C_78	SUP101	SUP	Uterosacral (R)	Macro	G12R	0*	0*			Wildtype	
C_78	SUP101	SUP	Uterosacral (R)	Macro	G12V	0·0036	0*			Wildtype	
C_78	SUP101	SUP	Uterosacral (R)	Macro	G12A	0·0022	0*			Wildtype	
C_78	SUP101	SUP	Uterosacral (R)	Macro	G12S	0·046	0·0057			Wildtype	
C_79	DIE49	DIE	Cul-de-sac (A)	Macro	G12C	0·24	0·057			Wildtype	
C_79	DIE49	DIE	Cul-de-sac (A)	Macro	G12D	0·24	0·057			Wildtype	
C_79	DIE49	DIE	Cul-de-sac (A)	Macro	G12R	0·0048	0*			Wildtype	
C_79	DIE49	DIE	Cul-de-sac (A)	Macro	G12V	0·0060	0·019	0·019	0·018	Wildtype	
C_79	DIE49	DIE	Cul-de-sac (A)	Macro	G12A	0*	0*			Wildtype	
C_79	DIE49	DIE	Cul-de-sac (A)	Macro	G12S	0·23	0·038			Wildtype	
C_79	OMA23	OMA	Ovary (L)	Macro	G12C	0·012	0·028	0·036	0*	Wildtype	

Case ID	Sample ID	Endo type	Anatomical Location	Enrich. method	Variant tested	Multi. cut-off VAF (%)	Multi. VAF (%)	Indiv. cut-off VAF (%)	Indiv. VAF (%)	Result	*KRAS* Variant
C_79	OMA23	OMA	Ovary (L)	Macro	G12D	0·017	0·028	0·71	0·19	Wildtype	
C_79	OMA23	OMA	Ovary (L)	Macro	G12R	0*	0*			Wildtype	
C_79	OMA23	OMA	Ovary (L)	Macro	G12V	0·0097	0·0056	0*	0*	Wildtype	
C_79	OMA23	OMA	Ovary (L)	Macro	G12A	0*	0*			Wildtype	
C_79	OMA23	OMA	Ovary (L)	Macro	G12S	0·038	0·034			Wildtype	
C_79	SUP52	SUP	Cul-de-sac (A)	Macro	G12C	0*	0*			Wildtype	
C_79	SUP52	SUP	Cul-de-sac (A)	Macro	G12D	0·027	0·0039			Wildtype	
C_79	SUP52	SUP	Cul-de-sac (A)	Macro	G12R	0*	0*			Wildtype	
C_79	SUP52	SUP	Cul-de-sac (A)	Macro	G12V	0*	0·0020	0·0065	0·0022	Wildtype	
C_79	SUP52	SUP	Cul-de-sac (A)	Macro	G12A	0·0021	0*			Wildtype	
C_79	SUP52	SUP	Cul-de-sac (A)	Macro	G12S	0·021	0·016			Wildtype	
C_80	DIE29	DIE	Uterosacral (R)	Macro	G12C	0·24	0·015			Wildtype	
C_80	DIE29	DIE	Uterosacral (R)	Macro	G12D	0·24	0·015			Wildtype	
C_80	DIE29	DIE	Uterosacral (R)	Macro	G12R	0·0048	0*			Wildtype	
C_80	DIE29	DIE	Uterosacral (R)	Macro	G12V	0·0060	0·0050	0·019	0·14	Mutant	G12V
C_80	DIE29	DIE	Uterosacral (R)	Macro	G12A	0*	0*			Wildtype	
C_80	DIE29	DIE	Uterosacral (R)	Macro	G12S	0·23	0·030	0·67	0·53	Wildtype	
C_80	DIE30	DIE	Pelvic sidewall (R)	Macro	G12C	0·24	0·0037			Wildtype	
C_80	DIE30	DIE	Pelvic sidewall (R)	Macro	G12D	0·24	0·0037			Wildtype	
C_80	DIE30	DIE	Pelvic sidewall (R)	Macro	G12R	0·0048	0*			Wildtype	
C_80	DIE30	DIE	Pelvic sidewall (R)	Macro	G12V	0·0060	0*			Wildtype	
C_80	DIE30	DIE	Pelvic sidewall (R)	Macro	G12A	0*	0*			Wildtype	
C_80	DIE30	DIE	Pelvic sidewall (R)	Macro	G12S	0·23	0*			Wildtype	
C_80	SUP53	SUP	Cul-de-sac (L)	Macro	G12C	0·0049	0*			Wildtype	
C_80	SUP53	SUP	Cul-de-sac (L)	Macro	G12D	0·012	0*			Wildtype	
C_80	SUP53	SUP	Cul-de-sac (L)	Macro	G12R	0·0020	0*			Wildtype	
C_80	SUP53	SUP	Cul-de-sac (L)	Macro	G12V	0·0078	0·018	0·0097	0·0077	Wildtype	
C_80	SUP53	SUP	Cul-de-sac (L)	Macro	G12A	0·014	0·14	0*	0·64	Mutant	G12A

Case ID	Sample ID	Endo type	Anatomical Location	Enrich. method	Variant tested	Multi. cut-off VAF (%)	Multi. VAF (%)	Indiv. cut-off VAF (%)	Indiv. VAF (%)	Result	*KRAS* Variant
C_80	SUP53	SUP	Cul-de-sac (L)	Macro	G12S	0·035	0·025			Wildtype	
C_80	SUP102	SUP	Cul-de-sac (R)	Macro	G12C	0·0058	0*			Wildtype	
C_80	SUP102	SUP	Cul-de-sac (R)	Macro	G12D	0·084	0·0058			Wildtype	
C_80	SUP102	SUP	Cul-de-sac (R)	Macro	G12R	0*	0*			Wildtype	
C_80	SUP102	SUP	Cul-de-sac (R)	Macro	G12V	0·0036	0*			Wildtype	
C_80	SUP102	SUP	Cul-de-sac (R)	Macro	G12A	0·0022	0*			Wildtype	
C_80	SUP102	SUP	Cul-de-sac (R)	Macro	G12S	0·046	0·0019			Wildtype	
C_81	SUP34	SUP	Fallopian tube (L)	Macro	G12C	0·0049	0·012	0·025	0·014	Wildtype	
C_81	SUP34	SUP	Fallopian tube (L)	Macro	G12D	0·012	0·062	0·62	0·57	Wildtype	
C_81	SUP34	SUP	Fallopian tube (L)	Macro	G12R	0·0020	0*			Wildtype	
C_81	SUP34	SUP	Fallopian tube (L)	Macro	G12V	0·0078	0·0074	0·0097	0·0082	Wildtype	
C_81	SUP34	SUP	Fallopian tube (L)	Macro	G12A	0·014	0·010			Wildtype	
C_81	SUP34	SUP	Fallopian tube (L)	Macro	G12S	0·035	0·020			Wildtype	
C_81	SUP103	SUP	Ovary (L)	Macro	G12C	0·0058	0*			Wildtype	
C_81	SUP103	SUP	Ovary (L)	Macro	G12D	0·084	0·018			Wildtype	
C_81	SUP103	SUP	Ovary (L)	Macro	G12R	0*	0*			Wildtype	
C_81	SUP103	SUP	Ovary (L)	Macro	G12V	0·0036	0·0050	0·0053	0*	Wildtype	
C_81	SUP103	SUP	Ovary (L)	Macro	G12A	0·0022	0*			Wildtype	
C_81	SUP103	SUP	Ovary (L)	Macro	G12S	0·046	0·015			Wildtype	
C_82	DIE31	DIE	Rectum	Macro	G12C	0·24	0·059			Wildtype	
C_82	DIE31	DIE	Rectum	Macro	G12D	0·24	0·059			Wildtype	
C_82	DIE31	DIE	Rectum	Macro	G12R	0·0048	0*			Wildtype	
C_82	DIE31	DIE	Rectum	Macro	G12V	0·0060	0*			Wildtype	
C_82	DIE31	DIE	Rectum	Macro	G12A	0*	0*			Wildtype	
C_82	DIE31	DIE	Rectum	Macro	G12S	0·23	0·030			Wildtype	
C_82	DIE32	DIE	Appendix	Macro	G12C	0·24	0·088			Wildtype	
C_82	DIE32	DIE	Appendix	Macro	G12D	0·24	0·088			Wildtype	
C_82	DIE32	DIE	Appendix	Macro	G12R	0·0048	0*			Wildtype	

Case ID	Sample ID	Endo type	Anatomical Location	Enrich. method	Variant tested	Multi. cut-off VAF (%)	Multi. VAF (%)	Indiv. cut-off VAF (%)	Indiv. VAF (%)	Result	*KRAS* Variant
C_82	DIE32	DIE	Appendix	Macro	G12V	0·0060	0*			Wildtype	
C_82	DIE32	DIE	Appendix	Macro	G12A	0*	0*			Wildtype	
C_82	DIE32	DIE	Appendix	Macro	G12S	0·23	0·036			Wildtype	
C_82	DIE33	DIE	Fallopian tube (R)	Macro	G12C	0·24	0·032			Wildtype	
C_82	DIE33	DIE	Fallopian tube (R)	Macro	G12D	0·24	0·032			Wildtype	
C_82	DIE33	DIE	Fallopian tube (R)	Macro	G12R	0·0048	0*			Wildtype	
C_82	DIE33	DIE	Fallopian tube (R)	Macro	G12V	0·0060	0·019	0·019	0*	Wildtype	
C_82	DIE33	DIE	Fallopian tube (R)	Macro	G12A	0*	0*			Wildtype	
C_82	DIE33	DIE	Fallopian tube (R)	Macro	G12S	0·23	0·097	0·67	0·35	Wildtype	
C_82	DIE34	DIE	Vagina	Macro	G12C	0·24	0·0048			Wildtype	
C_82	DIE34	DIE	Vagina	Macro	G12D	0·24	0·0048			Wildtype	
C_82	DIE34	DIE	Vagina	Macro	G12R	0·0048	0*			Wildtype	
C_82	DIE34	DIE	Vagina	Macro	G12V	0·0060	0·0048			Wildtype	
C_82	DIE34	DIE	Vagina	Macro	G12A	0*	0*			Wildtype	
C_82	DIE34	DIE	Vagina	Macro	G12S	0·23	0·029			Wildtype	
C_82	DIE35	DIE	Vagina	Macro	G12C	0·29	0·076	0·030	0*	Wildtype	
C_82	DIE35	DIE	Vagina	Macro	G12D	0·24	0·091	1·0	0·186	Wildtype	
C_82	DIE35	DIE	Vagina	Macro	G12R	0·0048	0*	0·0020	0*	Wildtype	
C_82	DIE35	DIE	Vagina	Macro	G12V	failed - not enough droplets	failed - not enough droplets	0·010	0*	Wildtype	
C_82	DIE35	DIE	Vagina	Macro	G12A	0*	0*	0·0049	0*	Wildtype	
C_82	DIE35	DIE	Vagina	Macro	G12S	0·23	0·030	0·73	0·31	Wildtype	
C_82	DIE36	DIE	Cervix Uteri (P)	Macro	G12C	0·010	0*			Wildtype	
C_82	DIE36	DIE	Cervix Uteri (P)	Macro	G12D	0·043	0·0069			Wildtype	
C_82	DIE36	DIE	Cervix Uteri (P)	Macro	G12R	0·0016	0*			Wildtype	
C_82	DIE36	DIE	Cervix Uteri (P)	Macro	G12V	0·010	0·0035			Wildtype	
C_82	DIE36	DIE	Cervix Uteri (P)	Macro	G12A	0·0046	0·0035			Wildtype	
C_82	DIE36	DIE	Cervix Uteri (P)	Macro	G12S	0·085	0·028	1·2	0·20	Wildtype	

Case ID	Sample ID	Endo type	Anatomical Location	Enrich. method	Variant tested	Multi. cut-off VAF (%)	Multi. VAF (%)	Indiv. cut-off VAF (%)	Indiv. VAF (%)	Result	*KRAS* Variant
C_82	OMA24	OMA	Ovary (R)	Macro	G12C	0*	0*			Wildtype	
C_82	OMA24	OMA	Ovary (R)	Macro	G12D	0·027	0·0038			Wildtype	
C_82	OMA24	OMA	Ovary (R)	Macro	G12R	0*	0*			Wildtype	
C_82	OMA24	OMA	Ovary (R)	Macro	G12V	0*	0*	0·0065	0·0034	Wildtype	
C_82	OMA24	OMA	Ovary (R)	Macro	G12A	0·0021	0·0037	0*	0·065	Mutant	G12A
C_82	OMA24	OMA	Ovary (R)	Macro	G12S	0·021	0·0060			Wildtype	
C_83	OMA25	OMA	Ovary (R)	Macro	G12C	0·012	0·18	0·036	0*	Wildtype	
C_83	OMA25	OMA	Ovary (R)	Macro	G12D	0·017	0·22	0·71	0·74	Mutant	G12D
C_83	OMA25	OMA	Ovary (R)	Macro	G12R	0*	0*			Wildtype	
C_83	OMA25	OMA	Ovary (R)	Macro	G12V	0·0097	0·028	0*	0·081	Mutant	G12V
C_83	OMA25	OMA	Ovary (R)	Macro	G12A	0*	0*			Wildtype	
C_83	OMA25	OMA	Ovary (R)	Macro	G12S	0·038	0·029			Wildtype	
C_83	OMA44	OMA	Ovary (L)	Macro	G12C	0·0058	0*			Wildtype	
C_83	OMA44	OMA	Ovary (L)	Macro	G12D	0·084	0·0089			Wildtype	
C_83	OMA44	OMA	Ovary (L)	Macro	G12R	0*	0·0022	0·0020	0*	Wildtype	
C_83	OMA44	OMA	Ovary (L)	Macro	G12V	0·0036	0·0044	0·0053	0·0046	Wildtype	
C_83	OMA44	OMA	Ovary (L)	Macro	G12A	0·0022	0*			Wildtype	
C_83	OMA44	OMA	Ovary (L)	Macro	G12S	0·046	0·033			Wildtype	
C_84	SUP35	SUP	Ovary (L)	Macro	G12C	0·0049	0*			Wildtype	
C_84	SUP35	SUP	Ovary (L)	Macro	G12D	0·012	0·015	0·62	0·21	Wildtype	
C_84	SUP35	SUP	Ovary (L)	Macro	G12R	0·0020	0*			Wildtype	
C_84	SUP35	SUP	Ovary (L)	Macro	G12V	0·0078	0·0048	0·0097	0*	Wildtype	
C_84	SUP35	SUP	Ovary (L)	Macro	G12A	0·014	0*			Wildtype	
C_84	SUP35	SUP	Ovary (L)	Macro	G12S	0·035	0·0049			Wildtype	
C_85	DIE63	DIE	Uterosacral (L)	Macro	G12C	0·24	0·055			Wildtype	
C_85	DIE63	DIE	Uterosacral (L)	Macro	G12D	0·24	0·055			Wildtype	
C_85	DIE63	DIE	Uterosacral (L)	Macro	G12R	0·0048	0*			Wildtype	
C_85	DIE63	DIE	Uterosacral (L)	Macro	G12V	0·0060	0·014	0·019	0·046	Mutant	G12V

Case ID	Sample ID	Endo type	Anatomical Location	Enrich. method	Variant tested	Multi. cut-off VAF (%)	Multi. VAF (%)	Indiv. cut-off VAF (%)	Indiv. VAF (%)	Result	*KRAS* Variant
C_85	DIE63	DIE	Uterosacral (L)	Macro	G12A	0*	0*			Wildtype	
C_85	DIE63	DIE	Uterosacral (L)	Macro	G12S	0·23	0·12	0·67	0·56	Wildtype	
C_86	SUP54	SUP	Uterosacral (R)	Macro	G12C	0·0049	0*			Wildtype	
C_86	SUP54	SUP	Uterosacral (R)	Macro	G12D	0·012	0·044	0·62	0·066	Wildtype	
C_86	SUP54	SUP	Uterosacral (R)	Macro	G12R	0·0020	0*			Wildtype	
C_86	SUP54	SUP	Uterosacral (R)	Macro	G12V	0·0078	0·0088	0·0060	0*	Wildtype	
C_86	SUP54	SUP	Uterosacral (R)	Macro	G12A	0·014	0·044	0*	0*	Wildtype	
C_86	SUP54	SUP	Uterosacral (R)	Macro	G12S	0·035	0·19	1·2	0·40	Wildtype	
C_87	SUP36	SUP	Pelvic sidewall (L)	Macro	G12C	0·0041	0*			Wildtype	
C_87	SUP36	SUP	Pelvic sidewall (L)	Macro	G12D	0·018	0·021	0·73	0·10	Wildtype	
C_87	SUP36	SUP	Pelvic sidewall (L)	Macro	G12R	0·0014	0*			Wildtype	
C_87	SUP36	SUP	Pelvic sidewall (L)	Macro	G12V	0·00070	0·042	0·0060	0·17	Mutant	G12V
C_87	SUP36	SUP	Pelvic sidewall (L)	Macro	G12A	0*	0*			Wildtype	
C_87	SUP36	SUP	Pelvic sidewall (L)	Macro	G12S	0·033	0·057	0·44	0·28	Wildtype	
C_88	OMA26	OMA	Ovary (L)	Macro	G12C	0·012	0·030	0·036	0*	Wildtype	
C_88	OMA26	OMA	Ovary (L)	Macro	G12D	0·017	0·033	0·71	0·24	Wildtype	
C_88	OMA26	OMA	Ovary (L)	Macro	G12R	0*	0*			Wildtype	
C_88	OMA26	OMA	Ovary (L)	Macro	G12V	0·0097	0·0037			Wildtype	
C_88	OMA26	OMA	Ovary (L)	Macro	G12A	0*	0*			Wildtype	
C_88	OMA26	OMA	Ovary (L)	Macro	G12S	0·038	0·019			Wildtype	
C_88	SUP37	SUP	Fallopian tube (L)	Macro	G12C	0·0049	0·023	0·0048	0*	Wildtype	
C_88	SUP37	SUP	Fallopian tube (L)	Macro	G12D	0·012	0·29	0·62	1·1	Mutant	G12D
C_88	SUP37	SUP	Fallopian tube (L)	Macro	G12R	0·0020	0*			Wildtype	
C_88	SUP37	SUP	Fallopian tube (L)	Macro	G12V	0·0078	0·0046	0·0097	0·0095	Wildtype	
C_88	SUP37	SUP	Fallopian tube (L)	Macro	G12A	0·014	0·023	0*	0*	Wildtype	
C_88	SUP37	SUP	Fallopian tube (L)	Macro	G12S	0·035	0·074	1·2	0·33	Wildtype	
C_88	SUP104	SUP	Pelvic sidewall (L)	Macro	G12C	0·0058	0*			Wildtype	
C_88	SUP104	SUP	Pelvic sidewall (L)	Macro	G12D	0·084	0·010			Wildtype	

Case ID	Sample ID	Endo type	Anatomical Location	Enrich. method	Variant tested	Multi. cut-off VAF (%)	Multi. VAF (%)	Indiv. cut-off VAF (%)	Indiv. VAF (%)	Result	*KRAS* Variant
C_88	SUP104	SUP	Pelvic sidewall (L)	Macro	G12R	0*	0*			Wildtype	
C_88	SUP104	SUP	Pelvic sidewall (L)	Macro	G12V	0·0036	0*			Wildtype	
C_88	SUP104	SUP	Pelvic sidewall (L)	Macro	G12A	0·0022	0*			Wildtype	
C_88	SUP104	SUP	Pelvic sidewall (L)	Macro	G12S	0·046	0·0068			Wildtype	
C_89	DIE66	DIE	Uterosacral (L)	Macro	G12C	0·24	0·13			Wildtype	
C_89	DIE66	DIE	Uterosacral (L)	Macro	G12D	0·24	0·13	0·35	0·55	Mutant	G12D
C_89	DIE66	DIE	Uterosacral (L)	Macro	G12R	0·0048	0*			Wildtype	
C_89	DIE66	DIE	Uterosacral (L)	Macro	G12V	0·0060	0·0040			Wildtype	
C_89	DIE66	DIE	Uterosacral (L)	Macro	G12A	0*	0*			Wildtype	
C_89	DIE66	DIE	Uterosacral (L)	Macro	G12S	0·23	0·074	0·67	0·33	Wildtype	
C_89	DIE67	DIE	Uterosacral (L)	Macro	G12C	0·24	0·0028			Wildtype	
C_89	DIE67	DIE	Uterosacral (L)	Macro	G12D	0·24	0·047	0·35	0·79	Mutant	G12D
C_89	DIE67	DIE	Uterosacral (L)	Macro	G12R	0·0048	0*			Wildtype	
C_89	DIE67	DIE	Uterosacral (L)	Macro	G12V	0·0060	0·011	0·019	0·43	Mutant	G12V
C_89	DIE67	DIE	Uterosacral (L)	Macro	G12A	0*	0*			Wildtype	
C_89	DIE67	DIE	Uterosacral (L)	Macro	G12S	0·23	0·0083			Wildtype	
C_90	DIE53	DIE	Uterosacral (L)	Macro	G12C	0·34	0·40	0·12	0·33	Mutant	G12C
C_90	DIE53	DIE	Uterosacral (L)	Macro	G12D	0·24	0·23	0·35	0·65	Mutant	G12D
C_90	DIE53	DIE	Uterosacral (L)	Macro	G12R	0·0048	0*	0·0080	0*	Wildtype	
C_90	DIE53	DIE	Uterosacral (L)	Macro	G12V	0·0060	0*	0·019	0·0086	Wildtype	
C_90	DIE53	DIE	Uterosacral (L)	Macro	G12A	0*	0*	0*	0*	Wildtype	
C_90	DIE53	DIE	Uterosacral (L)	Macro	G12S	0·23	0·13	0·67	0·47	Wildtype	
C_90	DIE54	DIE	Uterosacral (L)	Macro	G12C	0·24	0·049			Wildtype	
C_90	DIE54	DIE	Uterosacral (L)	Macro	G12D	0·24	0·049	0·35	0·37	Mutant	G12D
C_90	DIE54	DIE	Uterosacral (L)	Macro	G12R	0·0048	0*			Wildtype	
C_90	DIE54	DIE	Uterosacral (L)	Macro	G12V	0·0060	0·0027			Wildtype	
C_90	DIE54	DIE	Uterosacral (L)	Macro	G12A	0*	0*			Wildtype	
C_90	DIE54	DIE	Uterosacral (L)	Macro	G12S	0·23	0·019			Wildtype	

Case ID	Sample ID	Endo type	Anatomical Location	Enrich. method	Variant tested	Multi. cut-off VAF (%)	Multi. VAF (%)	Indiv. cut-off VAF (%)	Indiv. VAF (%)	Result	*KRAS* Variant
C_90	DIE55	DIE	Uterosacral (R)	Macro	G12C	0·24	0·20			Wildtype	
C_90	DIE55	DIE	Uterosacral (R)	Macro	G12D	0·24	0·29	0·35	0·59	Mutant	G12D
C_90	DIE55	DIE	Uterosacral (R)	Macro	G12R	0·0048	0*			Wildtype	
C_90	DIE55	DIE	Uterosacral (R)	Macro	G12V	0·0060	0·039	0·019	0·16	Mutant	G12V
C_90	DIE55	DIE	Uterosacral (R)	Macro	G12A	0*	0*			Wildtype	
C_90	DIE55	DIE	Uterosacral (R)	Macro	G12S	0·23	0·031			Wildtype	
C_90	DIE56	DIE	Uterosacral (R)	Macro	G12C	0·24	0·0019			Wildtype	
C_90	DIE56	DIE	Uterosacral (R)	Macro	G12D	0·24	0·0019			Wildtype	
C_90	DIE56	DIE	Uterosacral (R)	Macro	G12R	0·0048	0*			Wildtype	
C_90	DIE56	DIE	Uterosacral (R)	Macro	G12V	0·0060	0*			Wildtype	
C_90	DIE56	DIE	Uterosacral (R)	Macro	G12A	0*	0*			Wildtype	
C_90	DIE56	DIE	Uterosacral (R)	Macro	G12S	0·23	0·0095			Wildtype	
C_90	SUP59	SUP	Pelvic sidewall (R)	Macro	G12C	0·0049	0·012	0·0048	0*	Wildtype	
C_90	SUP59	SUP	Pelvic sidewall (R)	Macro	G12D	0·012	0·082	0·62	0·24	Wildtype	
C_90	SUP59	SUP	Pelvic sidewall (R)	Macro	G12R	0·0020	0*			Wildtype	
C_90	SUP59	SUP	Pelvic sidewall (R)	Macro	G12V	0·0078	0*			Wildtype	
C_90	SUP59	SUP	Pelvic sidewall (R)	Macro	G12A	0·014	0·012			Wildtype	
C_90	SUP59	SUP	Pelvic sidewall (R)	Macro	G12S	0·035	0·059	1·2	0·17	Wildtype	
C_91	SUP60	SUP	Pelvic sidewall (L)	Macro	G12C	0·0049	0·0033			Wildtype	
C_91	SUP60	SUP	Pelvic sidewall (L)	Macro	G12D	0·012	0·013	0·62	0·13	Wildtype	
C_91	SUP60	SUP	Pelvic sidewall (L)	Macro	G12R	0·0020	0*			Wildtype	
C_91	SUP60	SUP	Pelvic sidewall (L)	Macro	G12V	0·0078	0·0066	0·0097	0*	Wildtype	
C_91	SUP60	SUP	Pelvic sidewall (L)	Macro	G12A	0·014	0·017	0*	0*	Wildtype	
C_91	SUP60	SUP	Pelvic sidewall (L)	Macro	G12S	0·035	0·033	0·44	0·18	Wildtype	
C_92	SUP61	SUP	Pelvic sidewall (R)	Macro	G12C	0·0049	0·030	0·0048	0*	Wildtype	
C_92	SUP61	SUP	Pelvic sidewall (R)	Macro	G12D	0·012	0·12	0·62	0·52	Wildtype	
C_92	SUP61	SUP	Pelvic sidewall (R)	Macro	G12R	0·0020	0*			Wildtype	
C_92	SUP61	SUP	Pelvic sidewall (R)	Macro	G12V	0·0078	0·0043	0·0060	0*	Mutant	G12V

Case ID	Sample ID	Endo type	Anatomical Location	Enrich. method	Variant tested	Multi. cut-off VAF (%)	Multi. VAF (%)	Indiv. cut-off VAF (%)	Indiv. VAF (%)	Result	*KRAS* Variant
C_92	SUP61	SUP	Pelvic sidewall (R)	Macro	G12A	0·014	0·035	0*	0*	Wildtype	
C_92	SUP61	SUP	Pelvic sidewall (R)	Macro	G12S	0·035	0·065	1·2	0·37	Wildtype	
C_93	OMA33	OMA	Ovary (B)	Macro	G12C	failed - not enough droplets	failed - not enough droplets	0·0039	0·0013	Wildtype	
C_93	OMA33	OMA	Ovary (B)	Macro	G12D	failed - not enough droplets	failed - not enough droplets	1·5	0·28	Wildtype	
C_93	OMA33	OMA	Ovary (B)	Macro	G12R	failed - not enough droplets	failed - not enough droplets	0·0020	0*	Wildtype	
C_93	OMA33	OMA	Ovary (B)	Macro	G12V	failed - not enough droplets	failed - not enough droplets	0·0039	0*	Wildtype	
C_93	OMA33	OMA	Ovary (B)	Macro	G12A	failed - not enough droplets	failed - not enough droplets	0·0022	0·0013	Wildtype	
C_93	OMA33	OMA	Ovary (B)	Macro	G12S	failed - not enough droplets	failed - not enough droplets	0·73	0·12	Wildtype	
C_94	DIE57	DIE	Uterosacral (L)	Macro	G12C	0·24	0*			Wildtype	
C_94	DIE57	DIE	Uterosacral (L)	Macro	G12D	0·24	0*			Wildtype	
C_94	DIE57	DIE	Uterosacral (L)	Macro	G12R	0·0048	0·030	0·0080	0·12	Mutant	G12R
C_94	DIE57	DIE	Uterosacral (L)	Macro	G12V	0·0060	0*			Wildtype	
C_94	DIE57	DIE	Uterosacral (L)	Macro	G12A	0*	0*			Wildtype	
C_94	DIE57	DIE	Uterosacral (L)	Macro	G12S	0·23	0·068	0·67	0·40	Wildtype	
C_94	DIE58	DIE	Uterosacral (R)	Macro	G12C	0·24	0·24			Wildtype	
C_94	DIE58	DIE	Uterosacral (R)	Macro	G12D	0·24	0·24	0·35	0·61	Mutant	G12D
C_94	DIE58	DIE	Uterosacral (R)	Macro	G12R	0·0048	0*			Wildtype	
C_94	DIE58	DIE	Uterosacral (R)	Macro	G12V	0·0015	0·016	0·010	0*	Wildtype	
C_94	DIE58	DIE	Uterosacral (R)	Macro	G12A	0*	0*			Wildtype	

Case ID	Sample ID	Endo type	Anatomical Location	Enrich. method	Variant tested	Multi. cut-off VAF (%)	Multi. VAF (%)	Indiv. cut-off VAF (%)	Indiv. VAF (%)	Result	*KRAS* Variant
C_94	DIE58	DIE	Uterosacral (R)	Macro	G12S	0·23	0*			Wildtype	
C_95	SUP105	SUP	Uterosacral (R)	Macro	G12C	0·0058	0*			Wildtype	
C_95	SUP105	SUP	Uterosacral (R)	Macro	G12D	0·084	0·0098			Wildtype	
C_95	SUP105	SUP	Uterosacral (R)	Macro	G12R	0*	0*			Wildtype	
C_95	SUP105	SUP	Uterosacral (R)	Macro	G12V	0·0036	0·0016			Wildtype	
C_95	SUP105	SUP	Uterosacral (R)	Macro	G12A	0·0022	0*			Wildtype	
C_95	SUP105	SUP	Uterosacral (R)	Macro	G12S	0·046	0·0081			Wildtype	
C_96	SUP62	SUP	Pelvic sidewall (L)	Macro	G12C	0·0049	0·036	0·0048	0*	Wildtype	
C_96	SUP62	SUP	Pelvic sidewall (L)	Macro	G12D	0·012	0·11	0·62	0·20	Wildtype	
C_96	SUP62	SUP	Pelvic sidewall (L)	Macro	G12R	0·0020	0*			Wildtype	
C_96	SUP62	SUP	Pelvic sidewall (L)	Macro	G12V	0·0078	0*			Wildtype	
C_96	SUP62	SUP	Pelvic sidewall (L)	Macro	G12A	0·014	0·072	0*	0*	Wildtype	
C_96	SUP62	SUP	Pelvic sidewall (L)	Macro	G12S	0·035	0·11	0·44	0·19	Wildtype	
C_97	SUP78	SUP	Uterosacral (L)	Macro	G12C	0*	0*			Wildtype	
C_97	SUP78	SUP	Uterosacral (L)	Macro	G12D	0·043	0·0015			Wildtype	
C_97	SUP78	SUP	Uterosacral (L)	Macro	G12R	0*	0*			Wildtype	
C_97	SUP78	SUP	Uterosacral (L)	Macro	G12V	0·0020	0*			Wildtype	
C_97	SUP78	SUP	Uterosacral (L)	Macro	G12A	0*	0*			Wildtype	
C_97	SUP78	SUP	Uterosacral (L)	Macro	G12S	0·031	0·0015			Wildtype	
C_97	SUP106	SUP	Uterus (A)	Macro	G12C	0·0058	0*			Wildtype	
C_97	SUP106	SUP	Uterus (A)	Macro	G12D	0·084	0·0030			Wildtype	
C_97	SUP106	SUP	Uterus (A)	Macro	G12R	0*	0*			Wildtype	
C_97	SUP106	SUP	Uterus (A)	Macro	G12V	0·0036	0·0015			Wildtype	
C_97	SUP106	SUP	Uterus (A)	Macro	G12A	0·0022	0*			Wildtype	
C_97	SUP106	SUP	Uterus (A)	Macro	G12S	0·046	0·0015			Wildtype	
C_98	DIE104	DIE	Periureteric (R)	Macro	G12C	0·010	0*			Wildtype	
C_98	DIE104	DIE	Periureteric (R)	Macro	G12D	0·043	0·061	0·74	0·31	Wildtype	
C_98	DIE104	DIE	Periureteric (R)	Macro	G12R	0·0016	0*			Wildtype	

Case ID	Sample ID	Endo type	Anatomical Location	Enrich. method	Variant tested	Multi. cut-off VAF (%)	Multi. VAF (%)	Indiv. cut-off VAF (%)	Indiv. VAF (%)	Result	*KRAS* Variant
C_98	DIE104	DIE	Periureteric (R)	Macro	G12V	0·010	0*			Wildtype	
C_98	DIE104	DIE	Periureteric (R)	Macro	G12A	0·0046	0*			Wildtype	
C_98	DIE104	DIE	Periureteric (R)	Macro	G12S	0·085	0·012			Wildtype	
C_99	DIE51	DIE	Bladder	Macro	G12C	0·24	0·014			Wildtype	
C_99	DIE51	DIE	Bladder	Macro	G12D	0·24	0·014			Wildtype	
C_99	DIE51	DIE	Bladder	Macro	G12R	0·0048	0*			Wildtype	
C_99	DIE51	DIE	Bladder	Macro	G12V	0·0060	0*			Wildtype	
C_99	DIE51	DIE	Bladder	Macro	G12A	0*	0*			Wildtype	
C_99	DIE51	DIE	Bladder	Macro	G12S	0·23	0·0068			Wildtype	
C_99	DIE52	DIE	Cul-de-sac (L)	Macro	G12C	0·24	0·018			Wildtype	
C_99	DIE52	DIE	Cul-de-sac (L)	Macro	G12D	0·24	0·018			Wildtype	
C_99	DIE52	DIE	Cul-de-sac (L)	Macro	G12R	0·0048	0*			Wildtype	
C_99	DIE52	DIE	Cul-de-sac (L)	Macro	G12V	0·0060	0·085	0·019	0·55	Mutant	G12V
C_99	DIE52	DIE	Cul-de-sac (L)	Macro	G12A	0*	0*			Wildtype	
C_99	DIE52	DIE	Cul-de-sac (L)	Macro	G12S	0·23	0·031			Wildtype	
C_99	SUP79	SUP	Pelvic sidewall (R)	Macro	G12C	0·0058	0*			Wildtype	
C_99	SUP79	SUP	Pelvic sidewall (R)	Macro	G12D	0·084	0·0013			Wildtype	
C_99	SUP79	SUP	Pelvic sidewall (R)	Macro	G12R	0*	0*			Wildtype	
C_99	SUP79	SUP	Pelvic sidewall (R)	Macro	G12V	0·0036	0*			Wildtype	
C_99	SUP79	SUP	Pelvic sidewall (R)	Macro	G12A	0·0022	0*			Wildtype	
C_99	SUP79	SUP	Pelvic sidewall (R)	Macro	G12S	0·046	0*			Wildtype	
C_99	SUP107	SUP	Uterus (A)	Macro	G12C	0*	0*			Wildtype	
C_99	SUP107	SUP	Uterus (A)	Macro	G12D	0·043	0*			Wildtype	
C_99	SUP107	SUP	Uterus (A)	Macro	G12R	0*	0*			Wildtype	
C_99	SUP107	SUP	Uterus (A)	Macro	G12V	0·0020	0*			Wildtype	
C_99	SUP107	SUP	Uterus (A)	Macro	G12A	0*	0*			Wildtype	
C_99	SUP107	SUP	Uterus (A)	Macro	G12S	0·031	0·0029			Wildtype	
C_100	SUP63	SUP	Uterosacral (R)	Macro	G12C	0·0049	0*			Wildtype	

Case ID	Sample ID	Endo type	Anatomical Location	Enrich. method	Variant tested	Multi. cut-off VAF (%)	Multi. VAF (%)	Indiv. cut-off VAF (%)	Indiv. VAF (%)	Result	*KRAS* Variant
C_100	SUP63	SUP	Uterosacral (R)	Macro	G12D	0·012	0·0070			Wildtype	
C_100	SUP63	SUP	Uterosacral (R)	Macro	G12R	0·0020	0*			Wildtype	
C_100	SUP63	SUP	Uterosacral (R)	Macro	G12V	0·0078	0*			Wildtype	
C_100	SUP63	SUP	Uterosacral (R)	Macro	G12A	0·014	0·014	0*	0*	Wildtype	
C_100	SUP63	SUP	Uterosacral (R)	Macro	G12S	0·035	0·028			Wildtype	
C_101	SUP64	SUP	Uterosacral (L)	Macro	G12C	0*	0*			Wildtype	
C_101	SUP64	SUP	Uterosacral (L)	Macro	G12D	0·027	0·0024			Wildtype	
C_101	SUP64	SUP	Uterosacral (L)	Macro	G12R	0*	0*			Wildtype	
C_101	SUP64	SUP	Uterosacral (L)	Macro	G12V	0*	0·0025	0·0065	0*	Wildtype	
C_101	SUP64	SUP	Uterosacral (L)	Macro	G12A	0·0021	0*			Wildtype	
C_101	SUP64	SUP	Uterosacral (L)	Macro	G12S	0·021	0·0049			Wildtype	
C_102	SUP108	SUP	Pelvic sidewall (L)	Macro	G12C	0·0058	0*			Wildtype	
C_102	SUP108	SUP	Pelvic sidewall (L)	Macro	G12D	0·084	0*			Wildtype	
C_102	SUP108	SUP	Pelvic sidewall (L)	Macro	G12R	0*	0*			Wildtype	
C_102	SUP108	SUP	Pelvic sidewall (L)	Macro	G12V	0·0036	0·0044	0·0053	0·0033	Wildtype	
C_102	SUP108	SUP	Pelvic sidewall (L)	Macro	G12A	0·0022	0*			Wildtype	
C_102	SUP108	SUP	Pelvic sidewall (L)	Macro	G12S	0·046	0·0073			Wildtype	
C_102	SUP65	SUP	Uterosacral (R)	Macro	G12C	0*	0*			Wildtype	
C_102	SUP65	SUP	Uterosacral (R)	Macro	G12D	0·027	0·0041			Wildtype	
C_102	SUP65	SUP	Uterosacral (R)	Macro	G12R	0*	0*			Wildtype	
C_102	SUP65	SUP	Uterosacral (R)	Macro	G12V	0*	0·0022	0·0065	0·0037	Wildtype	
C_102	SUP65	SUP	Uterosacral (R)	Macro	G12A	0·0020	0·0020	0*	0·022	Mutant	G12A
C_102	SUP65	SUP	Uterosacral (R)	Macro	G12S	0·021	0·0021			Wildtype	
C_103	SUP66	SUP	Uterosacral (L)	Macro	G12C	0*	0*			Wildtype	
C_103	SUP66	SUP	Uterosacral (L)	Macro	G12D	0·027	0·033	0·73	0·45	Wildtype	
C_103	SUP66	SUP	Uterosacral (L)	Macro	G12R	0*	0*			Wildtype	
C_103	SUP66	SUP	Uterosacral (L)	Macro	G12V	0*	0·0057	0·0065	0*	Wildtype	
C_103	SUP66	SUP	Uterosacral (L)	Macro	G12A	0·0021	0·0028	0*	0*	Wildtype	

Case ID	Sample ID	Endo type	Anatomical Location	Enrich. method	Variant tested	Multi. cut-off VAF (%)	Multi. VAF (%)	Indiv. cut-off VAF (%)	Indiv. VAF (%)	Result	*KRAS* Variant
C_103	SUP66	SUP	Uterosacral (L)	Macro	G12S	0·021	0·020			Wildtype	
C_104	SUP67	SUP	Cul-de-sac	Macro	G12C	0*	0*			Wildtype	
C_104	SUP67	SUP	Cul-de-sac	Macro	G12D	0·027	0·066	0·73	0·45	Wildtype	
C_104	SUP67	SUP	Cul-de-sac	Macro	G12R	0*	0*			Wildtype	
C_104	SUP67	SUP	Cul-de-sac	Macro	G12V	0*	0·0032	0·0065	0·0037	Wildtype	
C_104	SUP67	SUP	Cul-de-sac	Macro	G12A	0·0021	0*			Wildtype	
C_104	SUP67	SUP	Cul-de-sac	Macro	G12S	0·021	0·016			Wildtype	
C_105	DIE46	DIE	Periureteric (R)	Macro	G12C	0·24	0·014			Wildtype	
C_105	DIE46	DIE	Periureteric (R)	Macro	G12D	0·24	0·014			Wildtype	
C_105	DIE46	DIE	Periureteric (R)	Macro	G12R	0·0048	0*			Wildtype	
C_105	DIE46	DIE	Periureteric (R)	Macro	G12V	0·0060	0*			Wildtype	
C_105	DIE46	DIE	Periureteric (R)	Macro	G12A	0*	0*			Wildtype	
C_105	DIE46	DIE	Periureteric (R)	Macro	G12S	0·23	0·007			Wildtype	
C_105	DIE47	DIE	Pelvic sidewall (L)	Macro	G12C	0·24	0·0099			Wildtype	
C_105	DIE47	DIE	Pelvic sidewall (L)	Macro	G12D	0·24	0·0099			Wildtype	
C_105	DIE47	DIE	Pelvic sidewall (L)	Macro	G12R	0·0048	0*			Wildtype	
C_105	DIE47	DIE	Pelvic sidewall (L)	Macro	G12V	0·0060	0*			Wildtype	
C_105	DIE47	DIE	Pelvic sidewall (L)	Macro	G12A	0*	0*			Wildtype	
C_105	DIE47	DIE	Pelvic sidewall (L)	Macro	G12S	0·23	0·012			Wildtype	
C_105	DIE48	DIE	Periureteric (R)	Macro	G12C	0·24	0·20			Wildtype	
C_105	DIE48	DIE	Periureteric (R)	Macro	G12D	0·24	0·20	0·35	0·77	Mutant	G12D
C_105	DIE48	DIE	Periureteric (R)	Macro	G12R	0·0048	0*			Wildtype	
C_105	DIE48	DIE	Periureteric (R)	Macro	G12V	0·0060	0·0068	0·010	0·0075	Wildtype	
C_105	DIE48	DIE	Periureteric (R)	Macro	G12A	0*	0*			Wildtype	
C_105	DIE48	DIE	Periureteric (R)	Macro	G12S	0·23	0·034			Wildtype	
C_105	OMA34	OMA	Ovary (L)	Macro	G12C	0*	0*			Wildtype	
C_105	OMA34	OMA	Ovary (L)	Macro	G12D	0·043	0·0071			Wildtype	
C_105	OMA34	OMA	Ovary (L)	Macro	G12R	0*	0*			Wildtype	

Case ID	Sample ID	Endo type	Anatomical Location	Enrich. method	Variant tested	Multi. cut-off VAF (%)	Multi. VAF (%)	Indiv. cut-off VAF (%)	Indiv. VAF (%)	Result	*KRAS* Variant
C_105	OMA34	OMA	Ovary (L)	Macro	G12V	0·0020	0·0035	0·0039	0·024	Mutant	G12V
C_105	OMA34	OMA	Ovary (L)	Macro	G12A	0*	0*			Wildtype	
C_105	OMA34	OMA	Ovary (L)	Macro	G12S	0·031	0·013			Wildtype	
C_106	SUP68	SUP	Uterosacral (R)	Macro	G12C	0*	0*			Wildtype	
C_106	SUP68	SUP	Uterosacral (R)	Macro	G12D	0·027	0·0030			Wildtype	
C_106	SUP68	SUP	Uterosacral (R)	Macro	G12R	0*	0*			Wildtype	
C_106	SUP68	SUP	Uterosacral (R)	Macro	G12V	0*	0·0016	0·0065	0·0017	Wildtype	
C_106	SUP68	SUP	Uterosacral (R)	Macro	G12A	0·0021	0*			Wildtype	
C_106	SUP68	SUP	Uterosacral (R)	Macro	G12S	0·021	0·0081			Wildtype	
C_107	DIE64	DIE	Periureteric (L)	Macro	G12C	0·24	0·011			Wildtype	
C_107	DIE64	DIE	Periureteric (L)	Macro	G12D	0·24	0·011			Wildtype	
C_107	DIE64	DIE	Periureteric (L)	Macro	G12R	0·0048	0*			Wildtype	
C_107	DIE64	DIE	Periureteric (L)	Macro	G12V	0·0060	0*			Wildtype	
C_107	DIE64	DIE	Periureteric (L)	Macro	G12A	0*	0*			Wildtype	
C_107	DIE64	DIE	Periureteric (L)	Macro	G12S	0·23	0*			Wildtype	
C_107	DIE65	DIE	Periureteric (L)	Macro	G12C	0·24	0·0025			Wildtype	
C_107	DIE65	DIE	Periureteric (L)	Macro	G12D	0·24	0·0025			Wildtype	
C_107	DIE65	DIE	Periureteric (L)	Macro	G12R	0·0048	0*			Wildtype	
C_107	DIE65	DIE	Periureteric (L)	Macro	G12V	0·0060	0·0025			Wildtype	
C_107	DIE65	DIE	Periureteric (L)	Macro	G12A	0*	0*			Wildtype	
C_107	DIE65	DIE	Periureteric (L)	Macro	G12S	0·23	0·0076			Wildtype	
C_107	OMA35	OMA	Ovary (L)	Macro	G12C	0*	0*			Wildtype	
C_107	OMA35	OMA	Ovary (L)	Macro	G12D	0·043	0*			Wildtype	
C_107	OMA35	OMA	Ovary (L)	Macro	G12R	0*	0*			Wildtype	
C_107	OMA35	OMA	Ovary (L)	Macro	G12V	0·0020	0*			Wildtype	
C_107	OMA35	OMA	Ovary (L)	Macro	G12A	0*	0*			Wildtype	
C_107	OMA35	OMA	Ovary (L)	Macro	G12S	0·031	0·0016			Wildtype	
C_107	SUP69	SUP	Fallopian tube (L)	Macro	G12C	0*	0*			Wildtype	

Case ID	Sample ID	Endo type	Anatomical Location	Enrich. method	Variant tested	Multi. cut-off VAF (%)	Multi. VAF (%)	Indiv. cut-off VAF (%)	Indiv. VAF (%)	Result	*KRAS* Variant
C_107	SUP69	SUP	Fallopian tube (L)	Macro	G12D	0·027	0·0072			Wildtype	
C_107	SUP69	SUP	Fallopian tube (L)	Macro	G12R	0*	0*			Wildtype	
C_107	SUP69	SUP	Fallopian tube (L)	Macro	G12V	0*	0·0019	0·0065	0·0064	Wildtype	
C_107	SUP69	SUP	Fallopian tube (L)	Macro	G12A	0·0021	0*			Wildtype	
C_107	SUP69	SUP	Fallopian tube (L)	Macro	G12S	0·021	0·0039			Wildtype	
C_108	OMA36	OMA	Ovary (NS)	Macro	G12C	0*	0*			Wildtype	
C_108	OMA36	OMA	Ovary (NS)	Macro	G12D	0·043	0·0065			Wildtype	
C_108	OMA36	OMA	Ovary (NS)	Macro	G12R	0*	0*			Wildtype	
C_108	OMA36	OMA	Ovary (NS)	Macro	G12V	0·0020	0*			Wildtype	
C_108	OMA36	OMA	Ovary (NS)	Macro	G12A	0*	0*			Wildtype	
C_108	OMA36	OMA	Ovary (NS)	Macro	G12S	0·031	0·0033			Wildtype	
C_109	DIE70	DIE	Cul-de-sac (L)	Macro	G12C	0·24	0·17			Wildtype	
C_109	DIE70	DIE	Cul-de-sac (L)	Macro	G12D	0·24	0·17	0·35	0·39	Mutant	G12D
C_109	DIE70	DIE	Cul-de-sac (L)	Macro	G12R	0·0048	0*			Wildtype	
C_109	DIE70	DIE	Cul-de-sac (L)	Macro	G12V	0·0060	0*			Wildtype	
C_109	DIE70	DIE	Cul-de-sac (L)	Macro	G12A	0*	0*			Wildtype	
C_109	DIE70	DIE	Cul-de-sac (L)	Macro	G12S	0·23	0·21	0·67	0·45	Wildtype	
C_109	OMA37	OMA	Ovary (L)	Macro	G12C	0*	0*			Wildtype	
C_109	OMA37	OMA	Ovary (L)	Macro	G12D	0·043	0·063	1·5	0·76	Wildtype	
C_109	OMA37	OMA	Ovary (L)	Macro	G12R	0*	0*			Wildtype	
C_109	OMA37	OMA	Ovary (L)	Macro	G12V	0·0020	0·027	0·0039	0*	Wildtype	
C_109	OMA37	OMA	Ovary (L)	Macro	G12A	0*	0·43	0·0022	3·7	Mutant	G12A
C_109	OMA37	OMA	Ovary (L)	Macro	G12S	0·031	0·077	0·78	0·43	Wildtype	
C_110	SUP38	SUP	Cul-de-sac	Macro	G12C	0·0049	0*			Wildtype	
C_110	SUP38	SUP	Cul-de-sac	Macro	G12D	0·012	0·0036			Wildtype	
C_110	SUP38	SUP	Cul-de-sac	Macro	G12R	0·0020	0*			Wildtype	
C_110	SUP38	SUP	Cul-de-sac	Macro	G12V	0·0078	0·0035	0·0097	0·0088	Wildtype	
C_110	SUP38	SUP	Cul-de-sac	Macro	G12A	0·014	0*			Wildtype	

Case ID	Sample ID	Endo type	Anatomical Location	Enrich. method	Variant tested	Multi. cut-off VAF (%)	Multi. VAF (%)	Indiv. cut-off VAF (%)	Indiv. VAF (%)	Result	*KRAS* Variant
C_110	SUP38	SUP	Cul-de-sac	Macro	G12S	0·035	0·0073			Wildtype	
C_111	SUP109	SUP	Uterosacral (L)	Macro	G12C	0·0058	0*			Wildtype	
C_111	SUP109	SUP	Uterosacral (L)	Macro	G12D	0·084	0·083	0·98	0·31	Wildtype	
C_111	SUP109	SUP	Uterosacral (L)	Macro	G12R	0*	0*			Wildtype	
C_111	SUP109	SUP	Uterosacral (L)	Macro	G12V	0·0036	0·0052	0·0053	0·017	Mutant	G12V
C_111	SUP109	SUP	Uterosacral (L)	Macro	G12A	0·0022	0*			Wildtype	
C_111	SUP109	SUP	Uterosacral (L)	Macro	G12S	0·046	0·20	0·90	0·72	Wildtype	
C_111	SUP39	SUP	Cul-de-sac (R)	Macro	G12C	0*	0*			Wildtype	
C_111	SUP39	SUP	Cul-de-sac (R)	Macro	G12D	0·019	0·18	0·99	0·51	Wildtype	
C_111	SUP39	SUP	Cul-de-sac (R)	Macro	G12R	0*	0*			Wildtype	
C_111	SUP39	SUP	Cul-de-sac (R)	Macro	G12V	0·0016	0*			Wildtype	
C_111	SUP39	SUP	Cul-de-sac (R)	Macro	G12A	0·0016	0*			Wildtype	
C_111	SUP39	SUP	Cul-de-sac (R)	Macro	G12S	0·030	0·42	1·0	0·61	Wildtype	
C_112	SUP110	SUP	Cul-de-sac (R)	Macro	G12C	0·0058	0*			Wildtype	
C_112	SUP110	SUP	Cul-de-sac (R)	Macro	G12D	0·084	0·019			Wildtype	
C_112	SUP110	SUP	Cul-de-sac (R)	Macro	G12R	0*	0*			Wildtype	
C_112	SUP110	SUP	Cul-de-sac (R)	Macro	G12V	0·0036	0·0083	0·0053	0·0036	Wildtype	
C_112	SUP110	SUP	Cul-de-sac (R)	Macro	G12A	0·0022	0*			Wildtype	
C_112	SUP110	SUP	Cul-de-sac (R)	Macro	G12S	0·046	0·069	0·90	0·52	Wildtype	
C_112	SUP45	SUP	Pelvic sidewall (L)	Macro	G12C	0*	0*			Wildtype	
C_112	SUP45	SUP	Pelvic sidewall (L)	Macro	G12D	0·019	0·018			Wildtype	
C_112	SUP45	SUP	Pelvic sidewall (L)	Macro	G12R	0*	0*			Wildtype	
C_112	SUP45	SUP	Pelvic sidewall (L)	Macro	G12V	0·0016	0*			Wildtype	
C_112	SUP45	SUP	Pelvic sidewall (L)	Macro	G12A	0·0016	0*			Wildtype	
C_112	SUP45	SUP	Pelvic sidewall (L)	Macro	G12S	0·030	0·095	1·0	0·35	Wildtype	
C_113	DIE71	DIE	Appendix	Macro	G12C	0·24	0*			Wildtype	
C_113	DIE71	DIE	Appendix	Macro	G12D	0·24	0*			Wildtype	
C_113	DIE71	DIE	Appendix	Macro	G12R	0·0048	0*			Wildtype	

Case ID	Sample ID	Endo type	Anatomical Location	Enrich. method	Variant tested	Multi. cut-off VAF (%)	Multi. VAF (%)	Indiv. cut-off VAF (%)	Indiv. VAF (%)	Result	*KRAS* Variant
C_113	DIE71	DIE	Appendix	Macro	G12V	0·0060	0·0030			Wildtype	
C_113	DIE71	DIE	Appendix	Macro	G12A	0*	0*			Wildtype	
C_113	DIE71	DIE	Appendix	Macro	G12S	0·23	0·0093			Wildtype	
C_113	SUP111	SUP	Pelvic sidewall (L)	Macro	G12C	0·0058	0*			Wildtype	
C_113	SUP111	SUP	Pelvic sidewall (L)	Macro	G12D	0·084	0·0015			Wildtype	
C_113	SUP111	SUP	Pelvic sidewall (L)	Macro	G12R	0*	0*			Wildtype	
C_113	SUP111	SUP	Pelvic sidewall (L)	Macro	G12V	0·0036	0·0030			Wildtype	
C_113	SUP111	SUP	Pelvic sidewall (L)	Macro	G12A	0·0022	0*			Wildtype	
C_113	SUP111	SUP	Pelvic sidewall (L)	Macro	G12S	0·046	0·0060			Wildtype	
C_113	SUP40	SUP	Rectum (L)	Macro	G12C	0*	0*			Wildtype	
C_113	SUP40	SUP	Rectum (L)	Macro	G12D	0·019	0·019	0·99	0·73	Wildtype	
C_113	SUP40	SUP	Rectum (L)	Macro	G12R	0*	0*			Wildtype	
C_113	SUP40	SUP	Rectum (L)	Macro	G12V	0·0016	0·022	0·017	1·5	Mutant	G12V
C_113	SUP40	SUP	Rectum (L)	Macro	G12A	0·0016	0·0024	0·0044	0·025	Mutant	G12A
C_113	SUP40	SUP	Rectum (L)	Macro	G12S	0·030	0·0024			Wildtype	
C_114	DIE15	DIE	Pelvic sidewall (R)	Macro	G12C	0·013	0*			Wildtype	
C_114	DIE15	DIE	Pelvic sidewall (R)	Macro	G12D	0·056	0·0067			Wildtype	
C_114	DIE15	DIE	Pelvic sidewall (R)	Macro	G12R	0*	0*			Wildtype	
C_114	DIE15	DIE	Pelvic sidewall (R)	Macro	G12V	0·0060	0·0058			Wildtype	
C_114	DIE15	DIE	Pelvic sidewall (R)	Macro	G12A	0·0060	0*			Wildtype	
C_114	DIE15	DIE	Pelvic sidewall (R)	Macro	G12S	0·0020	0·061	0·67	0·44	Wildtype	
C_114	SUP41	SUP	Pelvic sidewall (L)	Macro	G12C	0·0041	0*			Wildtype	
C_114	SUP41	SUP	Pelvic sidewall (L)	Macro	G12D	0·018	0·16	0·73	0·68	Wildtype	
C_114	SUP41	SUP	Pelvic sidewall (L)	Macro	G12R	0·0014	0*			Wildtype	
C_114	SUP41	SUP	Pelvic sidewall (L)	Macro	G12V	0·00070	0*			Wildtype	
C_114	SUP41	SUP	Pelvic sidewall (L)	Macro	G12A	0*	0*			Wildtype	
C_114	SUP41	SUP	Pelvic sidewall (L)	Macro	G12S	0·033	0·087	0·44	0·19	Wildtype	
C_115	DIE27	DIE	Uterosacral (R)	Macro	G12C	0·013	0*			Wildtype	

Case ID	Sample ID	Endo type	Anatomical Location	Enrich. method	Variant tested	Multi. cut-off VAF (%)	Multi. VAF (%)	Indiv. cut-off VAF (%)	Indiv. VAF (%)	Result	*KRAS* Variant
C_115	DIE27	DIE	Uterosacral (R)	Macro	G12D	0·056	0·010			Wildtype	
C_115	DIE27	DIE	Uterosacral (R)	Macro	G12R	0*	0*			Wildtype	
C_115	DIE27	DIE	Uterosacral (R)	Macro	G12V	0·0060	0·0042			Wildtype	
C_115	DIE27	DIE	Uterosacral (R)	Macro	G12A	0·0060	0*			Wildtype	
C_115	DIE27	DIE	Uterosacral (R)	Macro	G12S	0·0020	0·033	0·67	0·26	Wildtype	
C_115	OMA38	OMA	Ovary (L)	Macro	G12C	0*	0·0051	0·030	0*	Wildtype	
C_115	OMA38	OMA	Ovary (L)	Macro	G12D	0·043	0·092	1·3	0·34	Wildtype	
C_115	OMA38	OMA	Ovary (L)	Macro	G12R	0*	0*			Wildtype	
C_115	OMA38	OMA	Ovary (L)	Macro	G12V	0·0020	0·010	0·0039	0·034	Mutant	G12V
C_115	OMA38	OMA	Ovary (L)	Macro	G12A	0*	0·0051	0·0022	0*	Wildtype	
C_115	OMA38	OMA	Ovary (L)	Macro	G12S	0·031	0·17	0·78	0·42	Wildtype	
C_116	SUP112	SUP	Uterosacral (L)	Macro	G12C	0·0058	0*			Wildtype	
C_116	SUP112	SUP	Uterosacral (L)	Macro	G12D	0·084	0*			Wildtype	
C_116	SUP112	SUP	Uterosacral (L)	Macro	G12R	0*	0*			Wildtype	
C_116	SUP112	SUP	Uterosacral (L)	Macro	G12V	0·0036	0·0034	0·0053	0·0028	Wildtype	
C_116	SUP112	SUP	Uterosacral (L)	Macro	G12A	0·0022	0·0034	0·0015	0·023	Mutant	G12A
C_116	SUP112	SUP	Uterosacral (L)	Macro	G12S	0·046	0·0067			Wildtype	
C_116	SUP70	SUP	Pelvic sidewall (L)	Macro	G12C	0*	0*			Wildtype	
C_116	SUP70	SUP	Pelvic sidewall (L)	Macro	G12D	0·027	0*			Wildtype	
C_116	SUP70	SUP	Pelvic sidewall (L)	Macro	G12R	0*	0*			Wildtype	
C_116	SUP70	SUP	Pelvic sidewall (L)	Macro	G12V	0*	0*			Wildtype	
C_116	SUP70	SUP	Pelvic sidewall (L)	Macro	G12A	0·0021	0*			Wildtype	
C_116	SUP70	SUP	Pelvic sidewall (L)	Macro	G12S	0·021	0·0075			Wildtype	
C_117	DIE39	DIE	Uterosacral (R)	LCM	G12C	0·010	0·0092			Wildtype	
C_117	DIE39	DIE	Uterosacral (R)	LCM	G12D	0·043	0·35	0·74	0·79	Mutant	G12D
C_117	DIE39	DIE	Uterosacral (R)	LCM	G12R	0·0016	0*			Wildtype	
C_117	DIE39	DIE	Uterosacral (R)	LCM	G12V	0·010	0·018	0·010	0*	Wildtype	
C_117	DIE39	DIE	Uterosacral (R)	LCM	G12A	0·0046	0*			Wildtype	

Case ID	Sample ID	Endo type	Anatomical Location	Enrich. method	Variant tested	Multi. cut-off VAF (%)	Multi. VAF (%)	Indiv. cut-off VAF (%)	Indiv. VAF (%)	Result	*KRAS* Variant
C_117	DIE39	DIE	Uterosacral (R)	LCM	G12S	0·085	0·018			Wildtype	
C_117	DIE40	DIE	Uterosacral (R)	Macro	G12C	0·34	0·56	0·030	0*	Wildtype	
C_117	DIE40	DIE	Uterosacral (R)	Macro	G12D	0·24	0·57	0·35	1·0	Mutant	G12D
C_117	DIE40	DIE	Uterosacral (R)	Macro	G12R	0·0048	0*			Wildtype	
C_117	DIE40	DIE	Uterosacral (R)	Macro	G12V	0·0060	0*			Wildtype	
C_117	DIE40	DIE	Uterosacral (R)	Macro	G12A	0*	0*			Wildtype	
C_117	DIE40	DIE	Uterosacral (R)	Macro	G12S	0·43	0·023			Wildtype	
C_117	DIE41	DIE	Uterosacral (L)	Macro	G12C	0·24	0·032			Wildtype	
C_117	DIE41	DIE	Uterosacral (L)	Macro	G12D	0·24	0·032			Wildtype	
C_117	DIE41	DIE	Uterosacral (L)	Macro	G12R	0·0048	0*			Wildtype	
C_117	DIE41	DIE	Uterosacral (L)	Macro	G12V	0·0060	0*			Wildtype	
C_117	DIE41	DIE	Uterosacral (L)	Macro	G12A	0*	0*			Wildtype	
C_117	DIE41	DIE	Uterosacral (L)	Macro	G12S	0·23	0·053			Wildtype	
C_117	SUP42	SUP	Cul-de-sac (L)	Macro	G12C	0·0041	0*			Wildtype	
C_117	SUP42	SUP	Cul-de-sac (L)	Macro	G12D	0·018	0·049	0·73	0·42	Wildtype	
C_117	SUP42	SUP	Cul-de-sac (L)	Macro	G12R	0·0014	0*			Wildtype	
C_117	SUP42	SUP	Cul-de-sac (L)	Macro	G12V	0·00070	0·022	0·0060	0·18	Mutant	G12V
C_117	SUP42	SUP	Cul-de-sac (L)	Macro	G12A	0*	0*			Wildtype	
C_117	SUP42	SUP	Cul-de-sac (L)	Macro	G12S	0·033	0·022			Wildtype	
C_118	DIE37	DIE	Uterosacral	LCM	G12C	0·010	0*			Wildtype	
C_118	DIE37	DIE	Uterosacral	LCM	G12D	0·043	0·035			Wildtype	
C_118	DIE37	DIE	Uterosacral	LCM	G12R	0·0016	0*			Wildtype	
C_118	DIE37	DIE	Uterosacral	LCM	G12V	0·010	0*			Wildtype	
C_118	DIE37	DIE	Uterosacral	LCM	G12A	0·0046	0*			Wildtype	
C_118	DIE37	DIE	Uterosacral	LCM	G12S	0·085	0·017			Wildtype	
C_118	DIE38	DIE	Uterosacral (L)	Macro	G12C	0·24	0·019			Wildtype	
C_118	DIE38	DIE	Uterosacral (L)	Macro	G12D	0·24	0·019	0·35	0·19	Wildtype	
C_118	DIE38	DIE	Uterosacral (L)	Macro	G12R	0·0048	0*			Wildtype	

Case ID	Sample ID	Endo type	Anatomical Location	Enrich. method	Variant tested	Multi. cut-off VAF (%)	Multi. VAF (%)	Indiv. cut-off VAF (%)	Indiv. VAF (%)	Result	*KRAS* Variant
C_118	DIE38	DIE	Uterosacral (L)	Macro	G12V	0·0060	0·0042			Wildtype	
C_118	DIE38	DIE	Uterosacral (L)	Macro	G12A	0*	0*			Wildtype	
C_118	DIE38	DIE	Uterosacral (L)	Macro	G12S	0·23	0·0021			Wildtype	
C_118	OMA39	OMA	Ovary (L)	Macro	G12C	0*	0*			Wildtype	
C_118	OMA39	OMA	Ovary (L)	Macro	G12D	0·043	0·014			Wildtype	
C_118	OMA39	OMA	Ovary (L)	Macro	G12R	0*	0*			Wildtype	
C_118	OMA39	OMA	Ovary (L)	Macro	G12V	0·0020	0·0046	0·0039	0·0020	Wildtype	
C_118	OMA39	OMA	Ovary (L)	Macro	G12A	0*	0*			Wildtype	
C_118	OMA39	OMA	Ovary (L)	Macro	G12S	0·031	0·023			Wildtype	
C_119	DIE68	DIE	Bladder (L)	Macro	G12C	0·24	0·040			Wildtype	
C_119	DIE68	DIE	Bladder (L)	Macro	G12D	0·24	0·040			Wildtype	
C_119	DIE68	DIE	Bladder (L)	Macro	G12R	0·0048	0*			Wildtype	
C_119	DIE68	DIE	Bladder (L)	Macro	G12V	0·0060	0*			Wildtype	
C_119	DIE68	DIE	Bladder (L)	Macro	G12A	0*	0*			Wildtype	
C_119	DIE68	DIE	Bladder (L)	Macro	G12S	0·23	0·35	0·67	0·47	Wildtype	
C_119	DIE69	DIE	Bladder (L)	Macro	G12C	0·34	0*			Wildtype	
C_119	DIE69	DIE	Bladder (L)	Macro	G12D	0·24	0·033			Wildtype	
C_119	DIE69	DIE	Bladder (L)	Macro	G12R	0·0048	0*			Wildtype	
C_119	DIE69	DIE	Bladder (L)	Macro	G12V	0·0060	0*			Wildtype	
C_119	DIE69	DIE	Bladder (L)	Macro	G12A	0*	0*			Wildtype	
C_119	DIE69	DIE	Bladder (L)	Macro	G12S	0·23	0·033			Wildtype	
C_119	OMA40	OMA	Ovary (L)	Macro	G12C	0*	0*			Wildtype	
C_119	OMA40	OMA	Ovary (L)	Macro	G12D	0·043	0·37	1·3	0·39	Wildtype	
C_119	OMA40	OMA	Ovary (L)	Macro	G12R	0*	0*			Wildtype	
C_119	OMA40	OMA	Ovary (L)	Macro	G12V	0·0020	0*			Wildtype	
C_119	OMA40	OMA	Ovary (L)	Macro	G12A	0*	0·010	0·0022	0*	Wildtype	
C_119	OMA40	OMA	Ovary (L)	Macro	G12S	0·031	0·23	0·78	0·59	Wildtype	
C_119	SUP113	SUP	Cul-de-sac (L)	Macro	G12C	0·0058	0*			Wildtype	

Case ID	Sample ID	Endo type	Anatomical Location	Enrich. method	Variant tested	Multi. cut-off VAF (%)	Multi. VAF (%)	Indiv. cut-off VAF (%)	Indiv. VAF (%)	Result	*KRAS* Variant
C_119	SUP113	SUP	Cul-de-sac (L)	Macro	G12D	0·084	0·058	0·98	0·24	Wildtype	
C_119	SUP113	SUP	Cul-de-sac (L)	Macro	G12R	0*	0*			Wildtype	
C_119	SUP113	SUP	Cul-de-sac (L)	Macro	G12V	0·0036	0*			Wildtype	
C_119	SUP113	SUP	Cul-de-sac (L)	Macro	G12A	0·0022	0*			Wildtype	
C_119	SUP113	SUP	Cul-de-sac (L)	Macro	G12S	0·046	0·13	0·90	0·66	Wildtype	
C_119	SUP71	SUP	Pelvic sidewall (R)	Macro	G12C	0*	0*			Wildtype	
C_119	SUP71	SUP	Pelvic sidewall (R)	Macro	G12D	0·027	0·0042			Wildtype	
C_119	SUP71	SUP	Pelvic sidewall (R)	Macro	G12R	0*	0*			Wildtype	
C_119	SUP71	SUP	Pelvic sidewall (R)	Macro	G12V	0*	0·0087	0·0065	0*	Wildtype	
C_119	SUP71	SUP	Pelvic sidewall (R)	Macro	G12A	0·0021	0*			Wildtype	
C_119	SUP71	SUP	Pelvic sidewall (R)	Macro	G12S	0·021	0·0022			Wildtype	
C_120	SUP43	SUP	Cul-de-sac (L)	Macro	G12C	0*	0*			Wildtype	
C_120	SUP43	SUP	Cul-de-sac (L)	Macro	G12D	0·019	0·060	0·99	0·32	Wildtype	
C_120	SUP43	SUP	Cul-de-sac (L)	Macro	G12R	0*	0*			Wildtype	
C_120	SUP43	SUP	Cul-de-sac (L)	Macro	G12V	0·0016	0*			Wildtype	
C_120	SUP43	SUP	Cul-de-sac (L)	Macro	G12A	0·0016	0*			Wildtype	
C_120	SUP43	SUP	Cul-de-sac (L)	Macro	G12S	0·030	0·030	1·0	0·13	Wildtype	
C_121	DIE11	DIE	Fallopian tube (L)	Macro	G12C	0·34	1·2	0·030	0*	Wildtype	
C_121	DIE11	DIE	Fallopian tube (L)	Macro	G12D	0·34	1·2	0·35	1·7	Mutant	G12D
C_121	DIE11	DIE	Fallopian tube (L)	Macro	G12R	0·0048	0*			Wildtype	
C_121	DIE11	DIE	Fallopian tube (L)	Macro	G12V	0·0060	0*			Wildtype	
C_121	DIE11	DIE	Fallopian tube (L)	Macro	G12A	0*	0*			Wildtype	
C_121	DIE11	DIE	Fallopian tube (L)	Macro	G12S	0·43	1·5	0·67	1·7	Mutant	G12S
C_121	DIE12	DIE	Uterosacral (L)	Macro	G12C	0·24	0·12			Wildtype	
C_121	DIE12	DIE	Uterosacral (L)	Macro	G12D	0·24	0·12	0·35	0·39	Mutant	G12D
C_121	DIE12	DIE	Uterosacral (L)	Macro	G12R	0·0048	0*			Wildtype	
C_121	DIE12	DIE	Uterosacral (L)	Macro	G12V	0·0060	0*			Wildtype	
C_121	DIE12	DIE	Uterosacral (L)	Macro	G12A	0*	0*			Wildtype	

Case ID	Sample ID	Endo type	Anatomical Location	Enrich. method	Variant tested	Multi. cut-off VAF (%)	Multi. VAF (%)	Indiv. cut-off VAF (%)	Indiv. VAF (%)	Result	*KRAS* Variant
C_121	DIE12	DIE	Uterosacral (L)	Macro	G12S	0·23	0·019			Wildtype	
C_121	DIE13	DIE	Uterosacral (L)	Macro	G12C	0·24	0·071			Wildtype	
C_121	DIE13	DIE	Uterosacral (L)	Macro	G12D	0·24	0·071	0·35	0·44	Mutant	G12D
C_121	DIE13	DIE	Uterosacral (L)	Macro	G12R	0·0048	0*			Wildtype	
C_121	DIE13	DIE	Uterosacral (L)	Macro	G12V	0·0060	0*			Wildtype	
C_121	DIE13	DIE	Uterosacral (L)	Macro	G12A	0*	0*			Wildtype	
C_121	DIE13	DIE	Uterosacral (L)	Macro	G12S	0·23	0·034			Wildtype	
C_121	DIE14	DIE	Cul-de-sac	LCM	G12C	0·010	0*			Wildtype	
C_121	DIE14	DIE	Cul-de-sac	LCM	G12D	0·043	0·0024			Wildtype	
C_121	DIE14	DIE	Cul-de-sac	LCM	G12R	0·0016	0*			Wildtype	
C_121	DIE14	DIE	Cul-de-sac	LCM	G12V	0·010	0*			Wildtype	
C_121	DIE14	DIE	Cul-de-sac	LCM	G12A	0·0046	0*			Wildtype	
C_121	DIE14	DIE	Cul-de-sac	LCM	G12S	0·085	0·0024			Wildtype	
C_121	OMA41	OMA	Ovary (L)	Macro	G12C	0*	0*			Wildtype	
C_121	OMA41	OMA	Ovary (L)	Macro	G12D	0·043	0·0029			Wildtype	
C_121	OMA41	OMA	Ovary (L)	Macro	G12R	0*	0*			Wildtype	
C_121	OMA41	OMA	Ovary (L)	Macro	G12V	0·0020	0·0014			Wildtype	
C_121	OMA41	OMA	Ovary (L)	Macro	G12A	0*	0*			Wildtype	
C_121	OMA41	OMA	Ovary (L)	Macro	G12S	0·031	0·0030			Wildtype	
C_122	SUP44	SUP	Cul-de-sac	Macro	G12C	0*	0*			Wildtype	
C_122	SUP44	SUP	Cul-de-sac	Macro	G12D	0·019	0*			Wildtype	
C_122	SUP44	SUP	Cul-de-sac	Macro	G12R	0*	0*			Wildtype	
C_122	SUP44	SUP	Cul-de-sac	Macro	G12V	0·0016	0*			Wildtype	
C_122	SUP44	SUP	Cul-de-sac	Macro	G12A	0·0016	0*			Wildtype	
C_122	SUP44	SUP	Cul-de-sac	Macro	G12S	0·030	0·0088			Wildtype	

* indicates true zeros (0). i.e., no mutant droplets were present. Enrich.=enrichment; Multi.=multiplex; Indiv.=individual; DIE=deep infiltrating endometriosis; OMA=endometrioma; SUP=superficial; L=left; R=right; A=anterior; P=posterior; B=bilateral; NS=non-specific; Macro=manual needle microdissection; LCM=laser capture microdissection.

Table S6. The association between follow-up pain scores and *KRAS* mutation status, controlling for baseline pain severity - linear regression.

Predictors	Unstandardized B	Lower	Upper	P value
**Dysmenorrhea at follow-up**				
KRAS mutation present	0·74	-0·78	2·27	0·33
Dysmenorrhea at baseline	0·28	-0·10	0·58	<0·001
**Deep Dyspareunia at follow-up**				
KRAS mutation present	-0·17	-1·45	1·11	0·79
Deep dyspareunia at baseline	0·42	0·22	0·61	<0·001
**Dyschezia at follow-up**				
KRAS mutation present	-0·40	-1·31	0·51	0·38
Dyschezia at baseline	0·35	0·22	0·49	<0·001
**Chronic Pelvic Pain at follow-up**				
KRAS mutation present	-0·21	-1·45	1·021	0·73
Chronic pelvic pain at baseline	0·31	0·082	0·54	0·008

Linear regression for each pain type at follow-up, showed that baseline pain score was as a significant predictor, but not *KRAS* mutation.
